# Vlasouliolides A-D, four rare C_17_/C_15_ sesquiterpene lactone dimers with potential anti-inflammatory activity from *Vladimiria souliei*

**DOI:** 10.1038/srep43837

**Published:** 2017-03-03

**Authors:** Li-Ping Chen, Guo-Zhen Wu, Jian-Ping Zhang, Ji Ye, Qing-Xin Liu, Yun-Heng Shen, Hui-Liang Li, Wei-Dong Zhang

**Affiliations:** 1 Department of Phytochemistry, School of Pharmacy, Second Military Medical University, Shanghai 200433, P. R. China; 2 Shanghai Institute of Pharmaceutical Industry, Shanghai 200040, P. R. China

## Abstract

Vlasouliolides A-D (**1**–**4**), four rare sesquiterpene lactone dimers, were isolated from *Vladimiria souliei*. The common structural characteristic of **1**–**4** is the C_32_ skeleton comprising two sesquiterpene lactone units linked by a C11-C13′ single bond with one acetyl connected to the C-13 position of one of the two sesquiterpene lactone units. The stereochemistries of **1**–**4** were assigned by a combination of NOESY correlations and Cu-Κ*α* X-ray crystallographic analyses. Compounds **1**–**4** strongly inhibited the production of NO in LPS-stimulated RAW 264.7 cells. Furthermore, **1** and **2** inhibited the activation of NF-*κ*B in LPS-induced 293T cells.

Naturally occurring sesquiterpene lactone dimers (SLDs) are a type of complex found in natural products with many pharmacological activities^1^,^2^. Since the first SLD absinthin was isolated from *Artemisia absinthium* in 1953^3^, more than 160 SLDs have been obtained. To the best of our knowledge, most of the published SLDs have C_30_ cores derived from two C_15_ sesquiterpenoid units^4^,^5^,^6^.

The genus *Vladimiria,* belonging to the family of Asteraceae, comprises approximately 12 species that are mainly distributed in the Sichuan Province, China^7^. Sesquiterpenes, as major constituents isolated from the *Vladimiria* species, possess various types of structures including guaianolide, carabrane, eudesmane and germacrane sesquiterpenes^8^,^9^,^10^,^11^,^12^. The plant of *Vladimiria souliei*, as a traditional Chinese medicine, has been used for relieving pain and stomach diseases since ancient times^7^,^13^. Additionally, the chemical constituents from the roots of *V. souliei* exhibited significant antimicrobial, antitumor and inhibitory effects on NO production activities^10^,^13^. In the course of our investigation on structurally novel SLDs from the family of Asteraceae, four rare SLDs with C_32_ cores were discovered from *Vladimiria souliei*, and they were designated vlasouliolides A-D (**1**–**4**) (Fig. 1).

## Results and Discussion

### Structure elucidation

Vlasouliolide A (**1**), 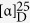 +3.77 (*c* 0.24, CH_3_COCH_3_), was obtained as a colorless orthorhombic crystal. Its molecular formula was determined to be C_32_H_40_O_5_ by positive HRESIMS at *m/z* 527.2763 ([M + Na]^+^, calcd. 527.2773). The ^1^H NMR spectrum of **1** (Supplementary Table S1) showed characteristic signals for one methyl singlet at *δ*_H_ 2.16 (3H, s, -Ac), two oxymethines at *δ*_H_ 3.91 (1H, t, *J* = 9.5 Hz, H-6′) and *δ*_H_ 4.17 (1H, t, *J* = 9.6 Hz, H-6), and four sets of terminal alkylene (*δ*_H_ 4.83, 1H, s, 4.76, 1H, s, H_2_-14; *δ*_H_ 4.88, 1H, s, 4.78, 1H, s, H_2_-14′; *δ*_H_ 5.29, 1H, d, *J* = 1.8 Hz, 5.06, 1H, d, *J* = 1.7 Hz, H_2_-15; *δ*_H_ 5.14, 1H, d, *J* = 1.9 Hz, 5.03, 1H, d, *J* = 1.7 Hz, H_2_-15′).

Analysis of the ^13^C NMR spectrum revealed the existence of 32 carbons (Supplementary Table S2), including 1 × CH_3_, 14 × CH_2_ (containing 4 sp^2^ alkylenes at *δ*_C_ 109.3, 109.6, 111.6, and 112.0), 9 × CH (containing 2 oxymethines at *δ*_C_ 82.4 and 84.5), and 8 × C (containing 3 carbonyl carbons at *δ*_C_ 177.5, 178.3 and 205.2). Detailed analysis of the NMR spectrum of **1** indicated that vlasouliolide A (**1**) should contain an acetyl due to the resonance signals at *δ*_C_ 205.2, 30.7, and *δ*_H_ 2.16 (3H, s, -Ac). Deducting the carbon resonances for the acetyl, the remaining 30 carbon resonances implied that two similar sesquiterpene lactone units existed.

**Figure 1:**
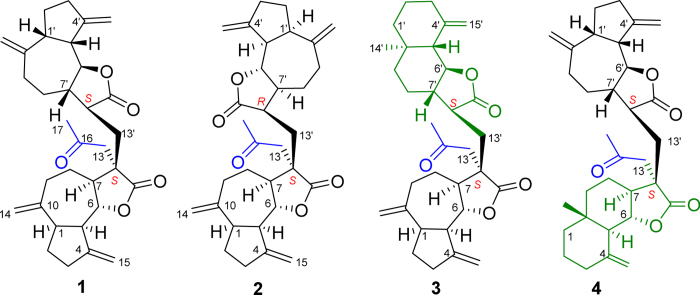
Chemical structures of **1**–**4**.

**Figure 2:**
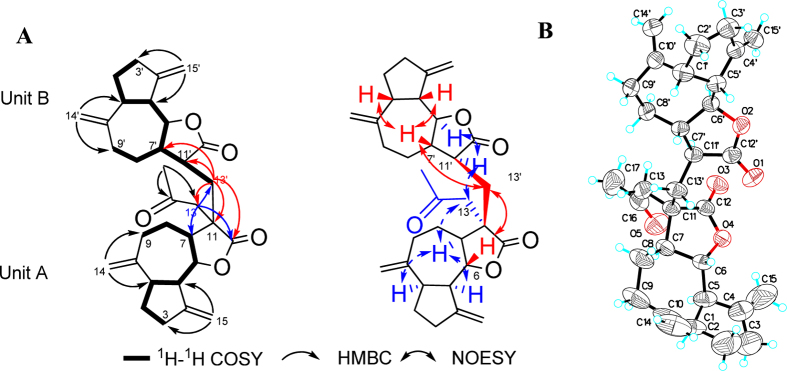
(**A**) Selected NMR correlations and (**B**) X-ray crystallographic structure of **1**.

The planar structure of vlasouliolide A was constructed by comprehensive analyses of the 2D NMR spectra (Fig. 2A). Two similar long proton-bearing structural fragments, H_2_-3/H_2_-2/H-1/H-5/H-6/H-7/H_2_-8/H_2_-9 and H_2_-3′/H_2_-2′/H-1′/H-5′/H-6′/H-7′/H_2_-8′/H_2_-9′, as well as an extra short chain, H-7′/H-11′/H_2_-13′, were constructed based on interpretation of the ^1^H-^1^H COSY and HSQC-TOCSY spectra. The HMBC spectrum of **1** showed the key correlations of four typical terminal alkylene groups (Fig. 2A), including H_2_-14/C-1 and C-9; H_2_-15/C-3 and C-5; H_2_-14′/C-1′ and C-9′; and H_2_-15′/C-3′ and C-5′. Thus, subunits A and B were deduced to be two guaianolide moieties similar to dehydrocostus lactone^14^,^15^. Therefore, the structure of **1** should be composed of two guaianolide moieties and an acetyl moiety. In the HMBC spectrum of **1**, the long correlations from H_3_-17 (*δ*_H_ 2.16, 3H, s) to C-13 (*δ*_C_ 46.4) as well as H_2_-13 (*δ*_H_ 2.86, 2.56, 1H, d, *J* = 19 Hz, respectively) to C-7 (*δ*_C_ 48.2) and C-12 (*δ*_C_ 178.3) indicated that the acetyl moiety was connected to the C-13 position of one guaianolide unit. Compared with dehydrocostus lactone, the C-11 position of subunit A was a quaternary carbon, while the C-13′ position of subunit B was an sp^3^ methylene. These changes implied that the two sesquiterpene lactone units should be linked directly *via* a C-C bond between C-11 and C-13′, and this assumption was verified by the HMBC correlations of H_2_-13′ (*δ*_H_ 2.23, 1.35, 1H, m, respectively) with C-11 (*δ*_C_ 46.1), C-12 (*δ*_C_ 178.3), C-11′ (*δ*_C_ 41.9) and C-12′ (*δ*_C_ 177.5). Thus, the planar structure of **1** was elucidated as shown in Fig. 1.

The relative configuration of **1** was characterized by interpretation of the NOESY spectrum (Fig. 2A). The similar NOESY correlations of H-1/H-7/H-5 and H-1′/H-7′/H-5′ in subunits A and B indicated that they are on the same face. In addition, the large coupling constant between H-6/H-7 and H-6′/H-7′ (*J* = 9.6, 9.5 Hz, respectively) implied that H-6/H-7 and H-6′/H-7′ were in the *trans*-form. Consequently, in subunit A, H-7 was arbitrarily assigned as having an *α*-orientation. The correlation between H-7 and H_2_-13 suggested that the side chain C_13_/C_16_/C_17_ should be located below the molecular plane. Thus, the H_2_-13′ was assigned as having a *β*-orientation, as indicated by the NOESY correlations of H-6 with H_2_-13′. It should be noted that H_2_-13′ exhibited NOESY correlations with H-7′. Combined with the NOESY correlation between H_2_-13 and H-11′, the relative configuration of H-11′ should be the *α*-orientation. Finally, the absolute configuration of **1** was confirmed to be 1*R*, 5*R*, 6*S*, 7*S*, 11*S*, 1′*R*, 5′*R*, 6′*S*, 7′*S*, 11′*S* by Cu-Κ*α* X-ray crystallographic analysis (Fig. 2B).

**Figure 3:**
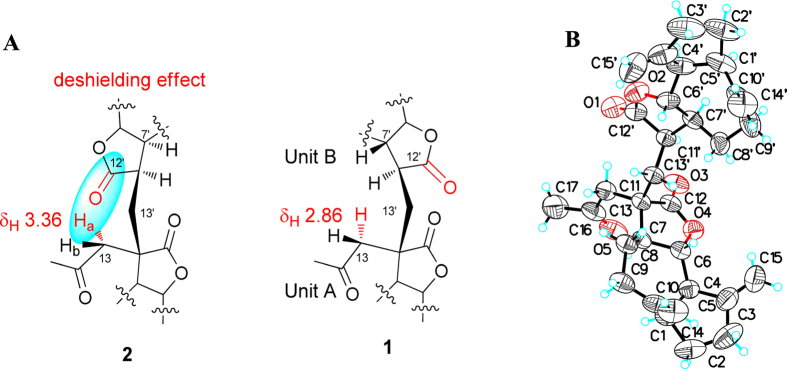
(**A**) The deshielding effect of the carbonyl group at C-12′ and (**B**) X-ray crystallographic structure of **2**.

Vlasouliolide B (**2**), 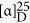 +20.41 (*c* 0.21, CH_3_COCH_3_), possessed the same molecular formula as that of **1** as determined by positive HRESIMS at *m/z* 527.2758 ([M + Na]^+^, calcd. 527.2773). Analysis of the NMR data (Supplementary Tables S1 and S2) indicated that compound **2** possesses the same planar structure as that of **1**. The notable NOESY correlation of H_2_-13/H-11′/H-7′ in **2** instead of that H-11′/H-6′ in **1** implied that **2** was an 11′-epimer of **1**. Comparing the NMR spectroscopic data of **2** with those of **1**, an obvious downfield shift of Ha-13 from *δ*_H_ 2.86 in **1** to *δ*_H_ 3.36 in the ^1^H NMR was observed. As shown in Fig. 3A, the opposite arrangement of the two subunits led to the C-12′ carbonyl and H_2_-13 in **1** being far from each other. In contrast, the subunits A and B were arranged in the same direction in **2**, resulting in the H_2_-13 being spatially adjacent to the carbon-oxygen double bond at C-12′. Thus, the intramolecular deshielding effect contributed to the downfield shift of Ha-13 from *δ*_H_ 2.86 in **1** to *δ*_H_ 3.36 in **2**. Finally, the absolute configuration of C-11′ was confirmed as *R* by Cu-Κ*α* X-ray diffraction (Fig. 3B).

**Figure 4:**
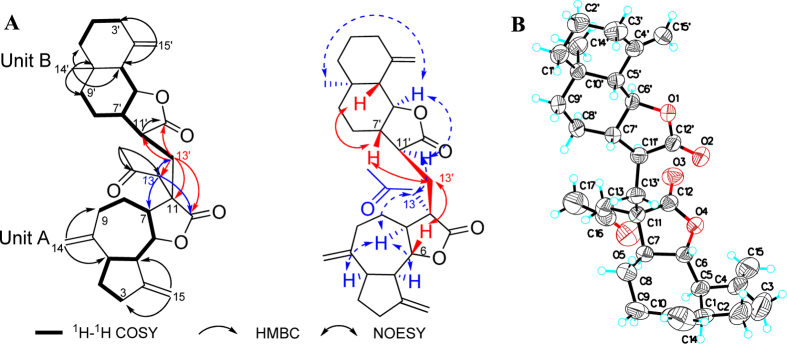
(**A**) Selected NMR correlations and (**B**) X-ray crystallographic structure of **3**.

Vlasouliolide C (**3**) was isolated as a colorless monoclinic crystal with 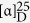 +30.23 (*c* 0.04, CHCl_3_). Its molecular formula was determined to be C_32_H_42_O_5_ by positive HRESIMS at *m/z* 529.2940 ([M + Na]^+^, calcd. 529.6728). The 1D NMR data (Supplementary Tables S1 and S2) of **3** disclosed a C_32_ skeleton similar to in **1** and **2**. Comprehensive analysis of the 2D NMR spectra indicated that compound **3** possessed the same 13-acetyl-mokkolactone fragment (subunit A) as **1** and **2**. Unlike the structures of **1** and **2**, an eudesmane moiety existed as subunit B in the structure of **3**, as deduced by the ^1^H-^1^H COSY correlations of H_2_-3′/H_2_-2′/H-1′, H-5′/H-6′/H-7′/H_2_-8′/H_2_-9′ and H-7′/H-11′/H_2_-13′ together with crucial HMBC correlations from H_2_-15′ to C-3′ and C-5′ as well as from CH_3_-14 to C-1, C-2, and C-9. Therefore, the structure of **3** was formed by an acetyl-substituted guaianolide moiety and eudesmane moiety. The HMBC correlations from H_2_-13′ (*δ*_H_ 2.30, 1.38) to C-11′ (*δ*_C_ 41.6), C-12′ (*δ*_C_ 178.8), C-11 (*δ*_C_ 46.2), C-12 (*δ*_C_ 178.3) and C-13 (*δ*_C_ 47.2) suggested that subunits A and B should also be directly linked by a C-11/13′ single bond (Fig. 4A).

The relative configuration of subunit B was deduced by analysis of the NOESY correlations (Fig. 4A). H-11′, H-6′ and H-14′ were assigned as having the *α*-orientation, and H-5′, H-7′ and H-13′ were assigned as having the *β*-orientation, which were consistent with the biosynthetic precursor *β*-cyclocostunolide^16^. Similar to **1**, the opposite arrangement of the two subunits was verified by the NOESY correlations of H-13′/H-6 and H-11′/H-13. The structure of **3** was finally elucidated as shown in Fig. 1, and the absolute configuration was assigned as 1*R*, 5*R*, 6*S*, 7*S*, 11*S*, 5′*S*, 6′*S*, 7′*S*, 10′*S*, 11′*S* by Cu-Κ*α* X-ray crystallographic analysis (Fig. 4B).

**Figure 5:**
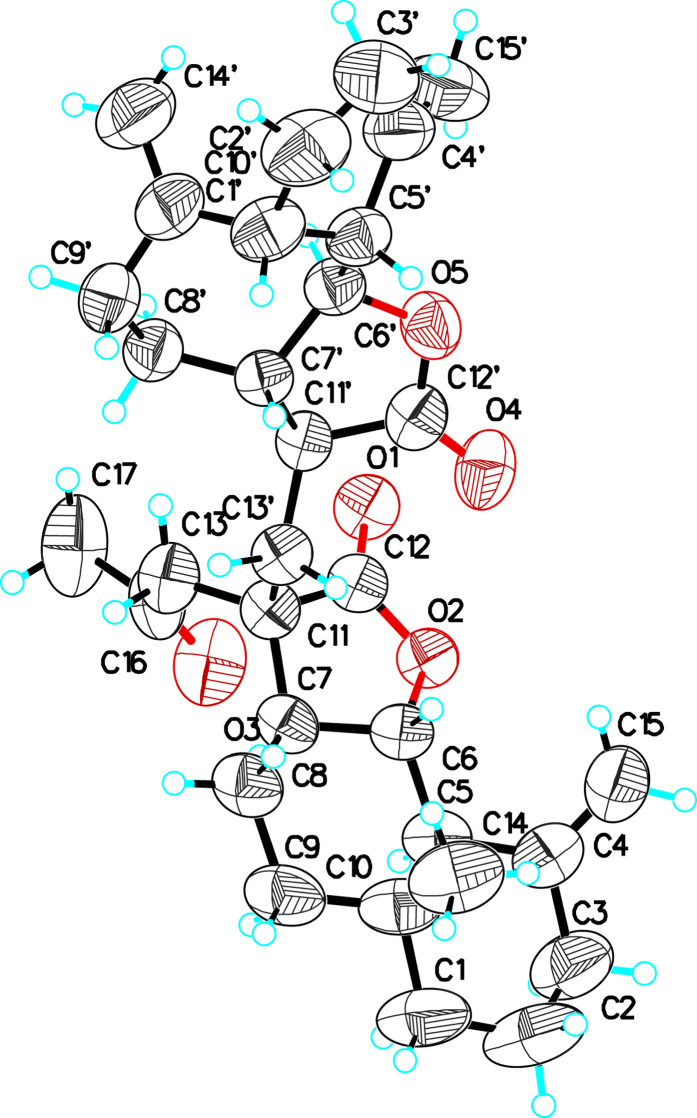
X-ray crystallographic structure of **4**.

Vlasouliolide D (**4**), a colorless orthorhombic crystal with 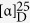 +55.21 (*c* 0.08, CH_3_COCH_3_), possessed the same molecular formula as that of **3** determined by positive HRESIMS at *m/z* 529.2923 ([M + Na]^+^ calcd. 529.6722). The 1D NMR data of **4** (Supplementary Tables S1 and S2) revealed that **4** was constructed from one acetyl, one dehydrocostus lactone, and one *β*-cyclocostunolide moieties, which were identical with those of **3**. However, different from **3**, the acetyl in **4** was located at the C-13 position of *β*-cyclocostunolide to form 13-acetyl-eudesmenolide (subunit A), which is supported by the key HMBC correlations from H_2_-13 (*δ*_H_ 2.79, 2.66, 1H, d, *J* = 17 Hz, respectively) to C-7 (*δ*_C_ 51.9), C-11 (*δ*_C_ 46.2) and C-12 (*δ*_C_ 178.9). Subunit B was also connected to subunit A *via* a C-11/13′ single bond. Therefore, the structure of **4** was determined as shown in Fig. 1. The absolute configuration of **4** was determined as 5*S*, 6*S*, 7*S*, 10*R*, 11*S*, 1′*R*, 5′*R*, 6′*S*, 7′*S*, 11′*S* by Cu-Κ*α* X-ray crystallographic analysis (Fig. 5).

### Biological activity assay

The roots of *V. souliei* are often used in traditional Chinese medicine for the treatment of digestive disorders and inflammatory diseases. Many sesquiterpenes isolated from *V. souliei* showed inhibitory activity against lipopolysaccharide (LPS)-induced nitric oxide (NO) production in murine RAW264.7 cells^10^. Excessive NO has been implicated in the pathological process of tissue damage following inflammation^17^. The inhibition of the overproduction of NO is an important therapeutic way to treat inflammatory diseases^18^. We investigated the anti-inflammatory activities of compounds **1**–**4** by a LPS-induced NO production assay in RAW 264.7 macrophages^5^. Compounds **1**–**4** exhibited significant inhibitory effects against NO production with IC_50_ values of 1.14, 2.53, 1.57 and 3.19 *μ*M, respectively. Moreover, these compounds were not notably cytotoxic at the concentrations required for inhibiting NO production, as determined by an MTT assay. NF-*κ*B is a transcription factor that controls immune responses and plays a pivotal role in the regulation of NO expression^19^. We conducted an NF-*κ*B luciferase reporter assay in 293T cells to evaluate the impact of compounds **1**–**4** on the transcriptional activity of NF-*κ*B^20^.

**Figure 6:**
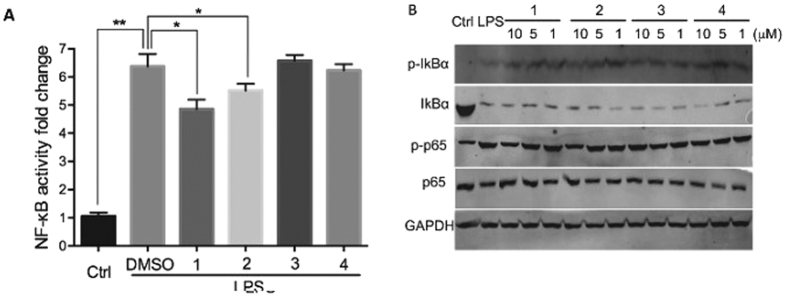
Effects of **1**–**4** on LPS-stimulated NF-*κ*B activation. (**A**) The NF-*κ*B luciferase reporter assay shows that compounds **1** and **2** displayed suppression of LPS-induced NF-*κ*B activation in 293T cells. (Mean ± SD in three separate experiments. *p < 0.05; **p < 0.001). (**B**) The effects of compounds **1**–**4** on the phosphorylation of I*κ*B*α* and p65 in the presence of LPS stimulation in RAW 264.7 cells.

The NF-*κ*B reporter luciferase construct and Renilla luciferase control vector were cotransfected in 293T cells for 24 h. Thereafter, the cells were left untreated or were treated with a 10 *μ*M concentration of compound for an additional 1 h before LPS activation for 4 h. Compounds **1** and **2** displayed inhibition towards the NF-*κ*B pathway, while **3** and **4** showed no effects in the reduction of NF-*κ*B luciferase activity (Fig. 6A). We also examined the effects of compounds **1**–**4** on the expression of I*κ*B*α* and P65 proteins in the NF-*κ*B pathway^21^. RAW 264.7 cells pretreated with **1**–**4** at the indicated concentrations for 1 h were subjected to LPS stimulation before Western blot analysis. Compounds **1**–**4** had no inhibitory effects on the degradation of I*κ*B*α*, while **1** and **2** can dose-dependently down-regulate the LPS-induced phosphorylation of the NF-*κ*B p65 subunit (Fig. 6B). These data indicated that the NO inhibitory activities of **1** and **2** might be attributed to suppressing NF-*κ*B activation, while those of **3** and **4** might not be due to this mechanism.

In conclusion, sesquiterpene lactone dimers usually have a C_30_ framework biosynthetically derived from a Diels-Alder adduct of two homo or hetero sesquiterpene monomers. Vlasouliolides A-D (**1**–**4**) possessed a C_32_ skeleton derived from two sesquiterpene lactone molecules and an acetyl group. Commonly, in the chemical structures of **1**–**4**, the acetyl was connected to the C-13 of one of the two sesquiterpene lactone units to form a C_17_ unit, and the C_17_/C_15_ units were further directly linked by a C11-C13′ single bond. This is the first report regarding C_17_/C_15_ sesquiterpene lactone dimers from nature with significant anti-inflammation activities, which might be potentially useful for the treatment of inflammatory diseases. The discovery of Vlasouliolides A-D may encourage further investigations by natural product chemists, synthetic chemists, and pharmacists.

## Methods

### General experimental procedures

Column chromatography (CC): silica gel H (10–40 *μ*m) and silica gel (200–300 mesh) (Marine Chemical Factory, Qingdao, P. R. China); Sephadex LH-20 (Pharmacia Fine Chemicals, Piscataway, NJ, USA); RP-C18 gel (40−63 *μ*m; Daiso, Co., Japan). TLC: silica gel plates (Yantai Jiang You Silicone Development Co., Yantai, P. R. China), visualization by spraying with 10% H_2_SO_4_ in EtOH. HPLC: Agilent 1260 series (Agilent Technologies, US) with a Zorbax SB-C18 (5 *μ*m, 9.4 × 150 mm) column. NMR: Bruker Avance III-500 and Avance III-600 spectrometers (Bruker, Switzerland). MS: Agilent MSD-Trap-XCT (for ESI) and Agilent-6520 Q-TOF mass spectrometers (for HR-ESI). Melting point: X-4B digital display micro-melting apparatus (Shanghai Jingsong Instrument, Shanghai, P. R. China). Optical rotation: Rudolph Autopo V (Rudolph Research Analytical, Hackettstown, NJ). UV: Agilent 1260 series DAD detector (Agilent Technologies, US). CD: Brighttime Chirascan (Applied Photophysics Ltd, UK). IR: Thermo Scientific Nicolet 6700 (Thermo Scientific, USA). RAW 264.7 cells and 293T cells: ATCC (American type culture collection). Dulbecco’s modified Eagle’s medium and fetal bovine serum: Gibco Invitrogen (Carlsbad, CA, USA). LPS Griess reagent and MTT: Sigma-Aldrich (St. Louis, MO, USA).

### Plant material

The roots of *V. souliei* were collected from the Sichuan province of China in October 2014 and authenticated by professor Bao-Kang Huang, Department of Pharmacognosy, School of Pharmacy, Second Military Medical University. A voucher specimen (No. 201412-VS) is deposited in the Department of Pharmacognosy, Second Military Medical University.

### Extraction and isolation

The chipped and dried roots of *V. souliei* (20.0 kg) were extracted by maceration with 95% ethanol overnight at room temperature (3 × 60 L). After removal of the solvent, the ethanol extract (2.12 kg) was successively partitioned between water and petroleum ether (PE)/ethyl acetate (EtOAc) to give PE, EtOAc and water extracts. The EtOAc extract (0.626 kg) was segmented by silica gel column chromatography (PE/EtOAc, 50:1–0:1) to yield 17 fractions (Fr. 1–17). Fraction 7 (31.99 g) underwent chromatography over an RP-C18 medium-pressure column (MeOH/H_2_O, 10:90 to 90:10) to give 12 subfractions (Fr. 7.1–7.12). Subfraction 7.9 (2.5 g) underwent further chromatography over an RP-C18 medium-pressure column (CH_3_CN/H_2_O, 45:55) and was finally purified by semi-preparative RP-C18 HPLC (MeOH/H_2_O, 71:29) to produce **1** (42.9 mg) and **4** (5.2 mg). Subfraction 7.10 (1.89 g) underwent chromatography over an RP-C18 medium-pressure column using MeOH/H_2_O (55:45–100:0) as the elution solvent, and the 70–80% fraction was purified by semi-preparative RP-C18 HPLC (CH_3_CN/H_2_O, 45:55), yielding **2** (22.2 mg). Fraction 8 (5.40 g) underwent chromatography over an RP-C18 medium-pressure column using MeOH/H_2_O in a gradient (10:90–100:0) to yield eight subfractions (Fr. 8.1–8.8). Subfraction 8.5 (2.35 g) was purified by an RP-C18 medium-pressure column (MeOH/H_2_O, 55:45 to 100:0) followed by semi-preparative RP-C18 HPLC (CH_3_CN/H_2_O, 63:27) to produce **3** (10.6 mg). Overall, we obtained compounds **1** (42.9 mg), **2** (22.2 mg), **3** (10.6 mg) and **4** (5.2 mg).

### Spectroscopic data

**Vlasouliolide A** (**1**) Colorless orthorhombic crystals in EtOH/H_2_O. m.p.: 169–173 °C; 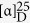 +3.77 (*c* 0.24, CH_3_COCH_3_); UV (CH_3_OH/H_2_O) λ_max_ 210 nm; IR (KBr) *ν*_max_ 3089, 2931, 2854, 1770, 1714, 1639, 1446, 1400, 1367, 1334, 1209, 1178, 1126, 995, 890 cm^−1^; ^1^H- and ^13^C-NMR data, see Tables S1 and S2; ESIMS *m/z* 527.4 ([M + Na]^+^), 503.2 ([M − H]^−^); positive HRESIMS *m/z* 527.2763 ([M + Na]^+^, calcd. 527.2773).

**Vlasouliolide B** (**2**) Colorless orthorhombic crystals in EtOH/H_2_O. m.p.: 171–173 °C; 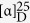 +20.41 (*c* 0.21, CH_3_COCH_3_); UV (CH_3_CN/H_2_O) λ_max_ 210, 254, 290 nm; IR (KBr) *ν*_max_ 3081, 2931, 2854, 1760, 1712, 1641, 1455, 1365, 1313, 1213, 1164, 995, 885 cm^−1^; ^1^H- and ^13^C-NMR data, see Tables S1 and S2; ESIMS *m/z* 527.3 ([M + Na]^+^), 539.3 ([M + Cl]^−^); positive HRESIMS *m/z* 527.2758 ([M + Na]^+^, calcd. 527.2773).

**Vlasouliolide C** (**3**) Colorless monoclinic crystals in CHCl_3_/MeOH. m.p.: 211–218 °C; 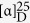 +30.23 (*c* 0.04 CHCl_3_); UV (CH_3_CN/H_2_O) λ_max_ 210 nm; IR (KBr) *ν*_max_ 3086, 2929, 2849, 1763, 1719, 1649, 1444, 1380, 1336, 1260, 1195, 1159, 1103, 1019, 991, 911, 891 cm^−1^; ^1^H- and ^13^C-NMR data, see Tables S1 and S2; ESIMS *m/z* 529.4 ([M + Na]^+^), 541.5 ([M + Cl]^−^); positive HRESIMS *m/z* 529.2940 ([M + Na]^+^, calcd. 529.2930).

**Vlasouliolide D** (**4**) Colorless orthorhombic crystals in EtOH/H_2_O. m.p.: 172–182 °C; 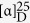 +55.21 (*c* 0.08 CH_3_COCH_3_); UV (CH_3_CN/H_2_O) λ_max_ 210 nm; IR (KBr) *ν*_max_ 2933,1772,1712, 1648, 1457, 1367, 1205, 1176, 1130, 995, 892 cm^−1^; ^1^H- and ^13^C-NMR data, see Tables S1 and S2; ESIMS *m/z* 529.4 ([M + Na]^+^), 505.3 ([M − H]^−^); positive HRESIMS *m/z* 529.2923 ([M + Na]^+^, calcd. 529.2930).

### Measurement of LPS-Induced NO Production

RAW 264.7 cells were seeded in 96-well culture plates at 5 × 10^5^ cells/well at 37 °C for 6 h in DMEM medium. The cells were pretreated with different concentrations of samples for 12 h and then incubated for 16 h with or without 1 *μ*g/mL LPS. The nitrite concentration in the culture supernatant was measured using Griess reagent (1% sulfanilamide, 0.1% N-1-naphthylenediamine dihydrochloride and 2.5% phosphoric acid). The absorbance was measured at 540 nm using a microplate reader after incubation for 15 min. The nitrite levels in the samples were calculated from a standard curve created using known concentrations of sodium nitrite.

### MTT assay

RAW 264.7 cells were seeded in 96-well plates at 5 × 10^5^ cells/well for 6 h and treated with different concentrations of compounds for 24 h. Thereafter, MTT [3-(4,5-dimethylthiazol-2-yl)-2,5-diphenyltetrazolium bromide] was added to each well at a final concentration of 0.5 mg/ml and incubated at 37 °C for 3 h. The amount of MTT formazan was determined by dissolving it in dimethyl sulfoxide (DMSO) and measuring its absorbance at 495 nm using a microplate reader.

### Dual-Luciferase reporter gene assay

293T cells were seeded in 24-well plates at 2 × 10^6^ cells/well for 6 h. After being cotransfected with expression plasmids for NF-*κ*B firefly luciferase and TK-Renilla luciferase for 24 h, the cells were treated with 10 *μ*M concentrations of compounds **1**–**4** for an additional 1 h. Thereafter, the cells were stimulated with 1 *μ*g/ml LPS for another 6 h and then lysed in lysis buffer. Luciferase activities were measured by the dual-luciferase reporter gene assay system (Promega). NF-*κ*B firefly luciferase activity was normalized to the *Renilla* luciferase activity.

### Antibodies and Western blot analysis

Antibodies for p-p65, p65, p-I*κ*B*α*, I*κ*B*α* and Gapdh were purchased from Cell Signaling Technology. For western blot analysis, RAW 264.7 cells were lysed in 2× SDS sample buffer (62.5 mM Tris-HCl, pH = 6.8, 2% SDS, 10% glycerol, 50 mM DTT, and 0.01% bromophenol blue) after treatment with the compounds and stimulation by LPS. Cell lysates were fractionated by sodium dodecyl sulfate–polyacrylamide gel electrophoresis (SDS-PAGE) after denaturation treatment and subjected to immunoblot analysis with GAPDH as the loading control.

## Additional Information

**How to cite this article**: Chen, L.-P. *et al*. Vlasouliolides A-D, four rare C_17_/C_15_ sesquiterpene lactone dimers with potential anti-inflammatory activity from *Vladimiria souliei. Sci. Rep.* **7**, 43837; doi: 10.1038/srep43837 (2017).

**Publisher's note:** Springer Nature remains neutral with regard to jurisdictional claims in published maps and institutional affiliations.

## Supplementary Material

Supporting Information

Supplementary Dataset 1

Supplementary Dataset 2

Supplementary Dataset 3
