# Supplementary material for: Vlasouliolides A-D, four rare C17/C15 sesquiterpene lactone dimers with potential anti-inflammatory activity from Vladimiria souliei
Source: Sci Rep. 2017 Mar 3;7:43837. doi: 10.1038/srep43837 (PMC5335558; doi:10.1038/srep43837)
Supplement: Supporting Information [file srep43837-s1.pdf]

Supporting Information

Vlasouliolides A-D, four rare C_{17}/C_{15} sesquiterpene lactone dimers with potential anti-inflammatory activity from *Vladimiria souliei*

Li-Ping Chen[1,+], Guo-Zhen Wu[1,+], Jian-Ping Zhang[1], Ji Ye[1], Qing-Xin Liu[1], Yun-Heng Shen[1], Hui-Liang Li[1,*] and Wei-Dong Zhang[1,2,*]

[1]Department of Phytochemistry, School of Pharmacy, Second Military Medical University, Shanghai 200433, P. R. China

[2]Shanghai Institute of Pharmaceutical Industry, Shanghai 200040, P. R. China

[*]faranli@hotmail.com & wdzhangy@hotmail.com.

[+]These authors contributed equally to this work.

Contents

Table S1. ^{1}H NMR (600 MHz of **1** and **2**, 500 MHz of **3** and **4**, $CDCl_3$, *J* in Hz, in Me_4Si) Spectroscopic Data for **1−4**........4
Table S2. ^{13}C NMR (150 MHz of **1** and **2**, 125 MHz of **3** and **4**, $CDCl_3$, in Me_4Si) Spectroscopic Data for **1−4**........5
Figure S1. HRESIMS spectrum of Vlasouliolide A (**1**)........6
Figure S2. IR spectrum of Vlasouliolide A (**1**)........7
Figure S3. OR Value of Vlasouliolide A (**1**) in CH_3COCH_3........8
Figure S4. CD spectrum of Vlasouliolide A (**1**) in CH_3CN........9
Figure S5. UV spectrum of Vlasouliolide A (**1**) in CH_3OH/H_2O........10
Figure S6. ^{1}H NMR spectrum of Vlasouliolide A (**1**) in $CDCl_3$........11
Figure S7. ^{13}C NMR spectrum of Vlasouliolide A (**1**) in $CDCl_3$........12
Figure S8. DEPT spectrum of Vlasouliolide A (**1**) in $CDCl_3$........13
Figure S9. ^{1}H-^{1}H COSY spectrum of Vlasouliolide A (**1**) in $CDCl_3$........14
Figure S10. HSQC spectrum of Vlasouliolide A (**1**) in $CDCl_3$........15
Figure S11. HMBC spectrum of Vlasouliolide A (**1**) in $CDCl_3$........16
Figure S12. NOESY spectrum of Vlasouliolide A (**1**) in $CDCl_3$........17
Figure S13. HSQC-TOCSY spectrum of Vlasouliolide A (**1**) in $CDCl_3$........18
Figure S14. HSQC-HMBC spectrum of Vlasouliolide A (**1**) in $CDCl_3$........19
Figure S15. X-ray structure of Vlasouliolide A (**1**)........20
Table S3. Crystal data and structure refinement for Vlasouliolide A (**1**)........21
Figure S16. HRESIMS spectrum of Vlasouliolide B (**2**)........22
Figure S17. IR spectrum of Vlasouliolide B (**2**)........23
Figure S18. OR value of Vlasouliolide B (**2**) in CH_3COCH_3........24
Figure S19. CD spectrum of Vlasouliolide B (**2**) in CH_3CN........25
Figure S20. UV spectrum of Vlasouliolide B (**2**) in CH_3CN/H_2O........26
Figure S21. ^{1}H NMR spectrum of Vlasouliolide B (**2**) in $CDCl_3$........27
Figure S22. ^{13}C NMR spectra of Vlasouliolide B (**2**) in $CDCl_3$........28
Figure S23. DEPT spectra of Vlasouliolide B (**2**) in $CDCl_3$........29
Figure S24. ^{1}H-^{1}H COSY spectrum of Vlasouliolide B (**2**) in $CDCl_3$........30
Figure S25. HSQC spectrum of Vlasouliolide B (**2**) in $CDCl_3$........31
Figure S26. HMBC spectrum of Vlasouliolide B (**2**) in $CDCl_3$........32
Figure S27. NOESY spectrum of Vlasouliolide B (**2**) in $CDCl_3$........33
Figure S28. HSQC-TOCSY spectrum of Vlasouliolide B (**2**) in $CDCl_3$........34
Figure S29. HSQC-HMBC spectrum of Vlasouliolide B (**2**) in CDCl........35
Figure S30. X-ray structure of Vlasouliolide B (**2**)........36
Table S4. Crystal data and structure refinement for Vlasouliolide B (**2**)........37
Figure S31. HRESIMS spectrum of Vlasouliolide C (**3**)........38
Figure S32. IR spectrum of Vlasouliolide C (**3**)........39
Figure S33. OR Value of Vlasouliolide C (**3**) in $CHCl_3$........40
Figure S34. CD spectrum of Vlasouliolide C (**3**) in CH_3COCH_3........41
Figure S35. UV spectrum of Vlasouliolide C (**3**) in CH_3CN/H_2O........42
Figure S36. ^{1}H NMR spectrum of Vlasouliolide C (**3**) in $CDCl_3$........43
Figure S37. ^{13}C NMR spectra of Vlasouliolide C (**3**) in $CDCl_3$........44
Figure S38. DEPT spectra of Vlasouliolide C (**3**) in $CDCl_3$........45
Figure S39. ^{1}H-^{1}H COSY spectrum of Vlasouliolide C (**3**) in $CDCl_3$........46
Figure S40. HSQC spectrum of Vlasouliolide C (**3**) in $CDCl_3$........47
Figure S41. HMBC spectrum of Vlasouliolide C (**3**) in $CDCl_3$........48
Figure S42. NOESY spectrum of Vlasouliolide C (**3**) in $CDCl_3$........49
Figure S43. X-ray structure of Vlasouliolide C (**3**)........50
Table S5. Crystal data and structure refinement for Vlasouliolide C (**3**)........51
Figure S44. HRESIMS spectrum of Vlasouliolide D (**4**)........52
Figure S45. IR spectrum of Vlasouliolide D (**4**)........53

Figure S46. OR Value of Vlasouliolide D (**4**) in CH_3COCH_3....54
Figure S47. CD spectrum of Vlasouliolide D (**4**) in CH_3CN....55
Figure S48. UV spectrum of Vlasouliolide D (**4**) in CH_3CN/H_2O....56
Figure S49. ^{1}H NMR spectrum of Vlasouliolide D (**4**) in $CDCl_3$....57
Figure S50. ^{13}C NMR spectra of Vlasouliolide D (**4**) in $CDCl_3$....58
Figure S51. DEPT spectra of Vlasouliolide D (**4**) in $CDCl_3$....59
Figure S52. ^{1}H-^{1}H COSY spectrum of Vlasouliolide D (**4**) in $CDCl_3$....60
Figure S53. HSQC spectrum of Vlasouliolide D (**4**) in $CDCl_3$....61
Figure S54. HMBC spectrum of Vlasouliolide D (**4**) in $CDCl_3$....62
Figure S55. NOESY spectrum of Vlasouliolide D (**4**) in $CDCl_3$....63
Figure S56. HSQC-TOCSY spectrum of Vlasouliolide D (**4**) in $CDCl_3$....64
Figure S57. X-ray structure of Vlasouliolide D (**4**)....65
Table S6. Crystal data and structure refinement for Vlasouliolide D (**4**)....66
Biological activity assay....67
Table S7. IC_{50} values (μM) of **1-4** from *Vladimiria souliei* against LPS-induced nitric oxide (NO) production....67
Table S8. Cytotoxicity Assay of **1-4** from *Vladimiria souliei* (Mean±SD, n=3)....67

Table S1. ^{1}H NMR (600 MHz of **1** and **2**, 500 MHz of **3** and **4**, $CDCl_3$, *J* in Hz, in Me_4Si) Spectroscopic Data for **1**−**4**

NO	**1**[ab]	**2**[ab]	**3**[ab]	**4**[ab]
1	2.85[c]	2.86[c]	2.87[c]	1.40[c]
2	1.95, m	1.94[c]	1.88[c], 1.93[c]	2.07[c]
3	2.46[c]	2.53, m	2.52, m	2.30, m 2.00, m,
5	2.97, t (8.7)	3.0, t (8.9)	2.99, t (8.8)	2.18[c]
6	4.17, t (9.5)	3.98, t (9.4)	4.16, t (9.5)	4.45, t (10.8)
7	2.66, m	2.67, m	2.65, t (2.8)	2.33, m
8	1.81[c], 1.43, m	1.78[c], 1.25, m,	1.81[c], 1.46[c]	1.68[c]
9	2.48[c], 1.93, m,	2.48[c], 1.94[c]	2.50, m, 1.94[c]	1.55[c], 1.33[c]
13	2.86[c], 2.56, d (18.0)	3.36, d (19.2),<br>2.69, d (19.3)	2.87[c], 2.58, d (18.2)	2.81, d (17.7),<br>2.66, d (17.6)
14	4.83, s,4.76, s	4.85, s, 4.76, s	4.86, s. 4.78 s	0.83, s
15	5.29, d (1.8),<br>5.06, d (1.7)	5.22, d (1.8),<br>5.06, d (1.8)	5.24, d (1.7),<br>5.07, d (1.6)	4.88, s , 4.92, s
17	2.16, s	2.18, s	2.17, s	2.16, s
1′	2.83[c]	2.86[c]	143, m	2.84, m
2′	1.81[c]	1.82[c]	1.62[c]	1.92[c]
3′	2.52[c]	2.49[c]	2.32, m, 1.98[c]	2.50[c]
5′	2.73, t (8.8)	2.79, t (9.1)	2.07, d (10.9)	2.75, t (8.9)
6′	3.91, t (9.5)	3.91, q (6.4)	4.02, t (10.5)	3.94, t (9.3)
7′	1.98, m	2.46[c]	1.66[c]	1.99 [c]
8′	2.12, m, 1.35[c]	1.82[c]	1.88[c]	2.07[c], 1.35, m
9′	2.52[c], 2.02, m	2.46[c], 1.98[c]	1.63, m, 1.32[c]	2.50[c], 2.02[c]
11′	2.53[c]	3.13, m	2.60, m	2.55, m
13′	2.23, dd (14.7), 1.35[c]	1.88, d (3.3),<br>1.44, dd (14.9)	2.30, d (4.5),<br>1.38, d (4.1)	1.58, m,<br>2.21, dd (14.7)
14′	4.88, s, 4.78, s	4.85, s, 4.74, s	0.86, s	4.89, s, 4.78, s
15′	5.14, d (1.9),<br>5.03, d (1.7)	5.15, d (1.8),<br>5.03, d (1.5)	4.92, d (1.1),<br>4.77, d (1.1)	5.15, s, 5.02, s

[a]Figures in parentheses are coupling constants in hertz. [b]Measured in $CDCl_3$. [c]Multiplicity patterns were unclear due to signal overlapping.

Table S2. ^{13}C NMR (150 MHz of **1** and **2**, 125 MHz of **3** and **4**, $CDCl_3$, in Me_4Si) Spectroscopic Data for **1**−**4**

NO	1	2	3	4
1	46.9	46.6	46.9	41.7
2	29.9	29.9	30.0	22.8
3	32.4	32.6	32.5	35.9
4	151.2	152.1	151.3	144.2
5	52.0	52.0	52.1	54.5
6	82.4	84.1	83.9	77.1
7	48.2	47.7	47.8	51.9
8	27.5	27.5	27.5	19.5
9	37.6	38.1	37.6	39.9
10	150.1	149.9	149.9	38.2
11	46.1	45.8	46.2	46.2
12	178.3	178.4	178.3	178.9
13	46.4	45.2	47.2	46.4
14	111.6	111.6	111.9	18.0
15	109.6	108.9	109.7	109.3
16	205.2	205.9	205.1	205.7
17	30.7	30.4	30.6	31.3
1′	47.0	46.8	41.7	47.1
2′	30.0	30.0	22.8	30.1
3′	32.3	32.3	35.9	32.4
4′	151.3	151.6	144.4	151.4
5′	51.8	52.2	54.3	51.8
6′	84.5	85.8	79.2	84.8
7′	49.2	45.1	52.3	48.9
8′	32.2	28.7	23.5	32.2
9′	37.2	37.4	40.0	37.3
10′	149.5	149.9	38.3	149.3
11′	41.9	37.9	41.6	41.7
12′	177.5	179.6	178.8	178.0
13′	30.7	26.1	30.7	28.2
14′	112.0	111.7	18.0	112.1
15′	109.3	109.2	109.0	109.4

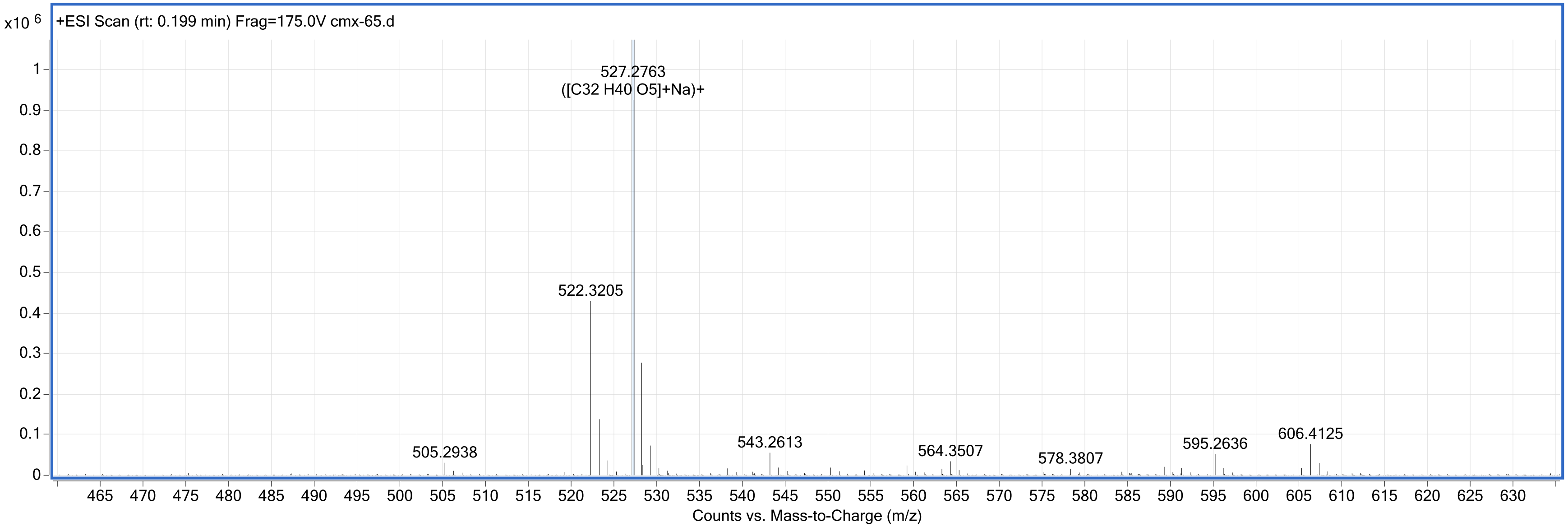

Best	Formula (M)	Ion Formula	m/z	Calc m/z	Score	Mass	Calc Mass	Diff (ppm)
TRUE	$C_{32}H_{40}O_5$	$C_{32}H_{40}NaO_5$	527.2763	527.2773	84.76	504.287	504.29	1.14

Figure S1. HRESIMS spectrum of Vlasouliolide A (**1**)

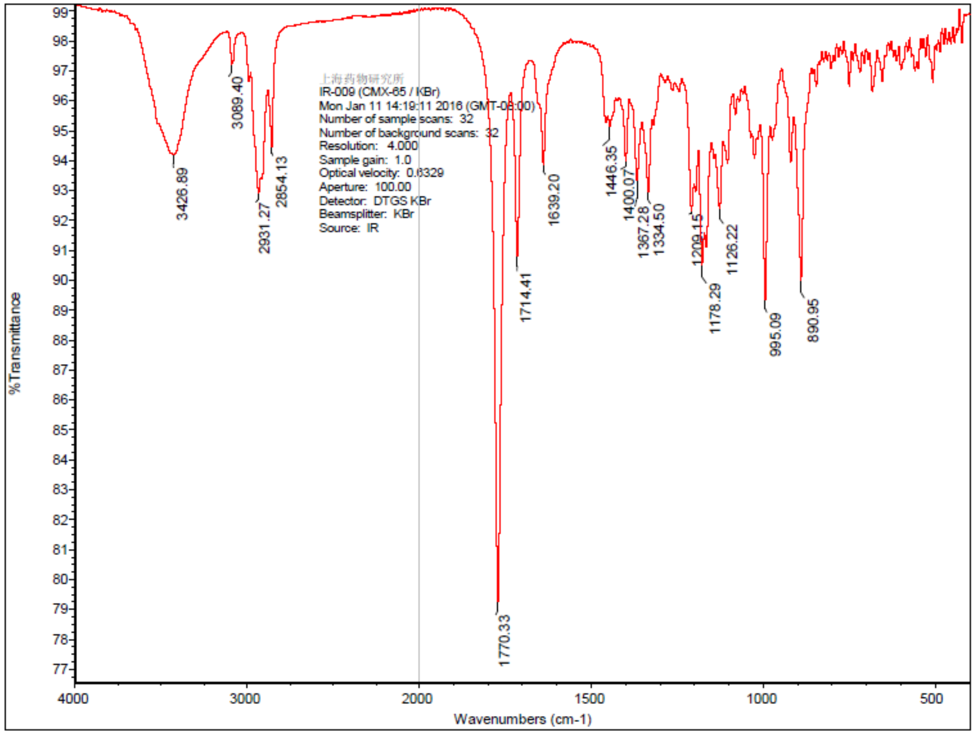

The IR absorption at 3426.89 cm^{-1} was attributed to residual solvent or impurity.

Figure S2. IR spectrum of Vlasouliolide A (**1**)

Rudolph Research Analytical

Friday, 01/22/2016

This sample was measured on an Autopol VI, serial number 90079,
manufactured by Rudolph Research Analytical,Hackettstown,NJ.

LotID : CMX-65
Set Temperature : 20.0
Temp Corr : OFF

n	Average	Std.Dev.	Maximum	Minimum
6	3.766	0.0000	3.766	3.766

S.No	Sample ID	Time	Result	Scale	OR ° Arc	WLG	Lg.mm	Conc.	Temp.	Comment
1	CMX-65	01:20:42 PM	3.766	SR	0.009	589	100.00	0.239	19.7	
2	CMX-65	01:20:49 PM	3.766	SR	0.009	589	100.00	0.239	19.7	
3	CMX-65	01:20:57 PM	3.766	SR	0.009	589	100.00	0.239	19.7	
4	CMX-65	01:21:04 PM	3.766	SR	0.009	589	100.00	0.239	19.7	
5	CMX-65	01:21:11 PM	3.766	SR	0.009	589	100.00	0.239	19.7	
6	CMX-65	01:21:18 PM	3.766	SR	0.009	589	100.00	0.239	19.7	

Signature

Figure S3. OR Value of Vlasouliolide A (**1**) in CH_3COCH_3

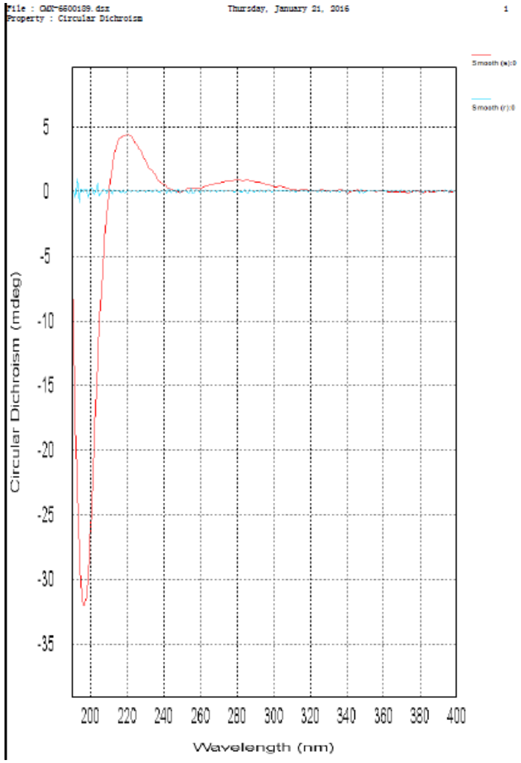

Figure S4. CD spectrum of Vlasouliolide A (**1**) in CH_3CN

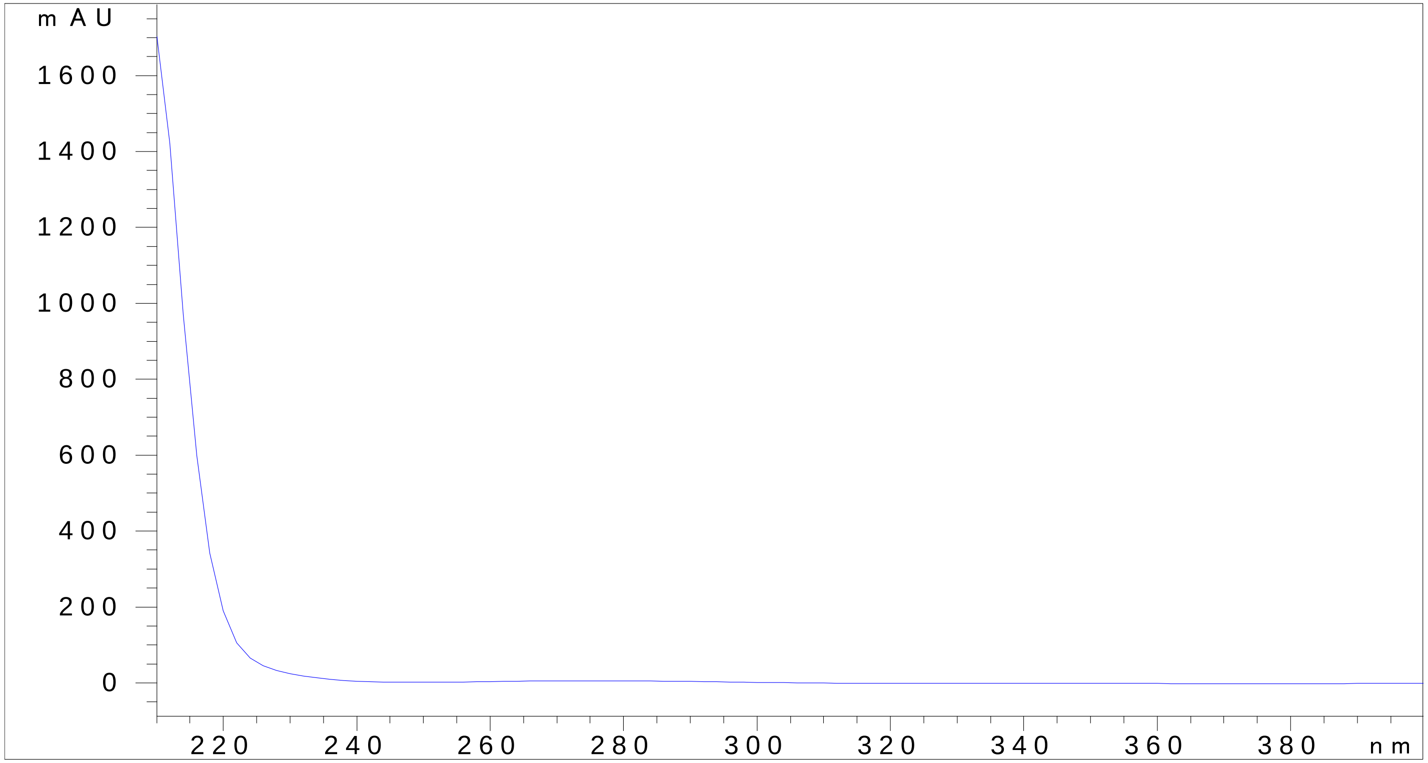

Figure S5. UV spectrum of Vlasouliolide A (**1**) in CH_3OH/H_2O

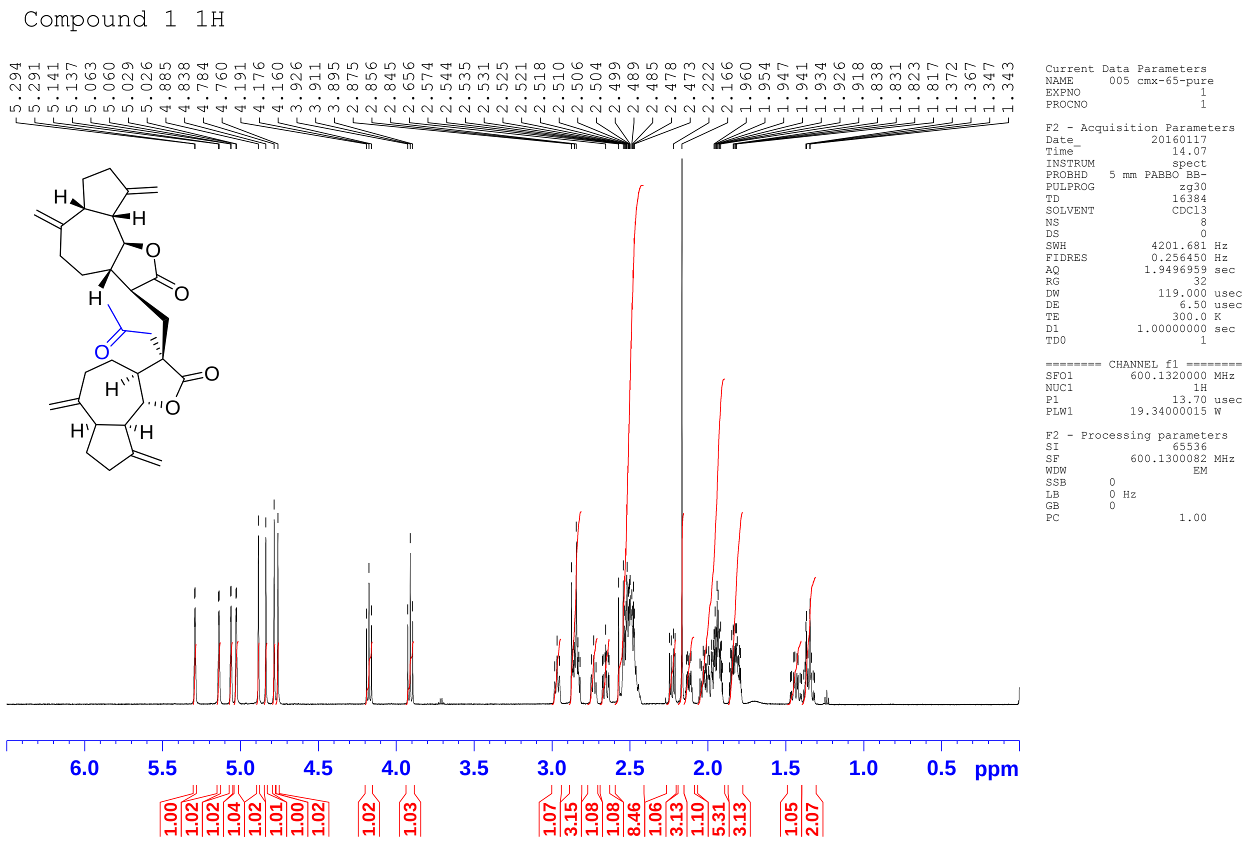

Figure S6. ^{1}H NMR spectrum of Vlasouliolide A (**1**) in $CDCl_3$

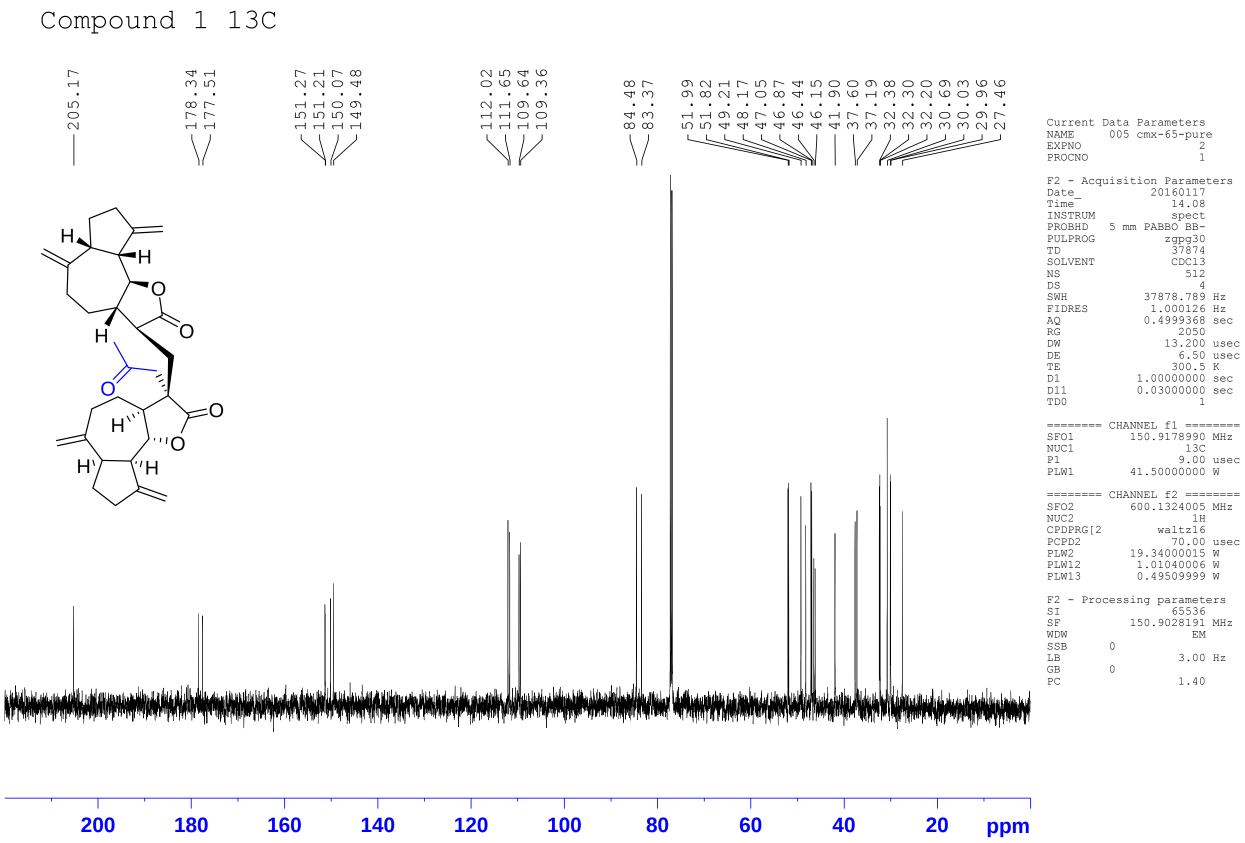

Figure S7. ^{13}C NMR spectrum of Vlasouliolide A (**1**) in $CDCl_3$

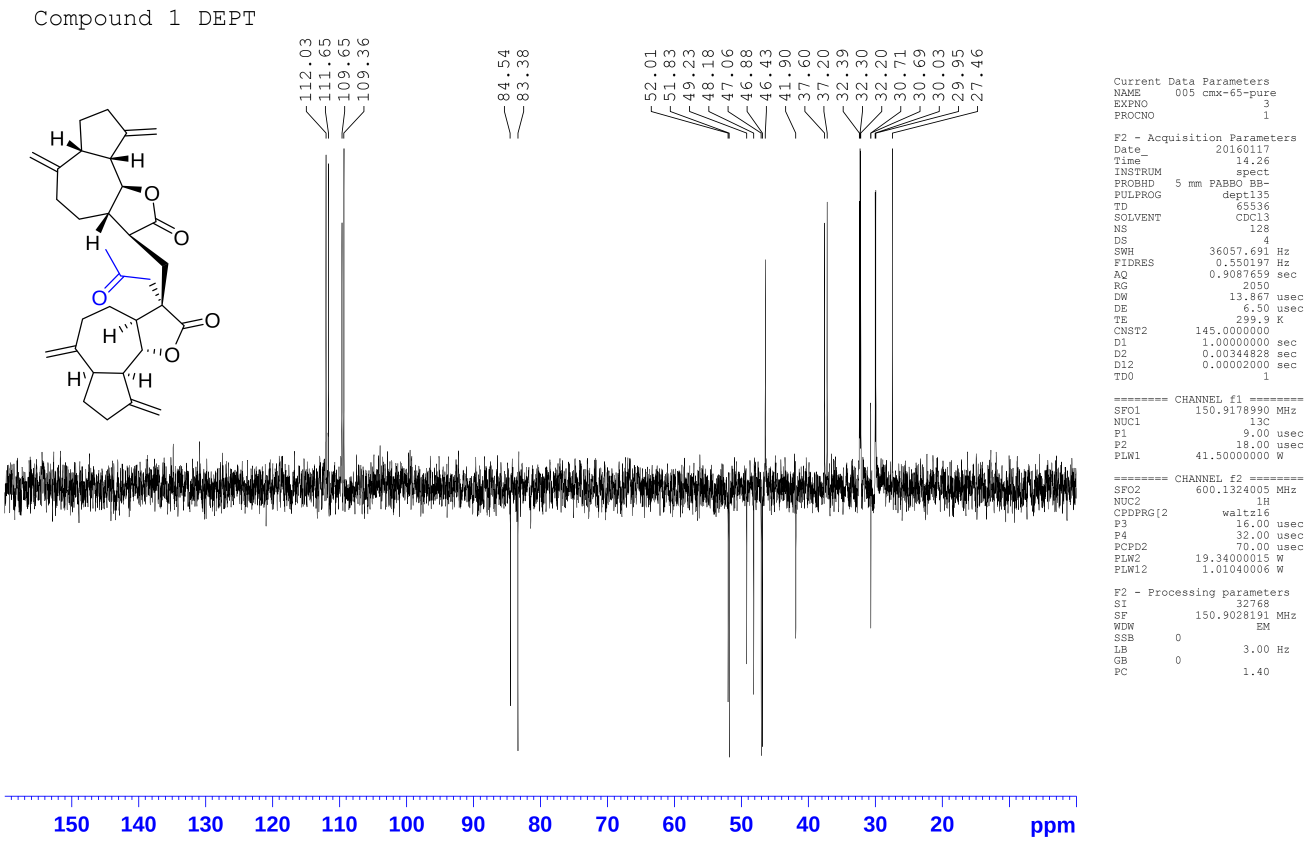

Figure S8. DEPT spectrum of Vlasouliolide A (**1**) in $CDCl_3$

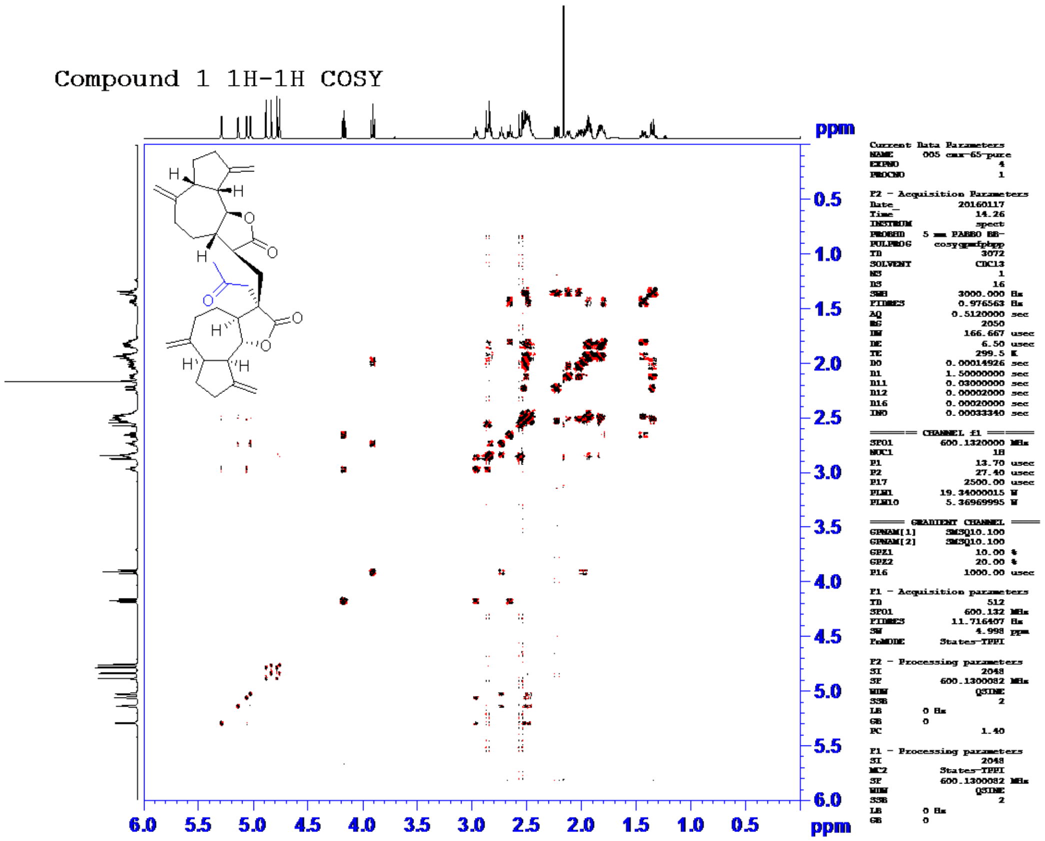

Figure S9. ^{1}H-^{1}H COSY spectrum of Vlasouliolide A (**1**) in $CDCl_3$

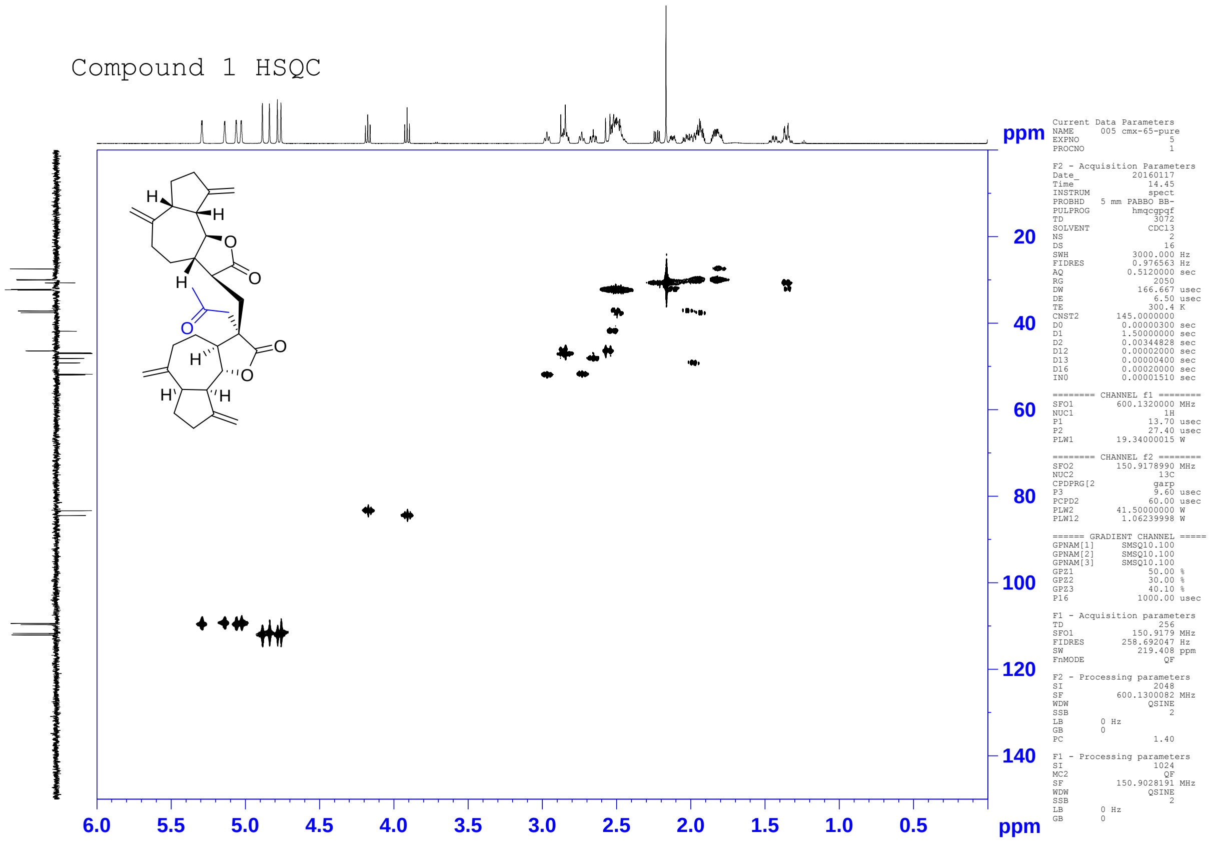

Figure S10. HSQC spectrum of Vlasouliolide A (**1**) in $CDCl_3$

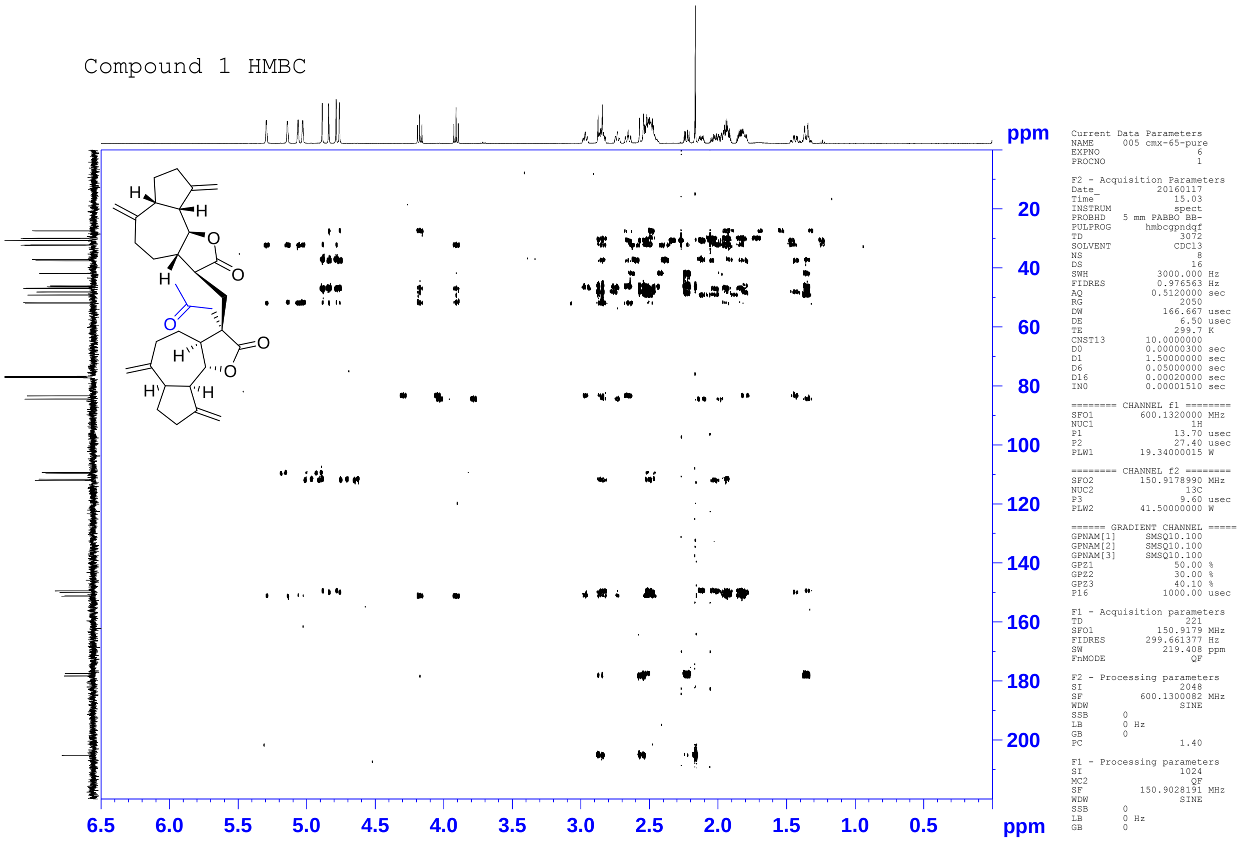

Figure S11. HMBC spectrum of Vlasouliolide A (**1**) in $CDCl_3$

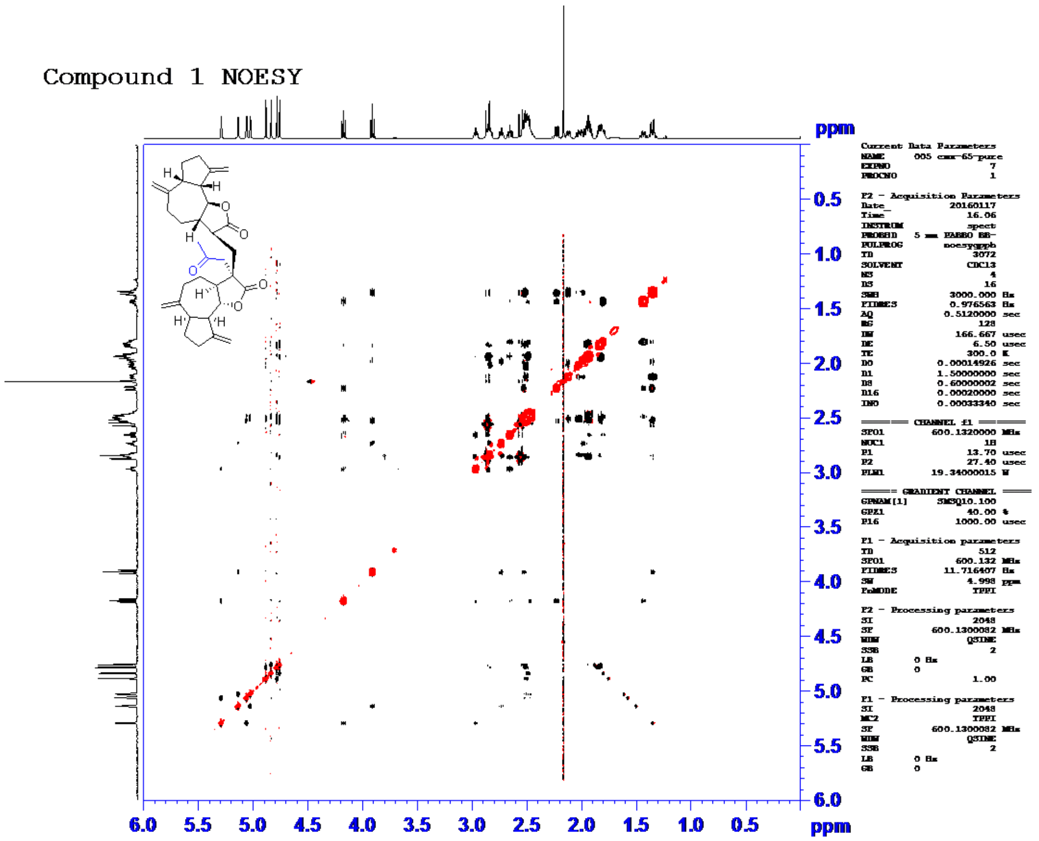

Figure S12. NOESY spectrum of Vlasouliolide A (**1**) in $CDCl_3$

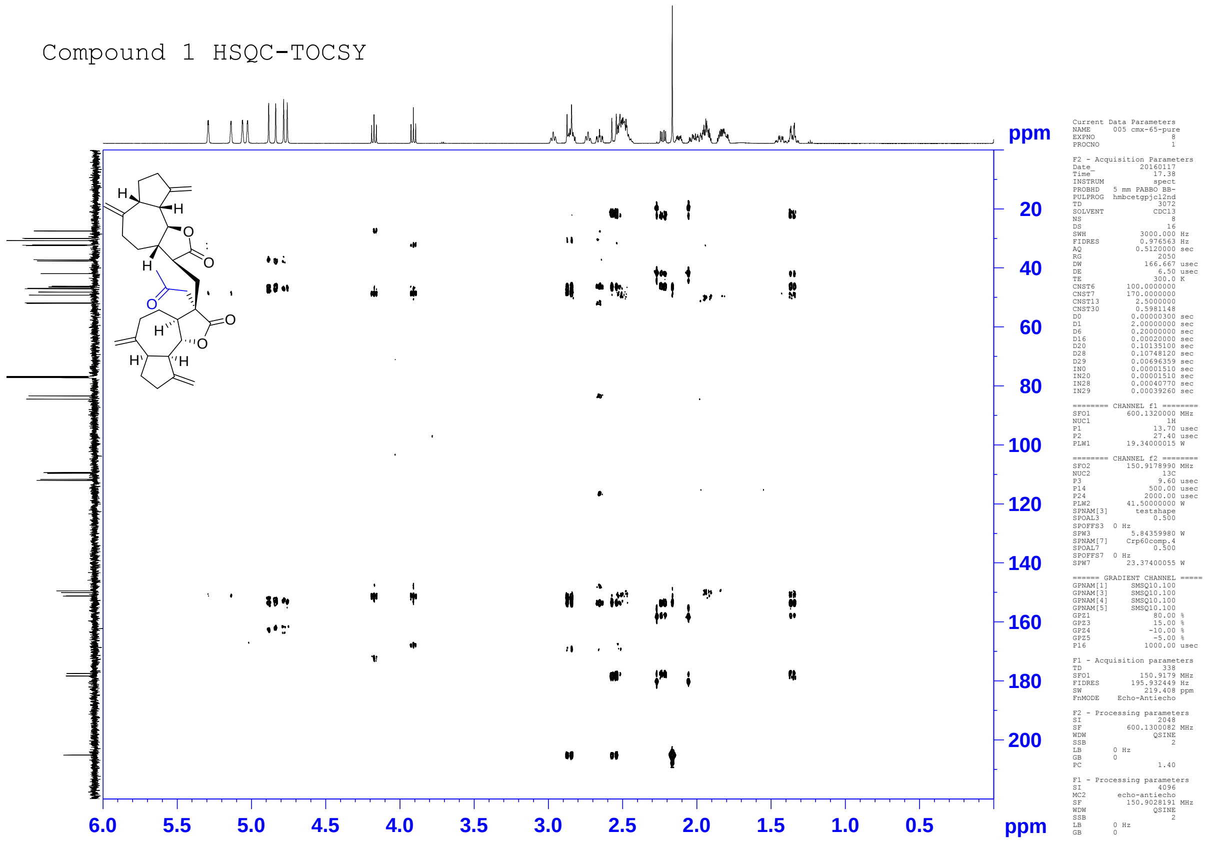

Figure S13. HSQC-TOCSY spectrum of Vlasouliolide A (**1**) in $CDCl_3$

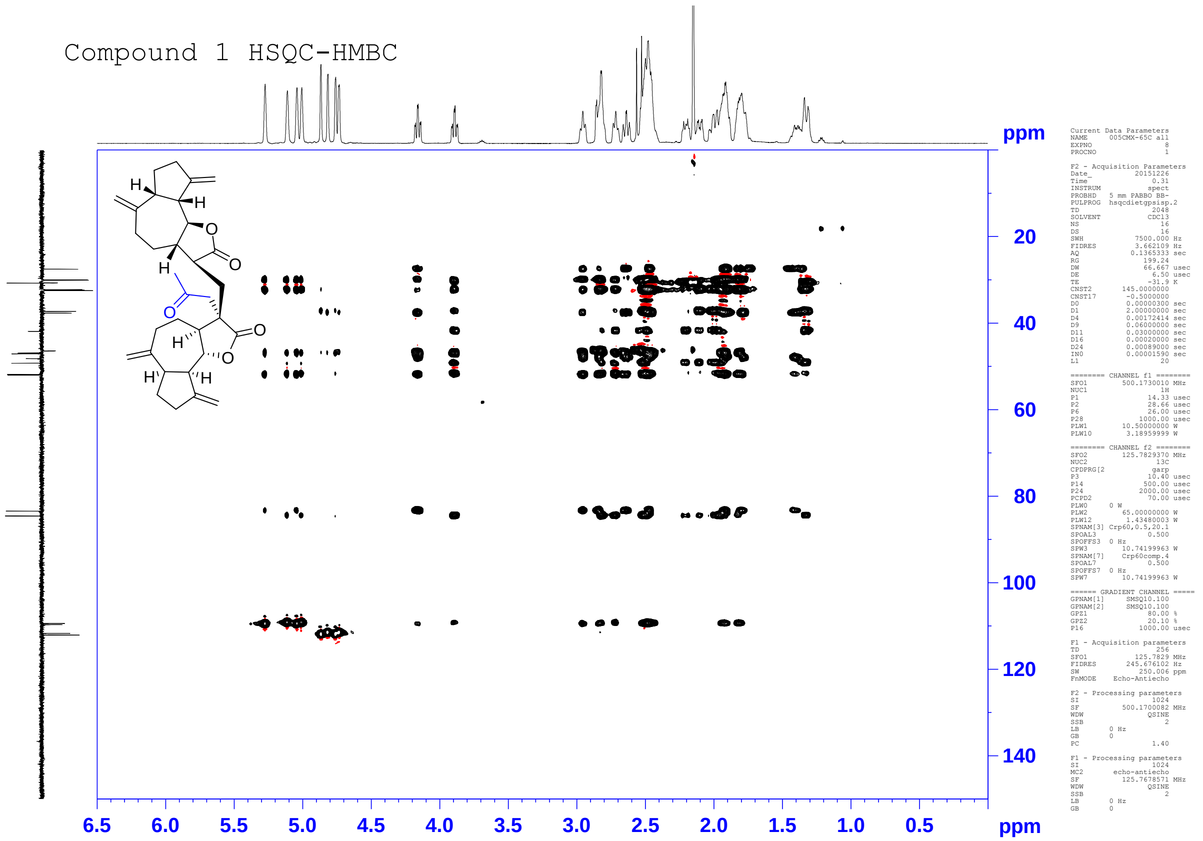

Figure S14. HSQC-HMBC spectrum of Vlasouliolide A (**1**) in $CDCl_3$

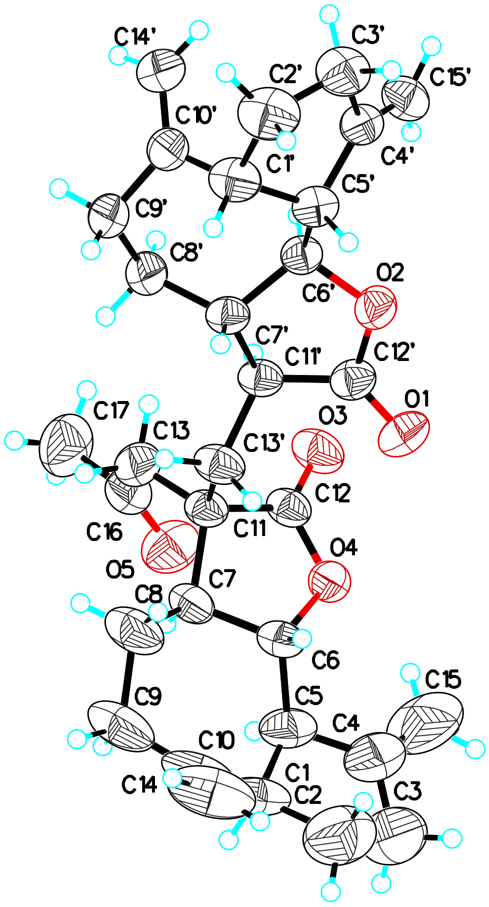

Figure S15. X-ray structure of Vlasouliolide A (**1**)

Table S3. Crystal data and structure refinement for Vlasouliolide A (**1**)

Identification code	dm15912	
Empirical formula	C32 H40 O5	
Formula weight	504.64	
Temperature	293.15 K	
Wavelength	1.54178 Å	
Crystal system	Orthorhombic	
Space group	P 21 21 2	
Unit cell dimensions	a = 11.9227(2) Å	α= 90 °.
	b = 31.4691(4) Å	β= 90 °.
	c = 7.42200(10) Å	γ = 90 °.
Volume	2784.71(7) $Å^3$	
Z	4	
Density (calculated)	1.204 Mg/m^3	
Absorption coefficient	0.635 mm^{-1}	
F(000)	1088	
Crystal size	0.28 x 0.12 x 0.05 mm^3	
Theta range for data collection	2.808 to 69.635 °.	
Index ranges	-14<=h<=14, -38<=k<=37, -6<=l<=8	
Reflections collected	17344	
Independent reflections	4929 [R(int) = 0.0473]	
Completeness to theta = 67.679 °	97.2 %	
Absorption correction	Semi-empirical from equivalents	
Max. and min. transmission	0.7532 and 0.4574	
Refinement method	Full-matrix least-squares on F^2	
Data / restraints / parameters	4929 / 90 / 335	
Goodness-of-fit on F^2	1.042	
Final R indices [I>2sigma(I)]	R1 = 0.0554, wR2 = 0.1575	
R indices (all data)	R1 = 0.0594, wR2 = 0.1643	
Absolute structure parameter	0.07(10)	
Extinction coefficient	n/a	
Largest diff. peak and hole	0.426 and -0.281 e.$Å^{-3}$	

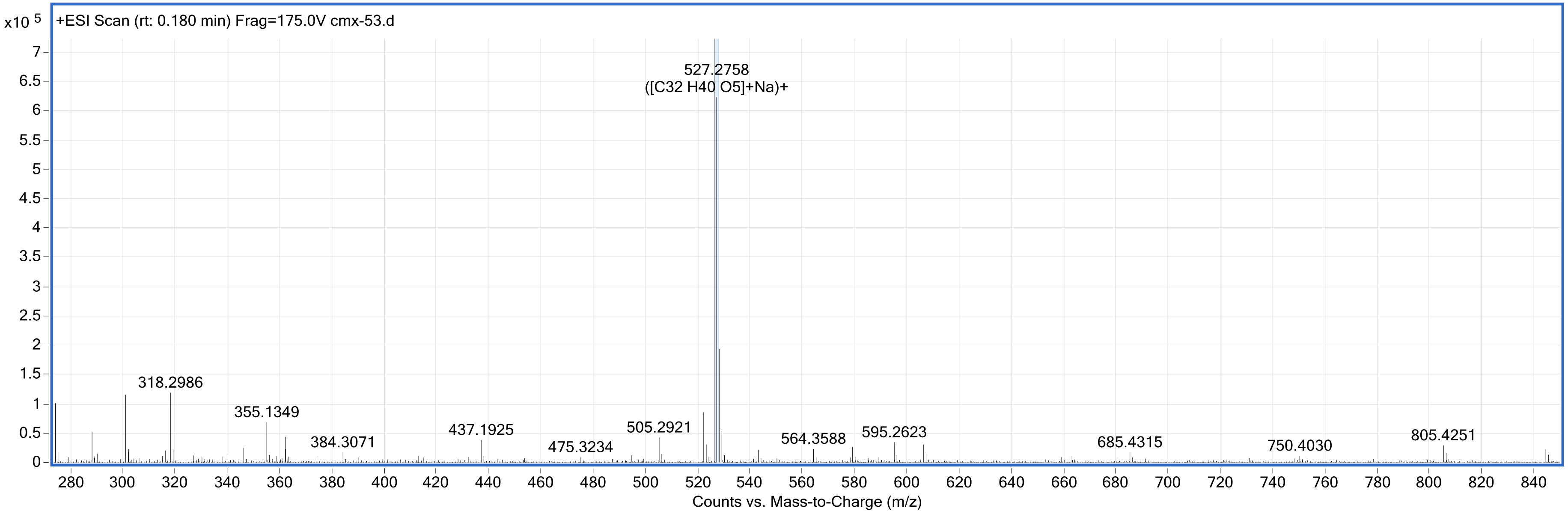

Best	Formula (M)	Ion Formula	m/z	Calc m/z	Score	Mass	Calc Mass	Diff (ppm)
TRUE	$C_{32}H_{40}O_5$	$C_{32}H_{40}NaO_5$	527.2658	527.2773	93.79	504.2867	504.29	1.83

Figure S16. HRESIMS spectrum of Vlasouliolide B (**2**)

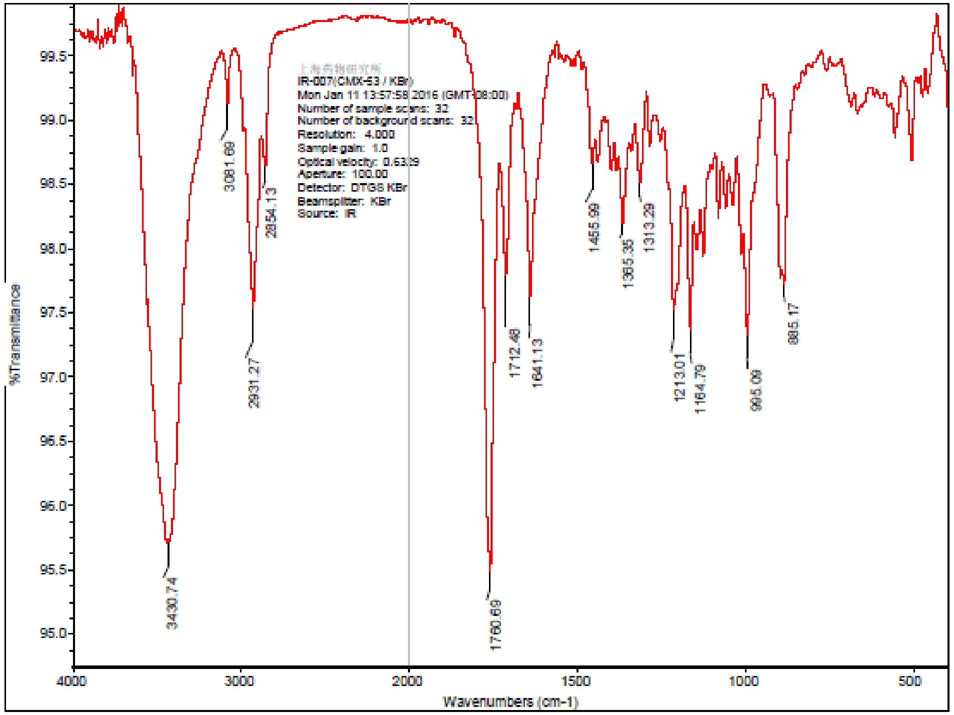

The IR absorption at 3430.74 cm^{-1} was attributed to residual solvent or impurity.

Figure S17. IR spectrum of Vlasouliolide B (**2**)

Rudolph Research Analytical

Friday, 01/22/2016

This sample was measured on an Autopol VI, serial number 90079,
manufactured by Rudolph Research Analytical,Hackettstown,NJ.

LotID : CMX-53
Set Temperature : 20.0
Temp Corr : OFF

n	Average	Std.Dev.	Maximum	Minimum
6	20.407	0.3674	20.976	20.000

S.No	Sample ID	Time	Result	Scale	OR ° Arc	WLG	Lg.mm	Conc.	Temp.	Comment
1	CMX-53	01:08:23 PM	20.488	SR	0.042	589	100.00	0.205	20.2	
2	CMX-53	01:08:30 PM	20.000	SR	0.041	589	100.00	0.205	20.2	
3	CMX-53	01:08:38 PM	20.000	SR	0.041	589	100.00	0.205	20.1	
4	CMX-53	01:08:45 PM	20.488	SR	0.042	589	100.00	0.205	20.1	
5	CMX-53	01:08:52 PM	20.488	SR	0.042	589	100.00	0.205	20.1	
6	CMX-53	01:08:59 PM	20.976	SR	0.043	589	100.00	0.205	20.1	

____

Signature

Figure S18. OR value of Vlasouliolide B (**2**) in CH_3COCH_3

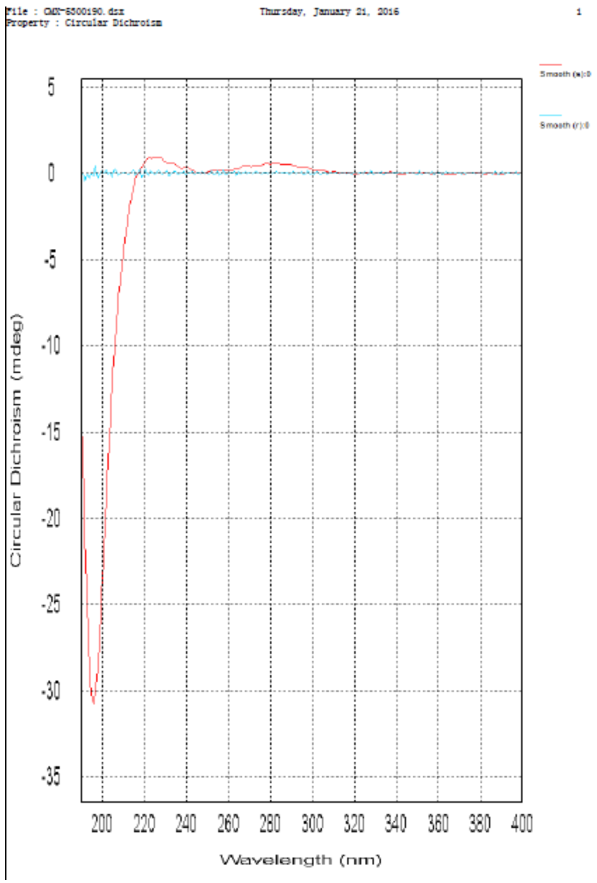

Figure S19. CD spectrum of Vlasouliolide B (**2**) in CH_3CN

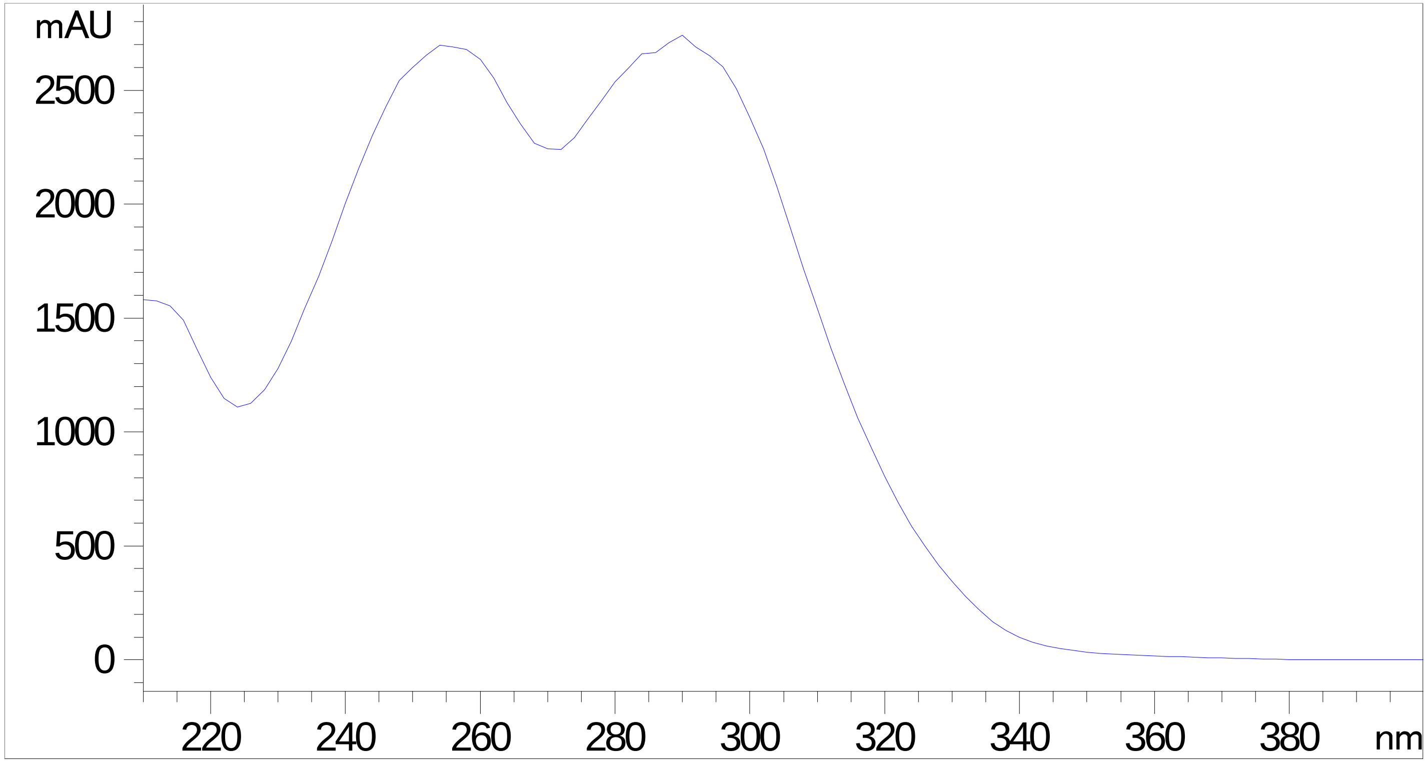

Figure S20. UV spectrum of Vlasouliolide B (**2**) in CH_3CN/H_2O

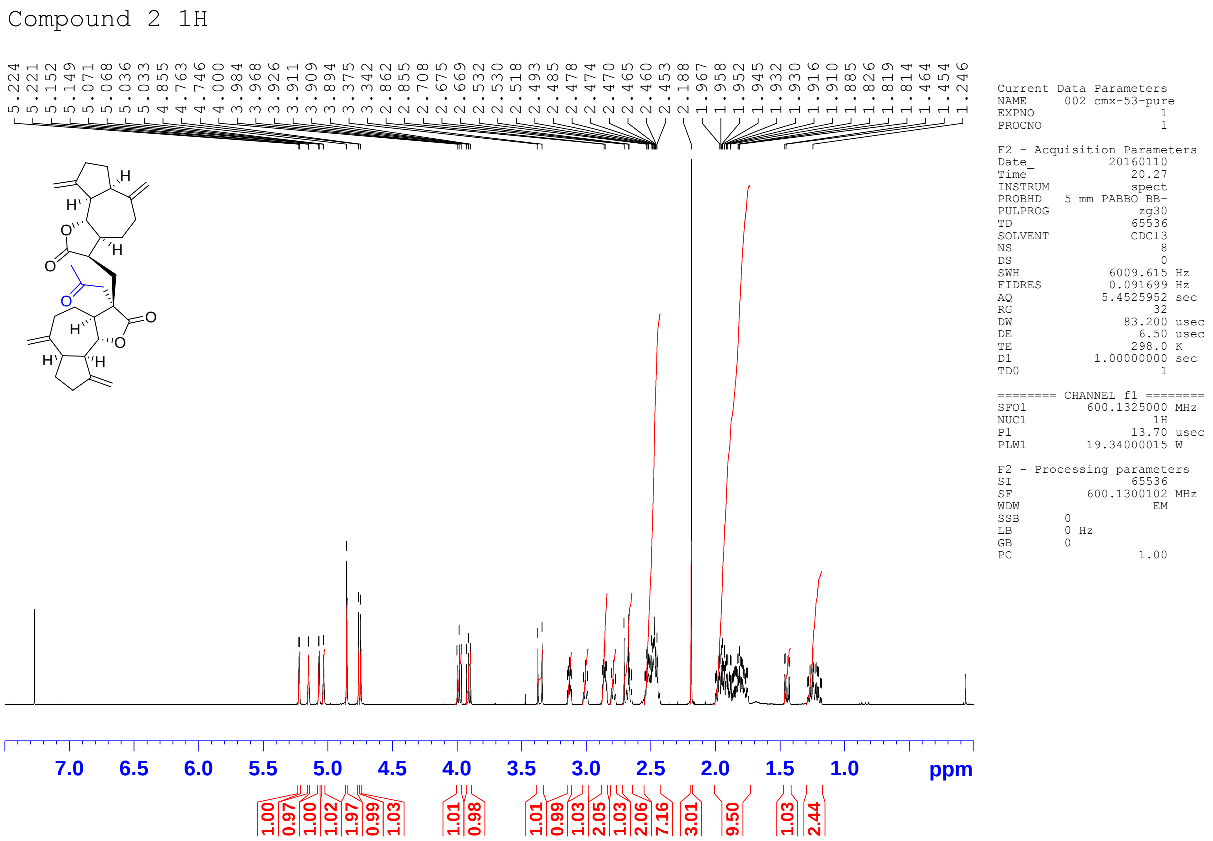

Figure S21. ^{1}H NMR spectrum of Vlasouliolide B (**2**) in $CDCl_3$

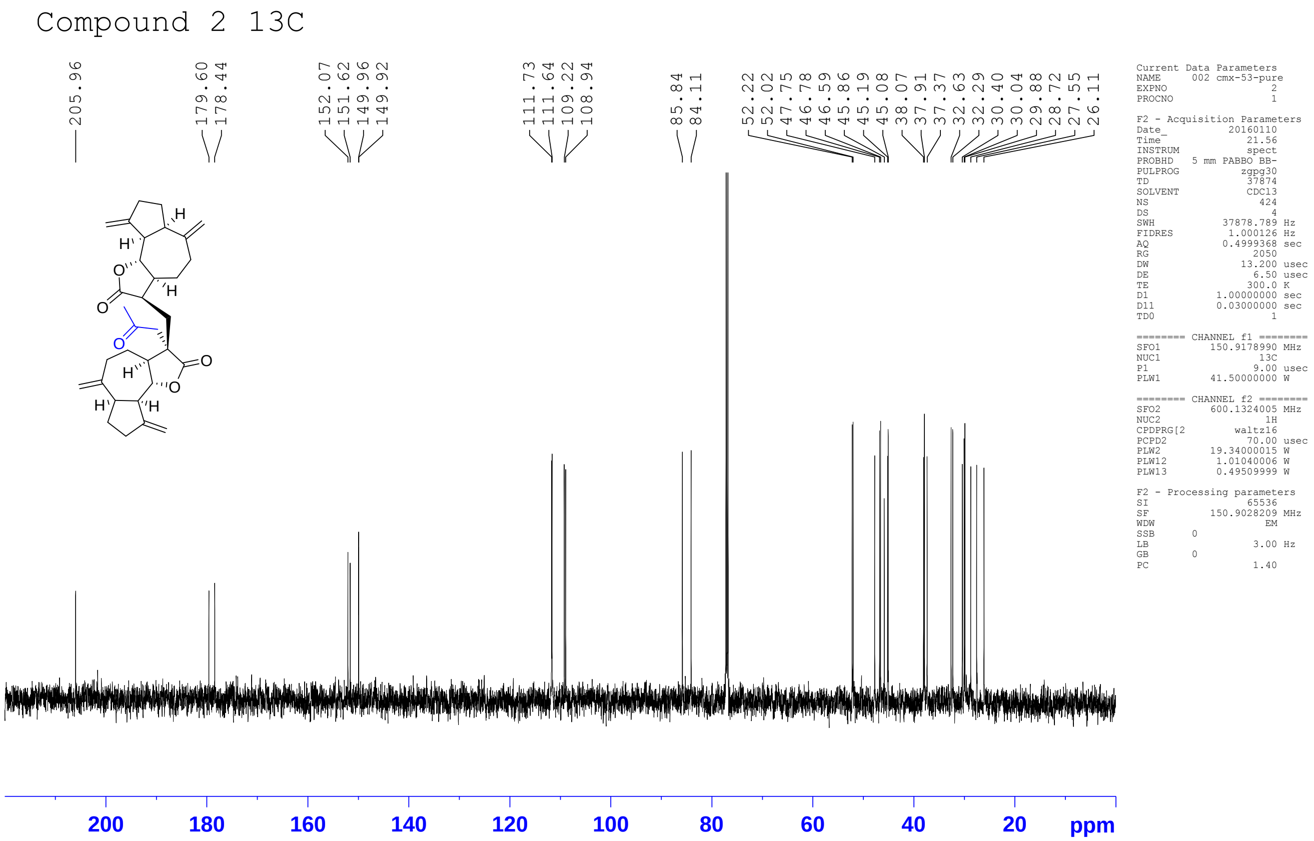

Figure S22. ^{13}C NMR spectra of Vlasouliolide B (**2**) in $CDCl_3$

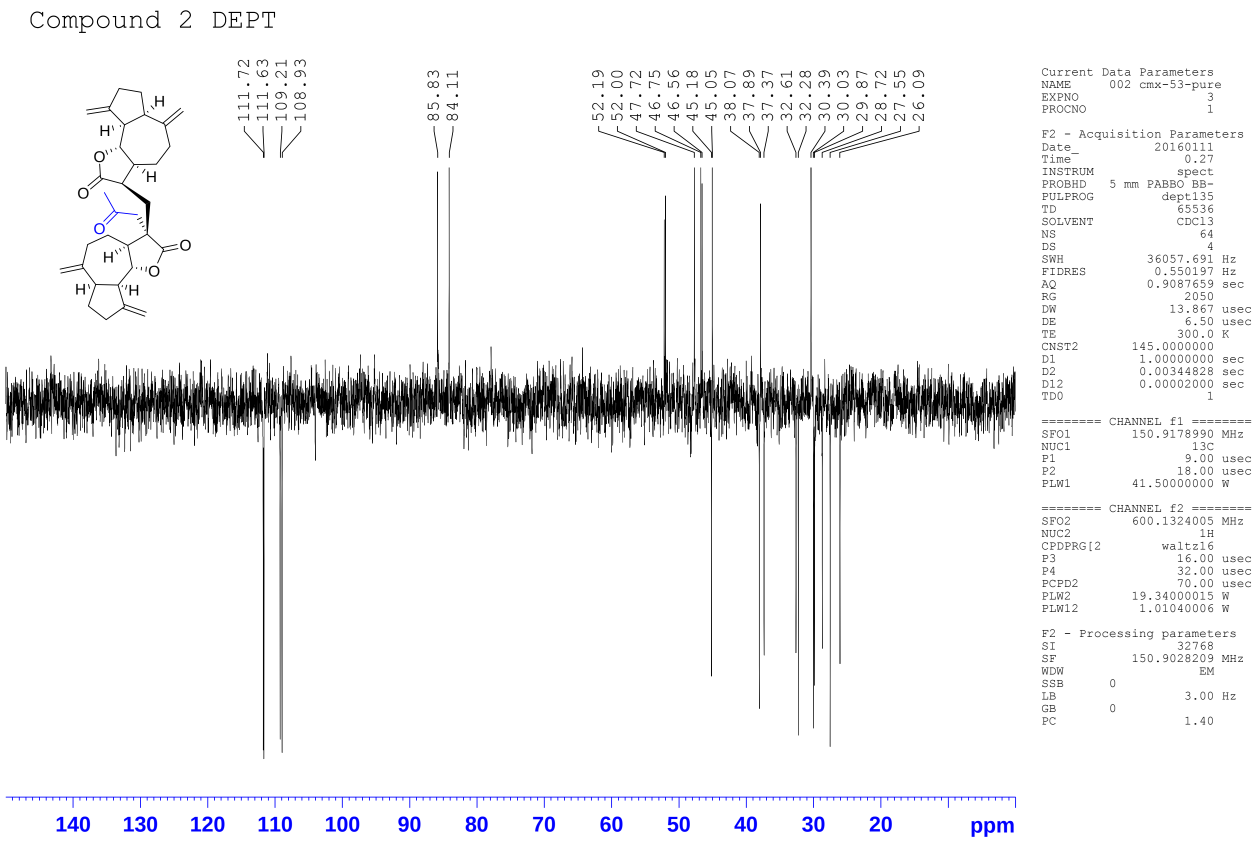

Figure S23. DEPT spectra of Vlasouliolide B (**2**) in $CDCl_3$

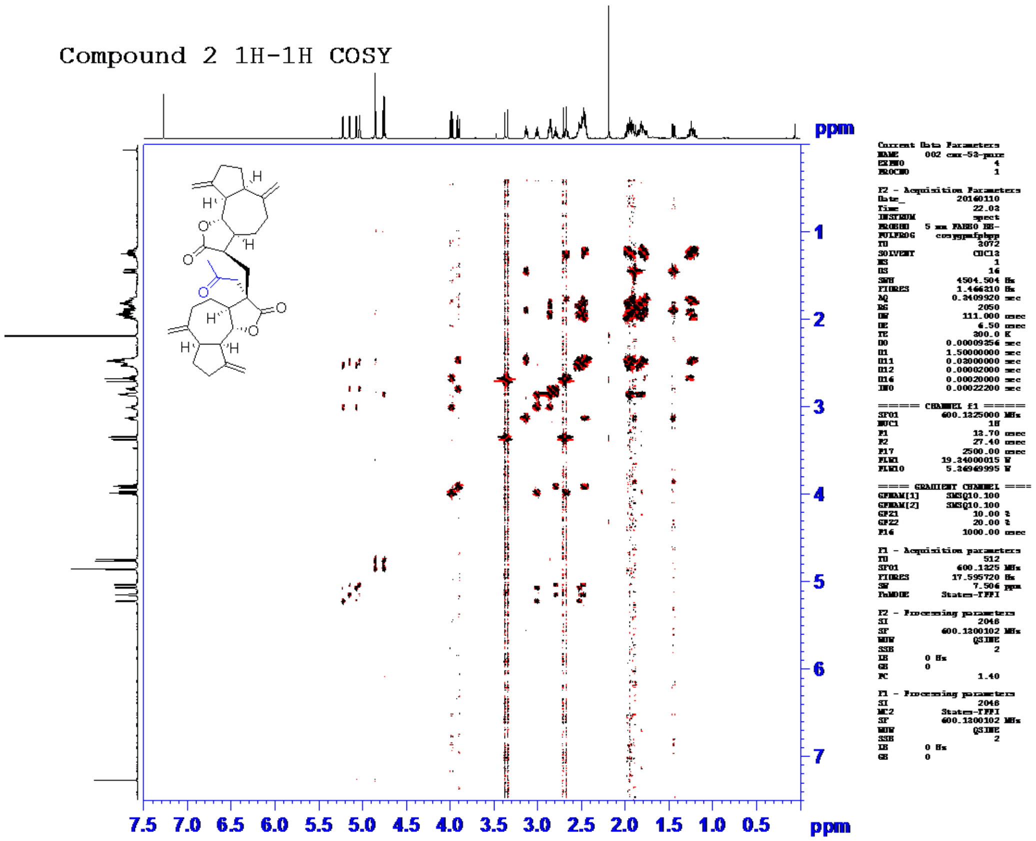

Figure S24. ^{1}H-^{1}H COSY spectrum of Vlasouliolide B (**2**) in $CDCl_3$

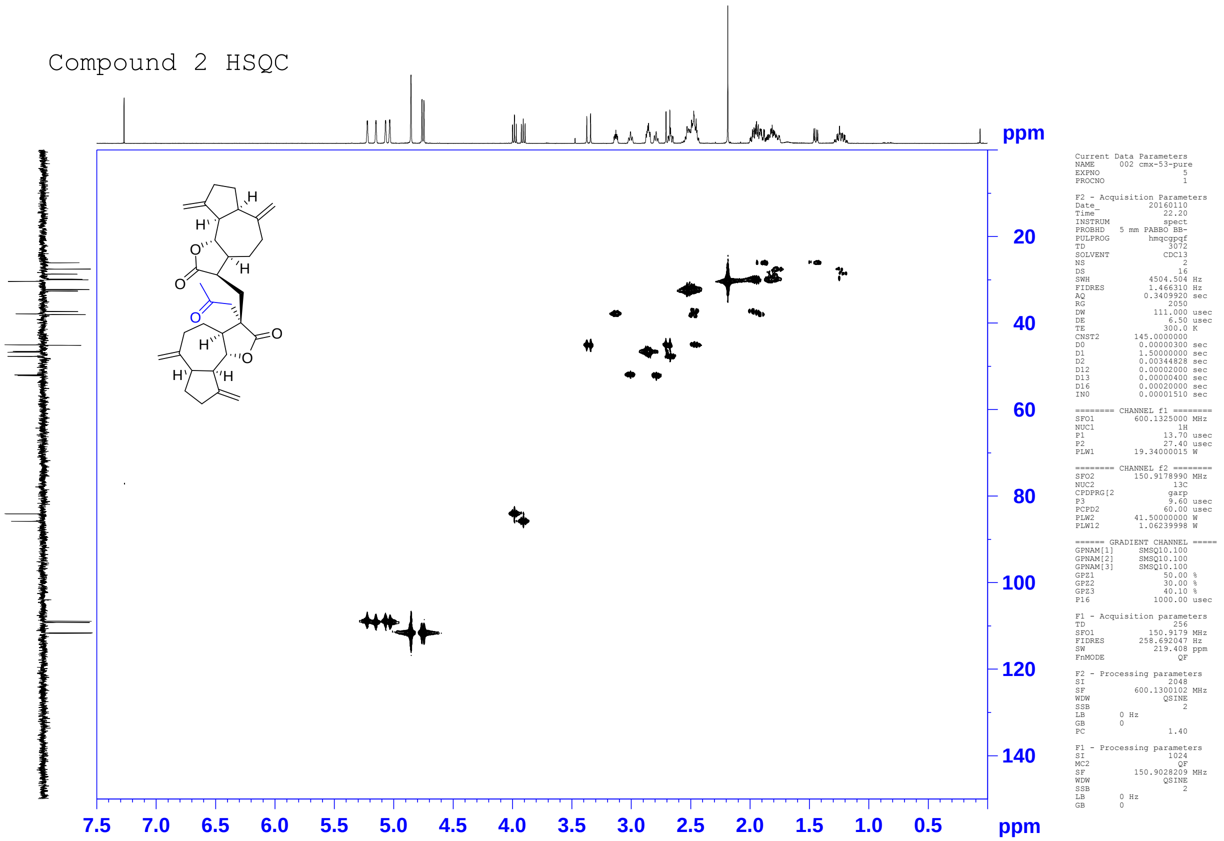

Figure S25. HSQC spectrum of Vlasouliolide B (**2**) in $CDCl_3$

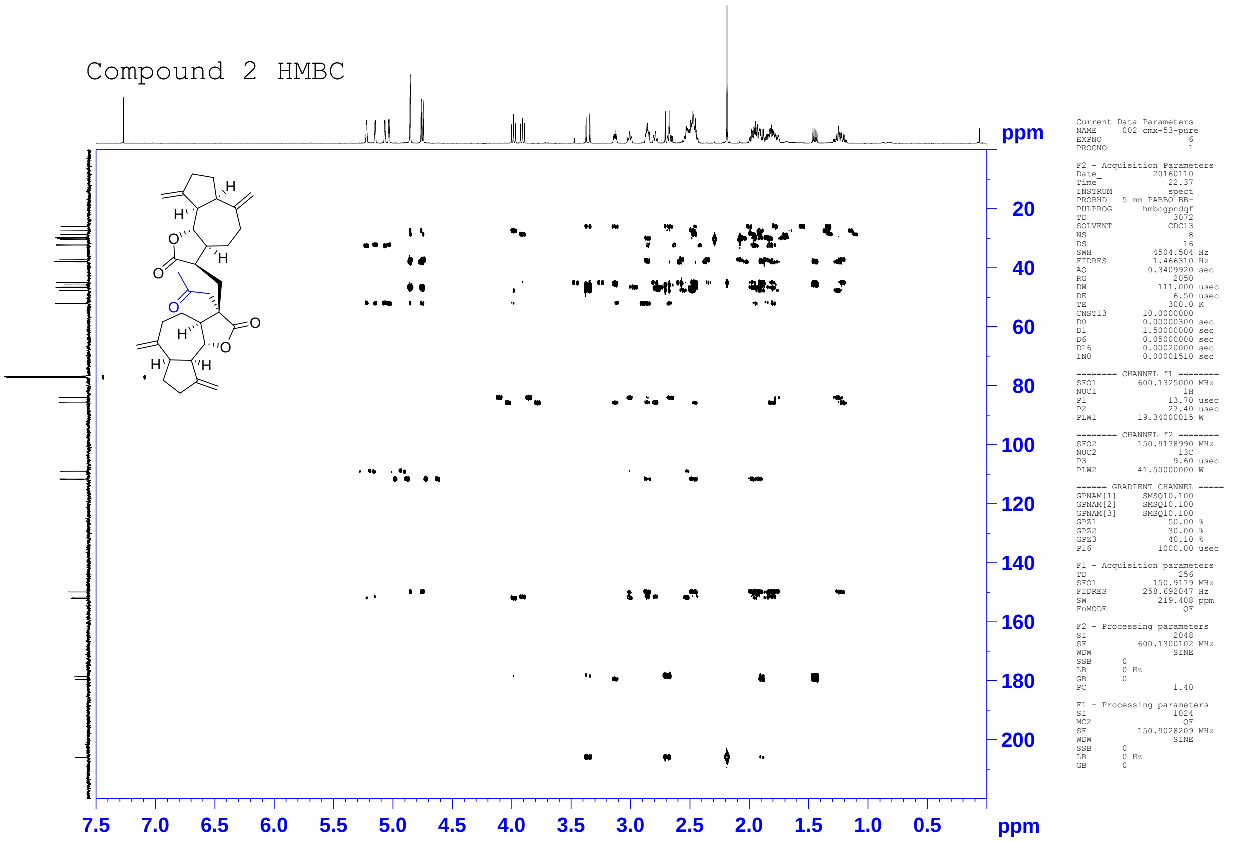

Figure S26. HMBC spectrum of Vlasouliolide B (**2**) in $CDCl_3$

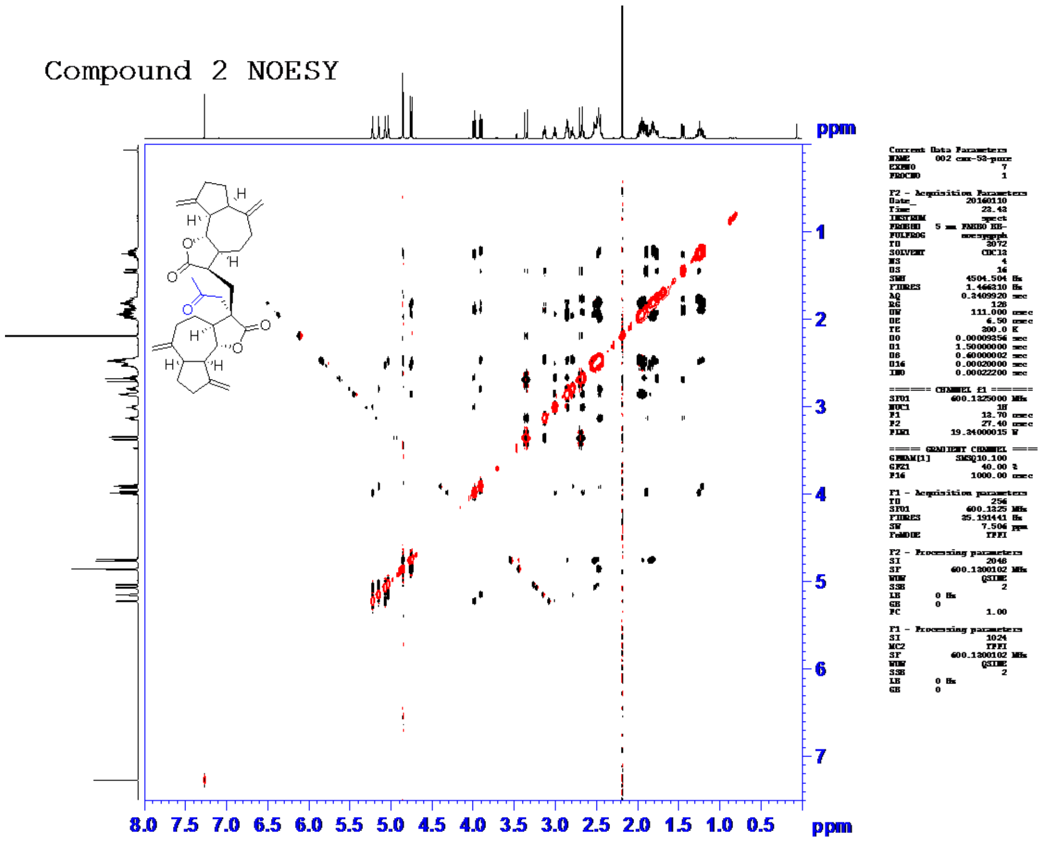

Figure S27. NOESY spectrum of Vlasouliolide B (**2**) in $CDCl_3$

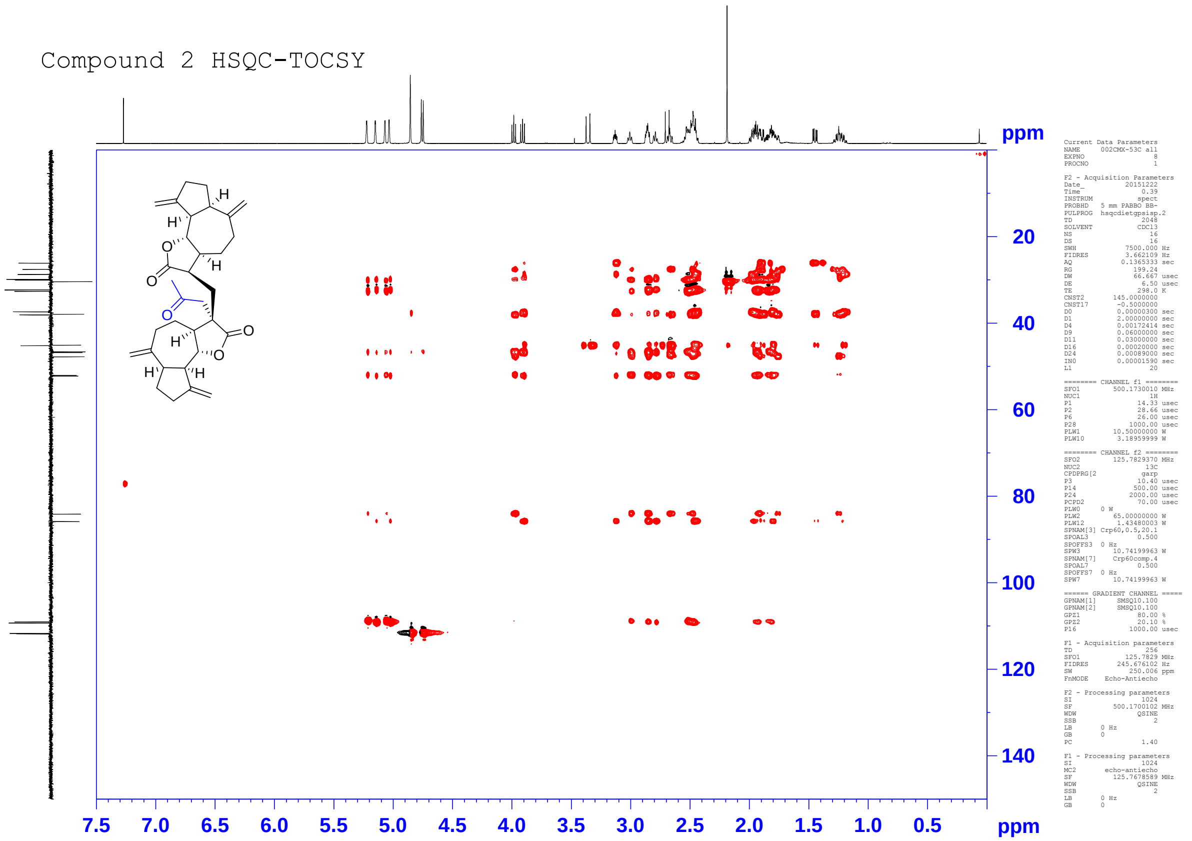

Figure S28. HSQC-TOCSY spectrum of Vlasouliolide B (**2**) in $CDCl_3$

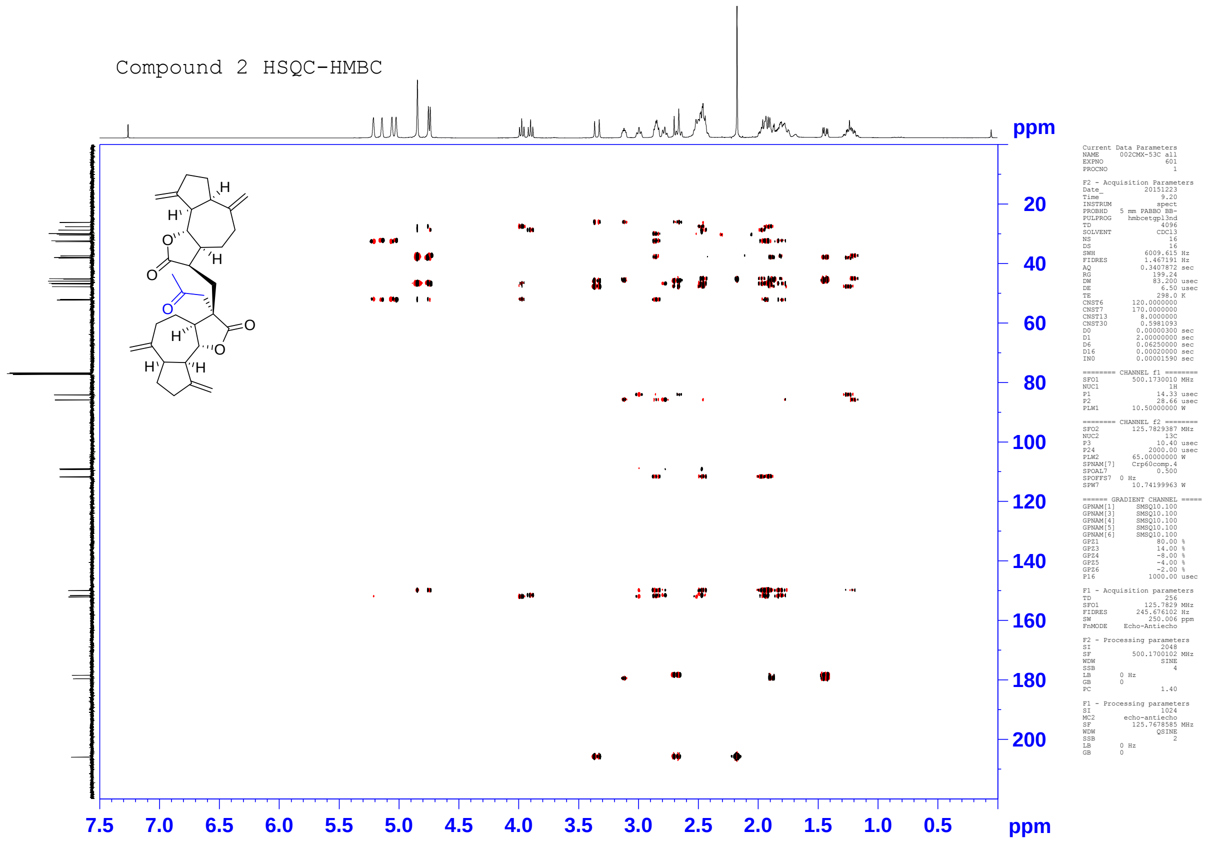

Figure S29. HSQC-HMBC spectrum of Vlasouliolide B (**2**) in CDCl

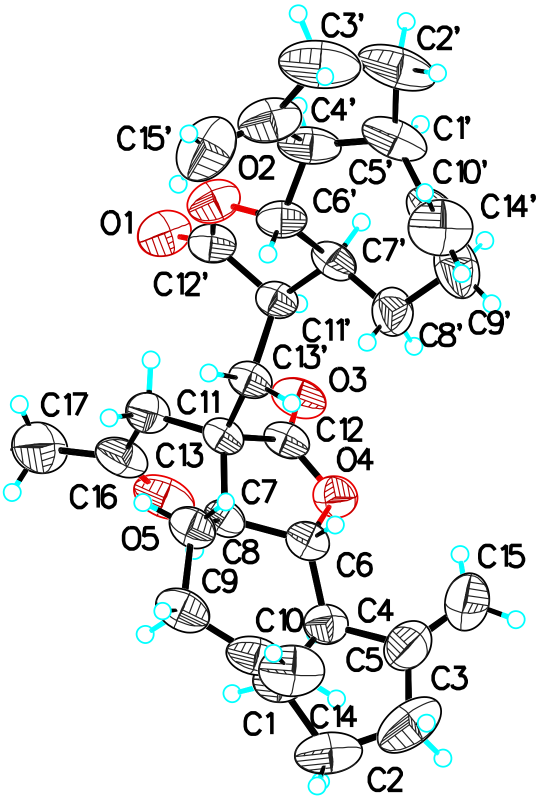

Figure S30. X-ray structure of Vlasouliolide B (**2**)

Table S4. Crystal data and structure refinement for Vlasouliolide B (**2**)

Identification code	cu_dm15891_0m	
Empirical formula	C32 H40 O5	
Formula weight	504.64	
Temperature	296.15 K	
Wavelength	1.54178 Å	
Crystal system	Orthorhombic	
Space group	P 21 21 21	
Unit cell dimensions	a = 11.1725(3) Å	α= 90 °.
	b = 14.2312(4) Å	β= 90 °.
	c = 35.3832(10) Å	γ = 90 °.
Volume	5625.9(3) $Å^3$	
Z	8	
Density (calculated)	1.192 Mg/m^3	
Absorption coefficient	0.629 mm^{-1}	
F(000)	2176	
Crystal size	0.1 x 0.08 x 0.05 mm^3	
Theta range for data collection	2.497 to 70.111 °.	
Index ranges	-13<=h<=13, -17<=k<=17, -43<=l<=41	
Reflections collected	39132	
Independent reflections	10295 [R(int) = 0.0615]	
Completeness to theta = 67.679 °	99.8 %	
Absorption correction	Semi-empirical from equivalents	
Max. and min. transmission	0.7533 and 0.6182	
Refinement method	Full-matrix least-squares on F^2	
Data / restraints / parameters	10295 / 0 / 669	
Goodness-of-fit on F^2	1.070	
Final R indices [I>2sigma(I)]	R1 = 0.0570, wR2 = 0.1518	
R indices (all data)	R1 = 0.0749, wR2 = 0.1664	
Absolute structure parameter	0.10(15)	
Extinction coefficient	n/a	
Largest diff. peak and hole	0.230 and -0.183 e.$Å^{-3}$	

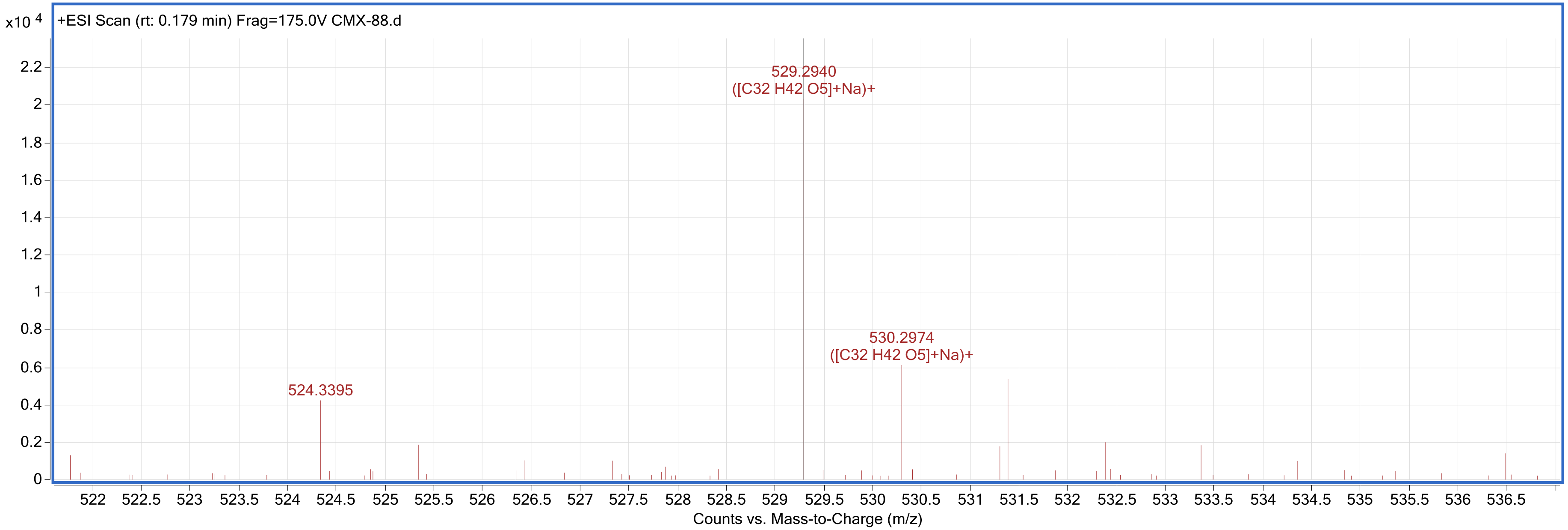

Best	Formula (M)	Ion Formula	m/z	Calc m/z	Score	Mass	Calc Mass	Diff (ppm)
TRUE	$C_{32}H_{42}O_5$	$C_{32}H_{42}NaO_5$	529.2940	529.2930	86.96	506.305	506.30	-3.42

Figure S31. HRESIMS spectrum of Vlasouliolide C (**3**)

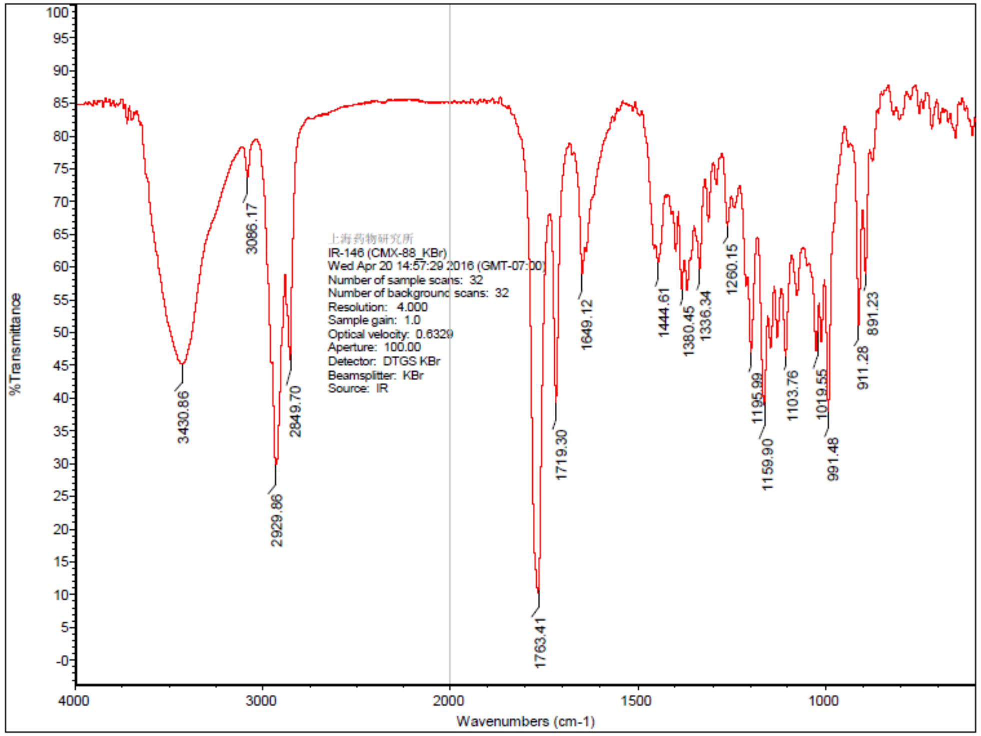

The IR absorption at 3430.86 cm^{-1} was attributed to residual solvent or impurity.

Figure S32. IR spectrum of Vlasouliolide C (**3**)

Rudolph Research Analytical

Thursday, 04/21/2016

This sample was measured on an Autopol VI, serial number 90079,
manufactured by Rudolph Research Analytical,Hackettstown,NJ.

LotID : CMX-88
Set Temperature : 20.0
Temp Corr : OFF

n	Average	Std.Dev.	Maximum	Minimum
5	30.233	0.0000	30.233	30.233

S.No	Sample ID	Time	Result	Scale	OR ° Arc	WLG	Lg.mm	Conc.	Temp.	Comment
1	CMX-88	04:53:08 PM	30.233	SR	0.013	589	100.00	0.043	19.7	
2	CMX-88	04:53:15 PM	30.233	SR	0.013	589	100.00	0.043	19.7	
3	CMX-88	04:53:23 PM	30.233	SR	0.013	589	100.00	0.043	19.7	
4	CMX-88	04:53:30 PM	30.233	SR	0.013	589	100.00	0.043	19.7	
5	CMX-88	04:53:37 PM	30.233	SR	0.013	589	100.00	0.043	19.7	

____
Signature

Figure S33. OR Value of Vlasouliolide C (**3**) in $CHCl_3$

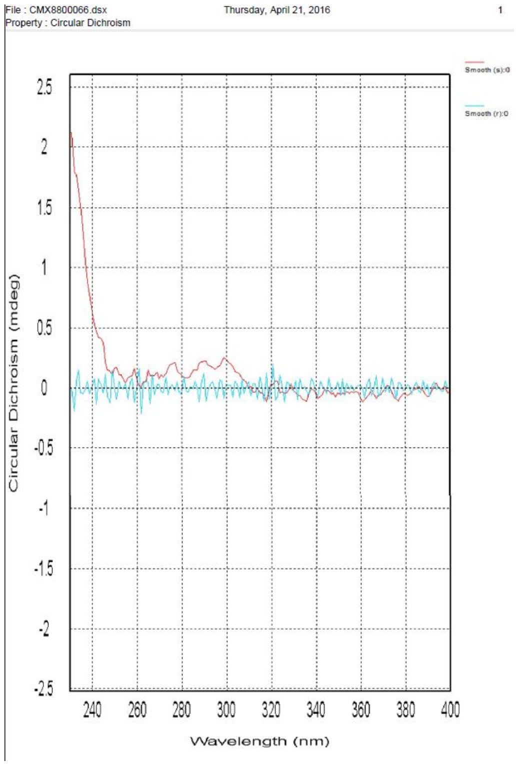

Figure S34. CD spectrum of Vlasouliolide C (**3**) in CH_3COCH_3

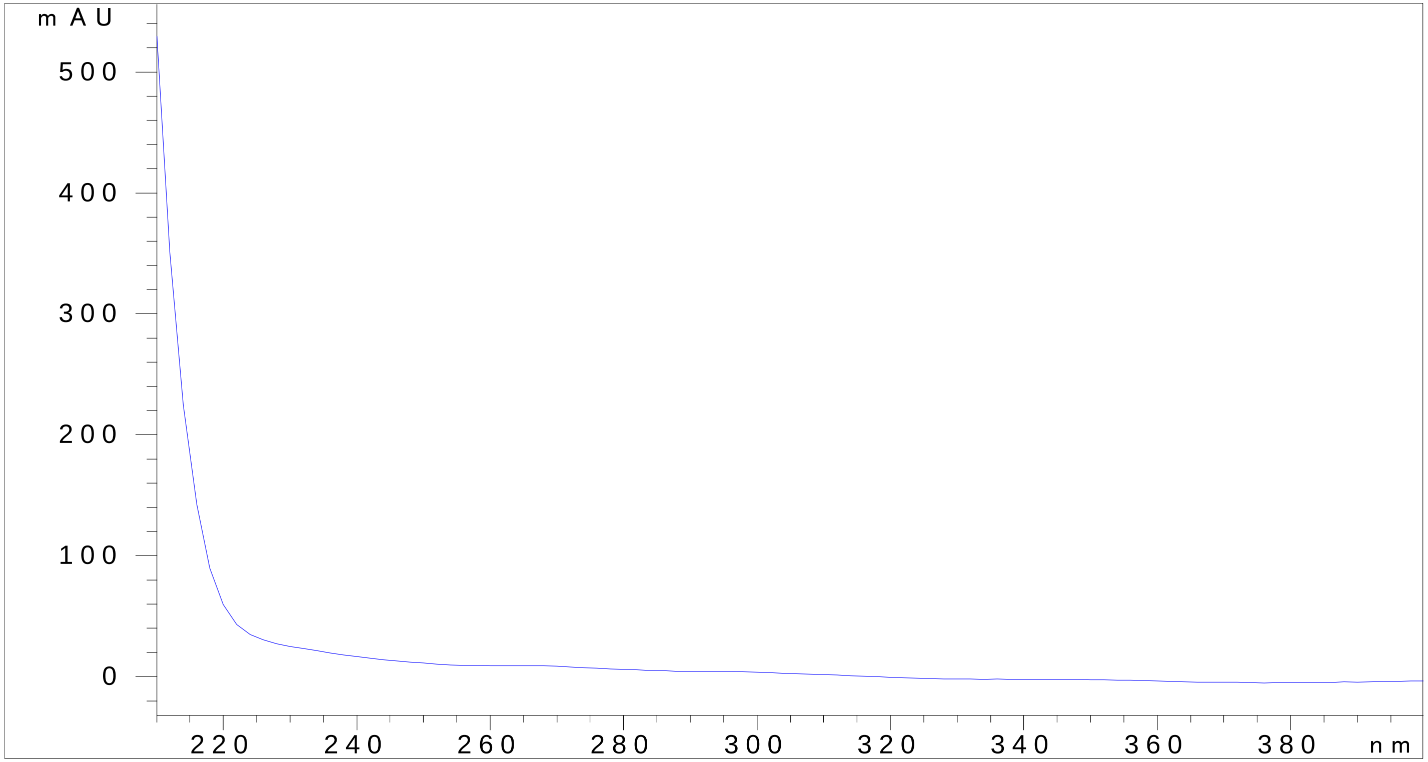

Figure S35. UV spectrum of Vlasouliolide C (**3**) in CH_3CN/H_2O

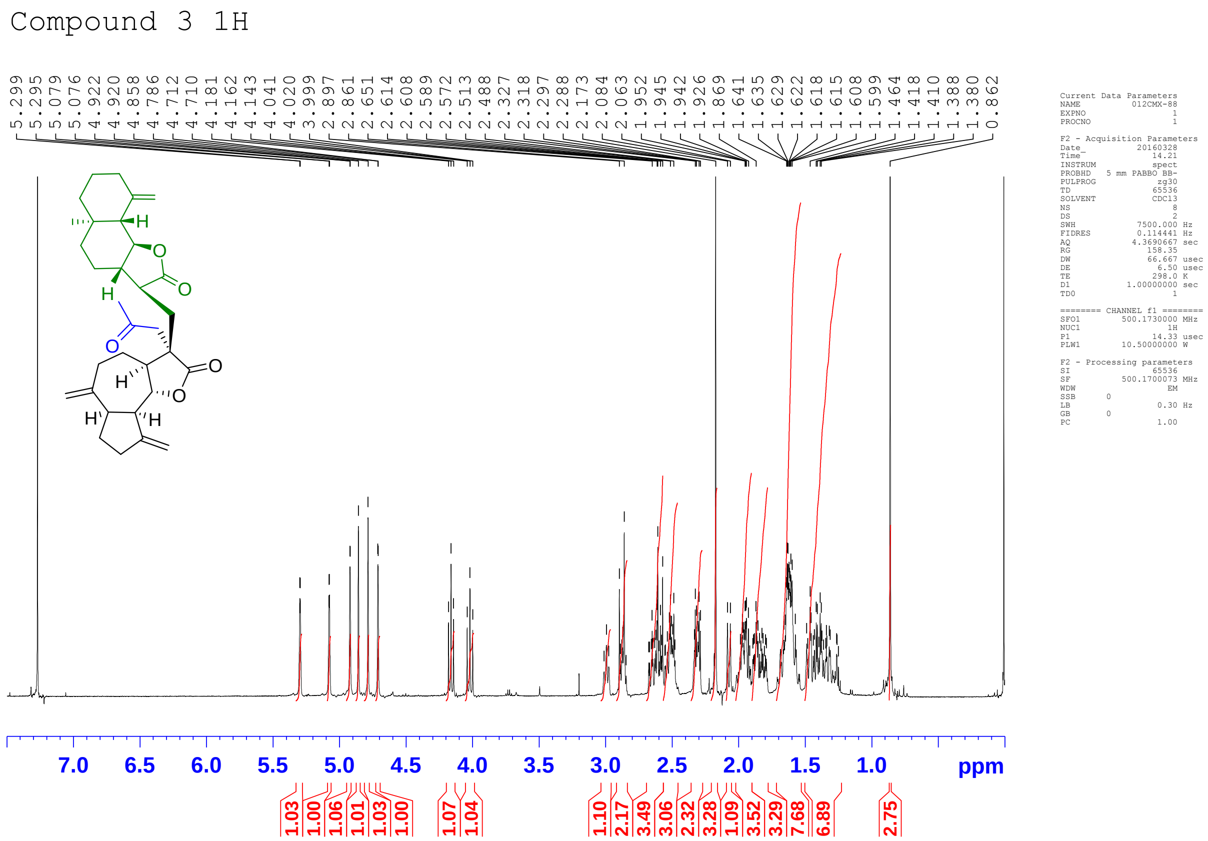

Figure S36. ^{1}H NMR spectrum of Vlasouliolide C (**3**) in $CDCl_3$

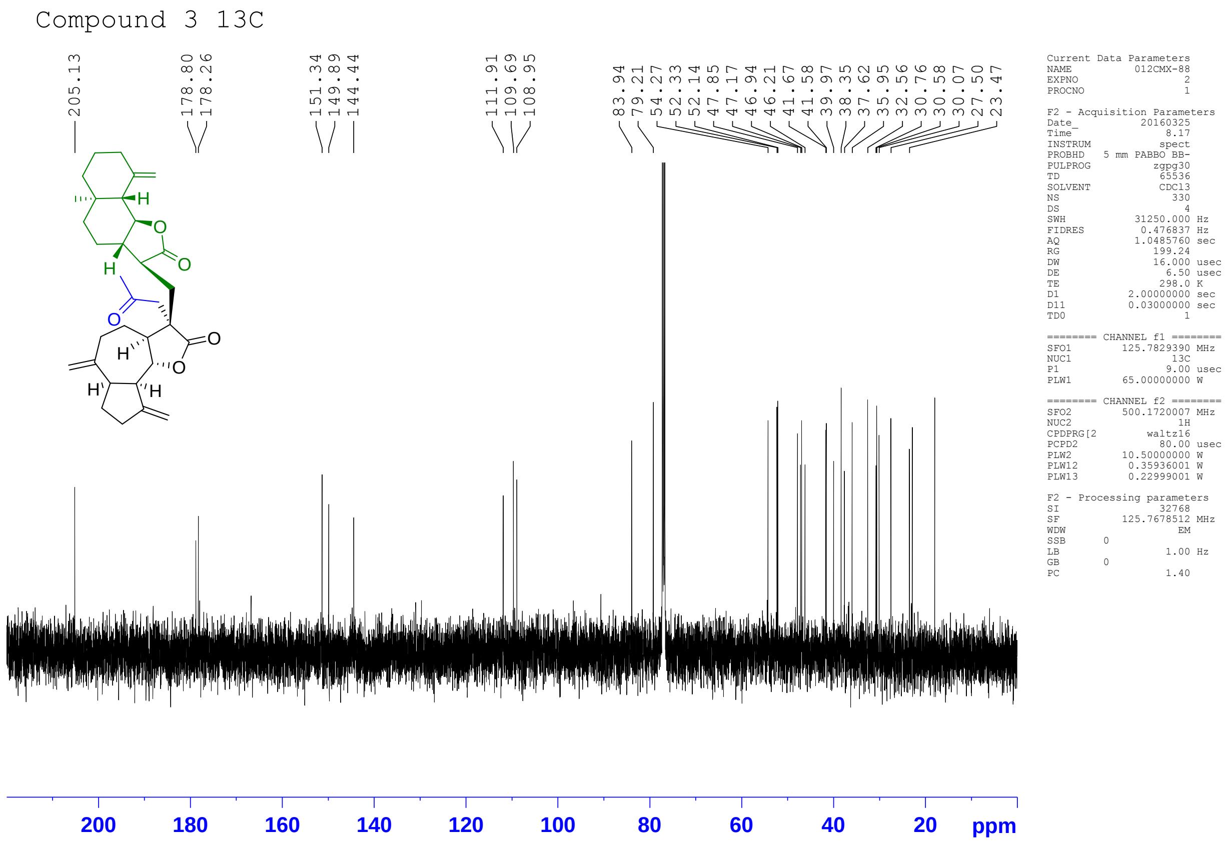

Figure S37. ^{13}C NMR spectra of Vlasouliolide C (**3**) in $CDCl_3$

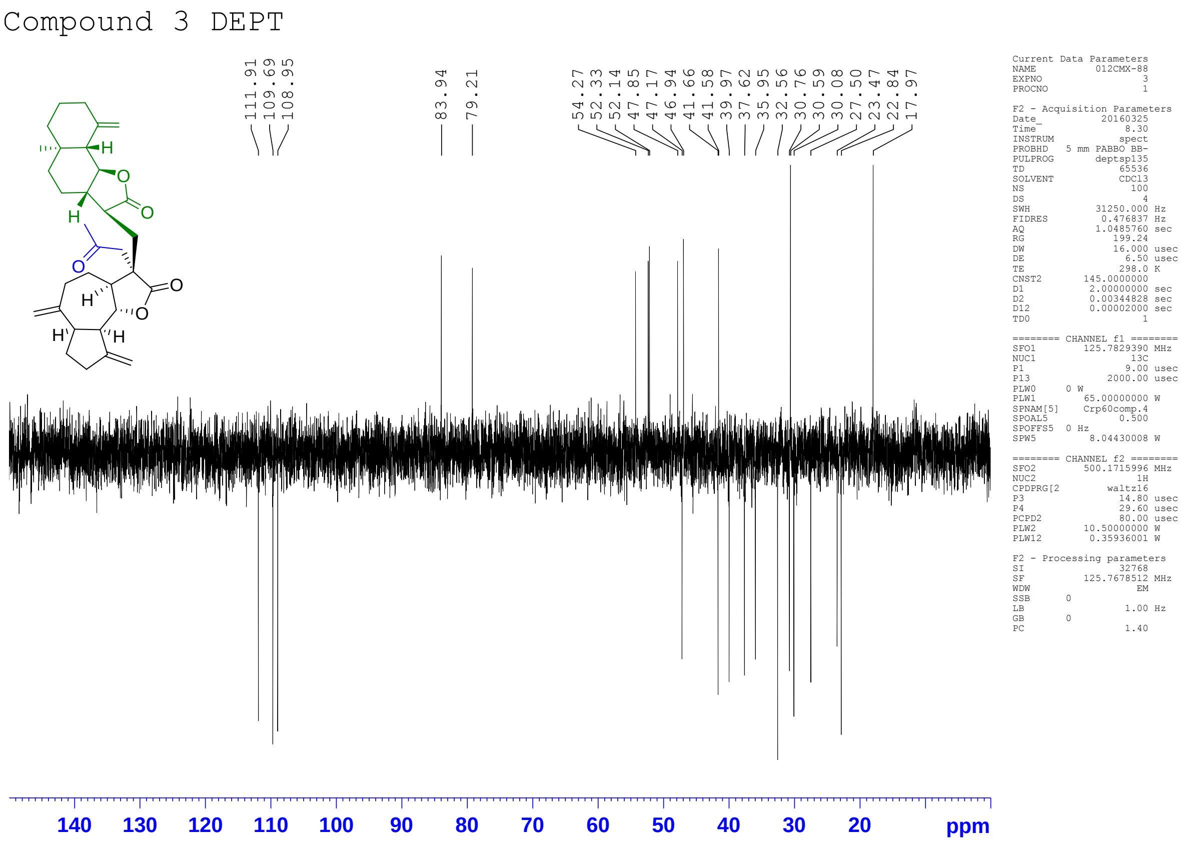

Figure S38. DEPT spectra of Vlasouliolide C (**3**) in $CDCl_3$

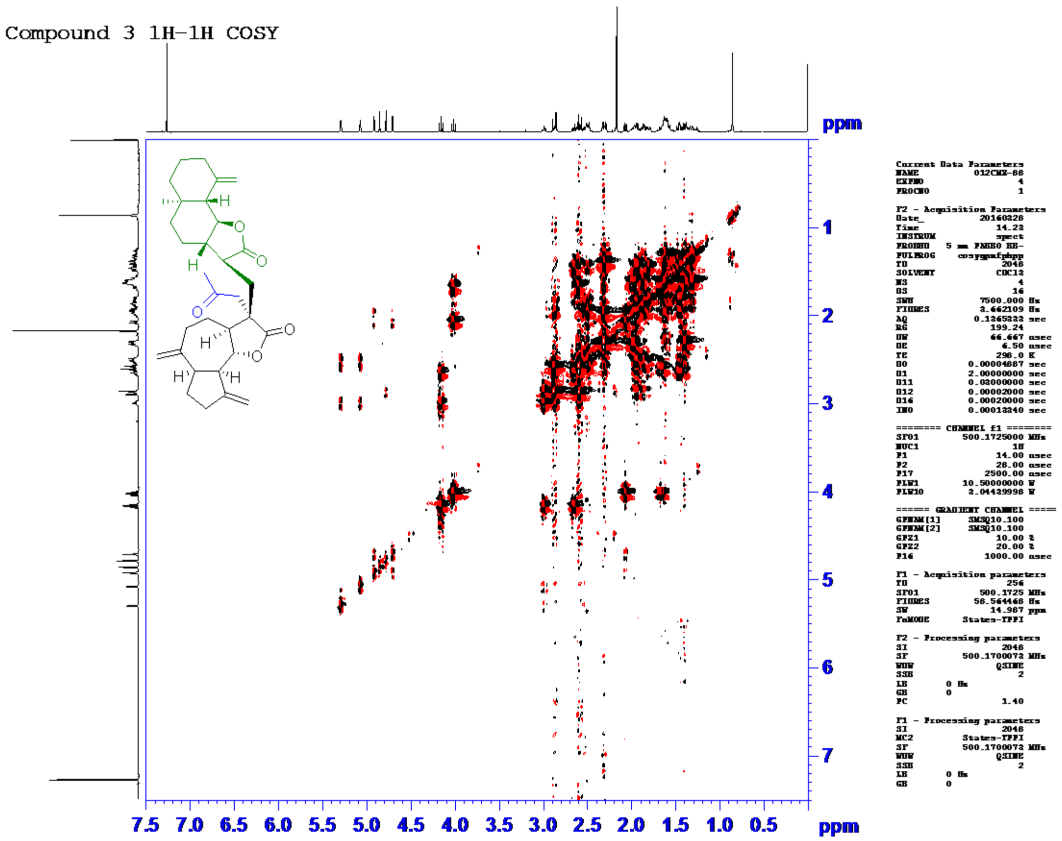

Figure S39. ^{1}H-^{1}H COSY spectrum of Vlasouliolide C (**3**) in $CDCl_3$

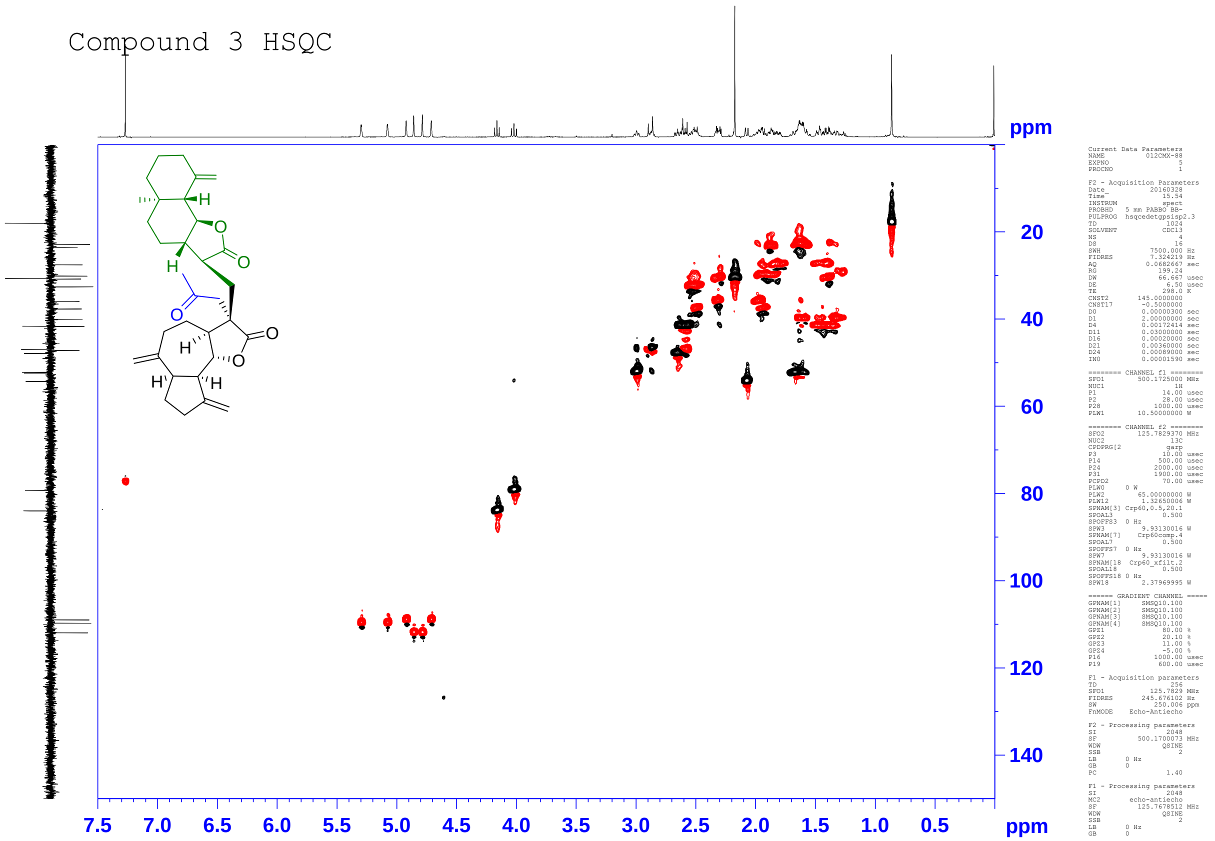

Figure S40. HSQC spectrum of Vlasouliolide C (**3**) in $CDCl_3$

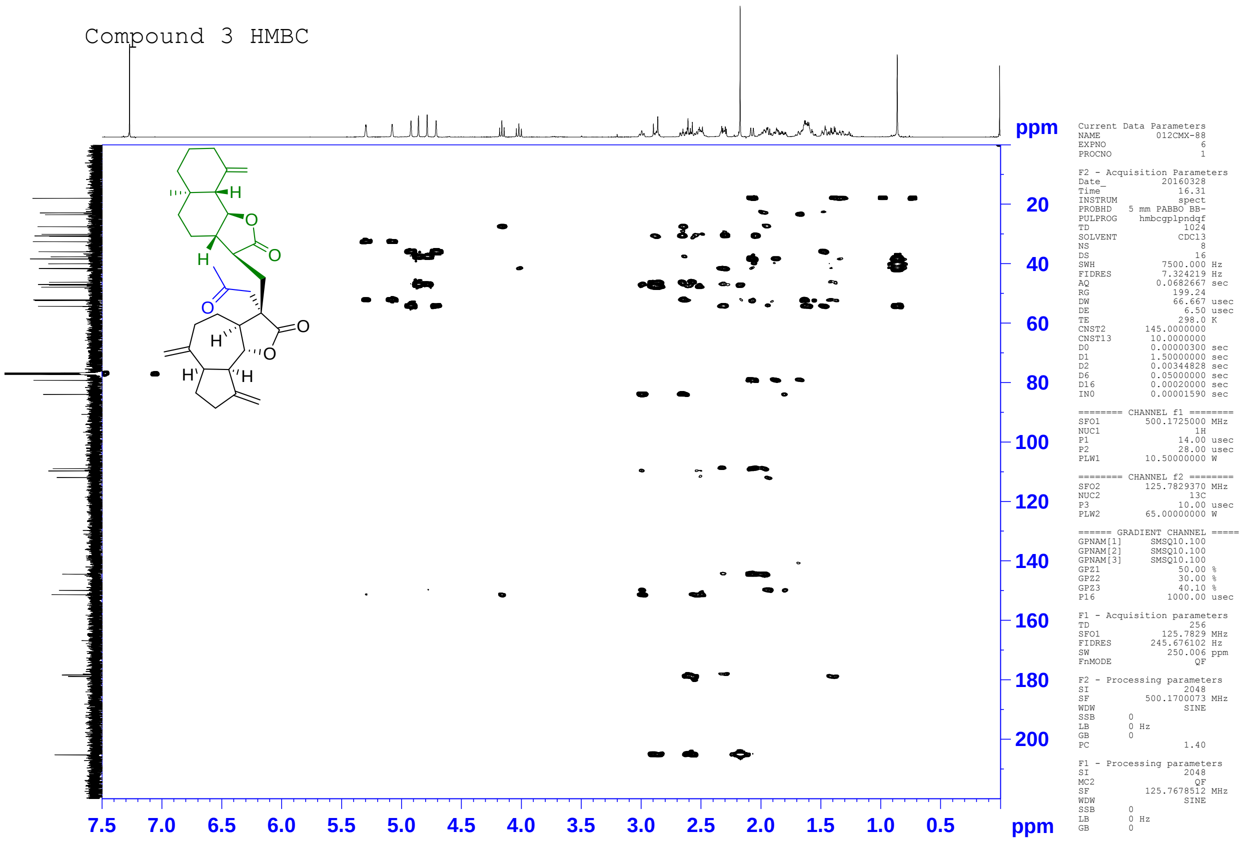

Figure S41. HMBC spectrum of Vlasouliolide C (**3**) in $CDCl_3$

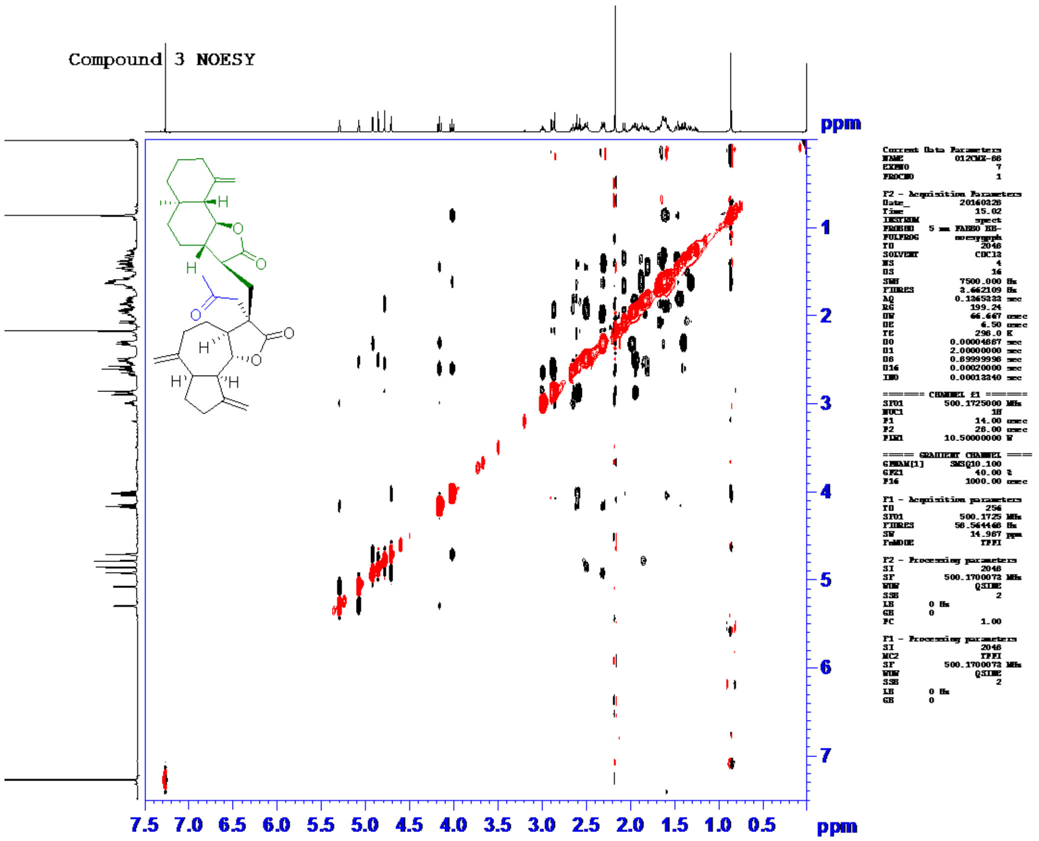

Figure S42. NOESY spectrum of Vlasouliolide C (**3**) in $CDCl_3$

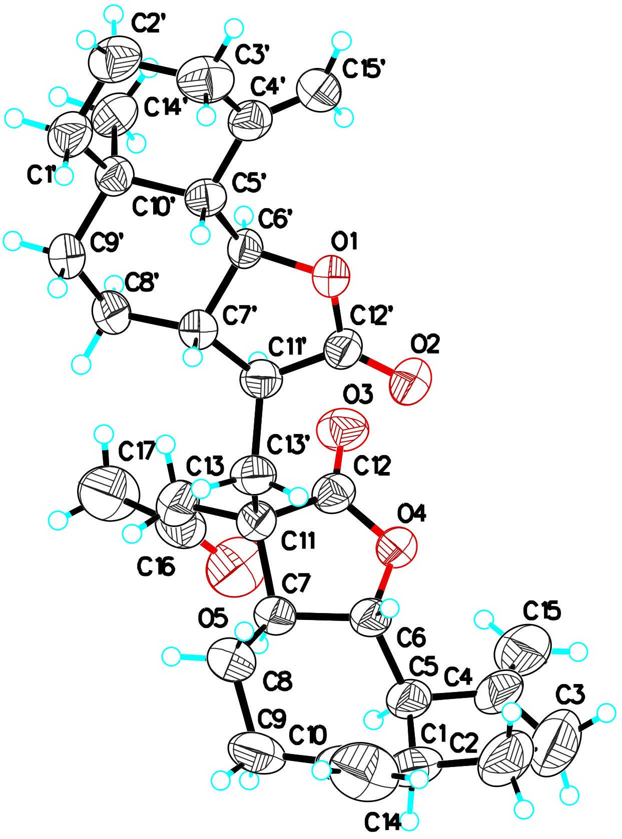

Figure S43. X-ray structure of Vlasouliolide C (**3**)

Table S5. Crystal data and structure refinement for Vlasouliolide C (**3**)

Identification code	cu_dm16188_0m	
Empirical formula	C32 H42 O5	
Formula weight	506.65	
Temperature	296 K	
Wavelength	1.54178 Å	
Crystal system	Monoclinic	
Space group	P 1 21 1	
Unit cell dimensions	a = 9.1953(2) Å	α= 90 °.
	b = 7.6570(2) Å	β= 99.956(2) °.
	c = 19.9371(2) Å	γ = 90 °.
Volume	1382.60(5) $Å^3$	
Z	2	
Density (calculated)	1.217 Mg/m^3	
Absorption coefficient	0.640 mm^{-1}	
F(000)	548	
Crystal size	0.12 x 0.05 x 0.01 mm^3	
Theta range for data collection	4.503 to 69.439 °.	
Index ranges	-11<=h<=11, -8<=k<=7, -23<=l<=24	
Reflections collected	10143	
Independent reflections	3967 [R(int) = 0.0452]	
Completeness to theta = 67.679 °	97.1 %	
Absorption correction	Semi-empirical from equivalents	
Max. and min. transmission	0.7532 and 0.5641	
Refinement method	Full-matrix least-squares on F^2	
Data / restraints / parameters	3967 / 1 / 336	
Goodness-of-fit on F^2	1.042	
Final R indices [I>2sigma(I)]	R1 = 0.0425, wR2 = 0.1055	
R indices (all data)	R1 = 0.0494, wR2 = 0.1108	
Absolute structure parameter	0.4(2)	
Extinction coefficient	n/a	
Largest diff. peak and hole	0.136 and -0.170 e.$Å^{-3}$	

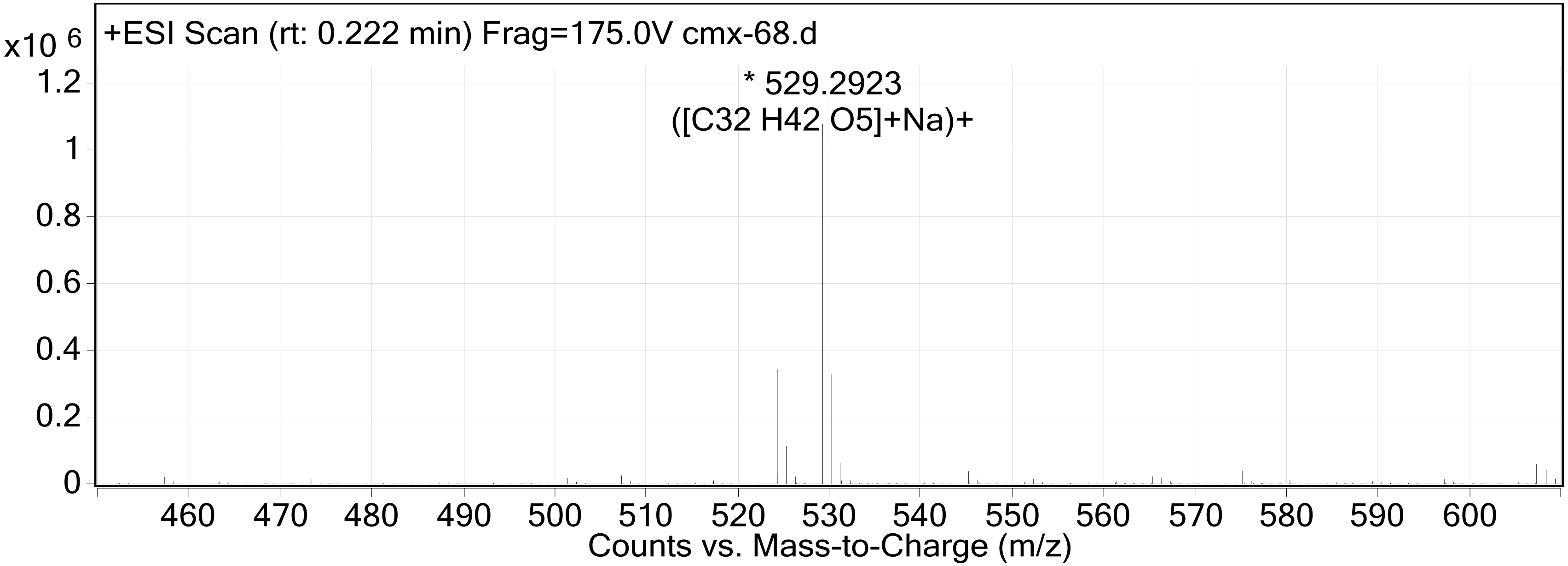

Best	Formula (M)	Ion Formula	m/z	Calc m/z	Score	Mass	Calc Mass	Diff (ppm)
TRUE	$C_{32}H_{42}O_5$	$C_{32}H_{45}NaO_5$	529.2923	529.2930	96.23	506.3029	506.30	0.58

Figure S44. HRESIMS spectrum of Vlasouliolide D (**4**)

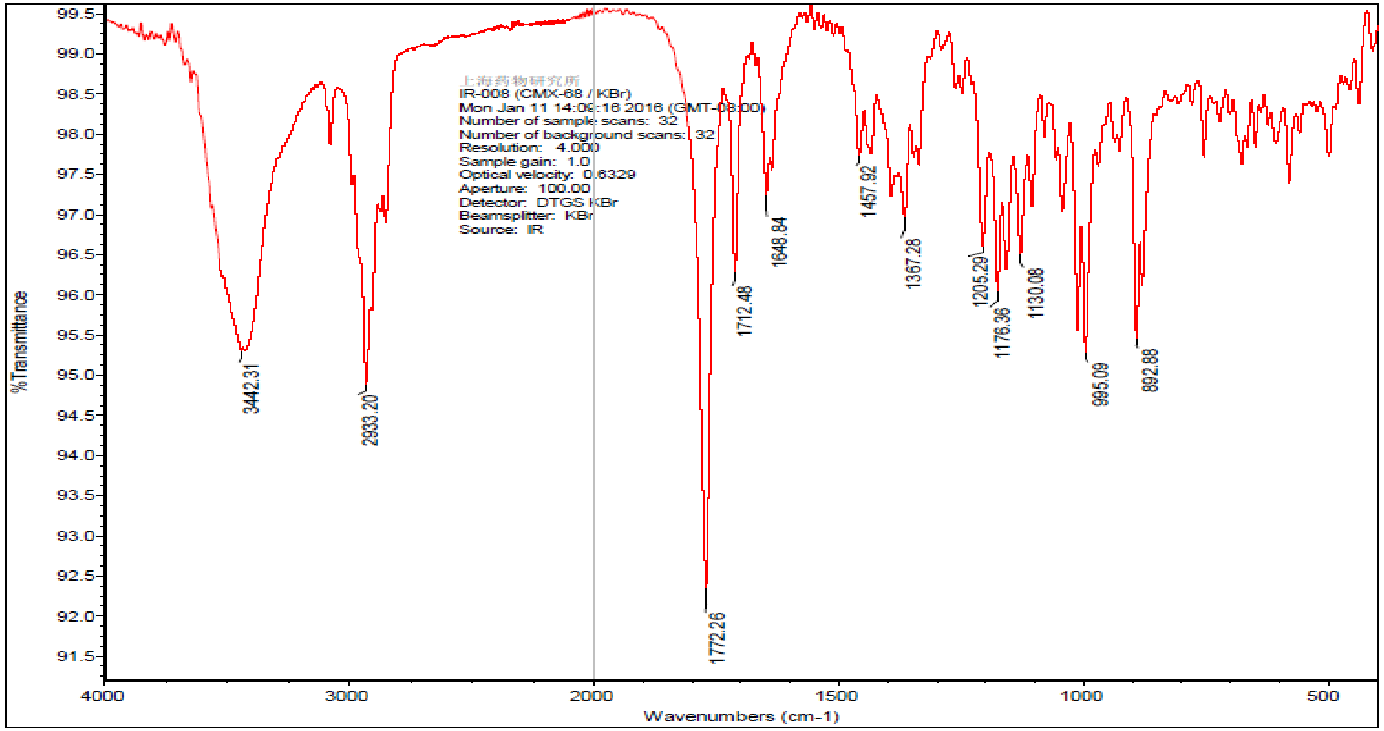

The IR absorption at 3442.31 cm^{-1} was attributed to residual solvent or impurity.

Figure S45. IR spectrum of Vlasouliolide D (**4**)

Rudolph Research Analytical

Friday, 01/22/2016

This sample was measured on an Autopol VI, serial number 90079,
manufactured by Rudolph Research Analytical,Hackettstown,NJ.

LotID : CMX-68
Set Temperature : 20.0
Temp Corr : OFF

n	Average	Std.Dev.	Maximum	Minimum
6	55.208	0.9410	56.250	53.750

S.No	Sample ID	Time	Result	Scale	OR ° Arc	WLG	Lg.mm	Conc.	Temp.	Comment
1	CMX-68	01:30:57 PM	55.000	SR	0.044	589	100.00	0.080	20.2	
2	CMX-68	01:31:04 PM	55.000	SR	0.044	589	100.00	0.080	20.1	
3	CMX-68	01:31:11 PM	55.000	SR	0.044	589	100.00	0.080	20.1	
4	CMX-68	01:31:18 PM	53.750	SR	0.043	589	100.00	0.080	20.1	
5	CMX-68	01:31:26 PM	56.250	SR	0.045	589	100.00	0.080	20.1	
6	CMX-68	01:31:32 PM	56.250	SR	0.045	589	100.00	0.080	20.0	

Signature

Figure S46. OR Value of Vlasouliolide D (**4**) in CH_3COCH_3

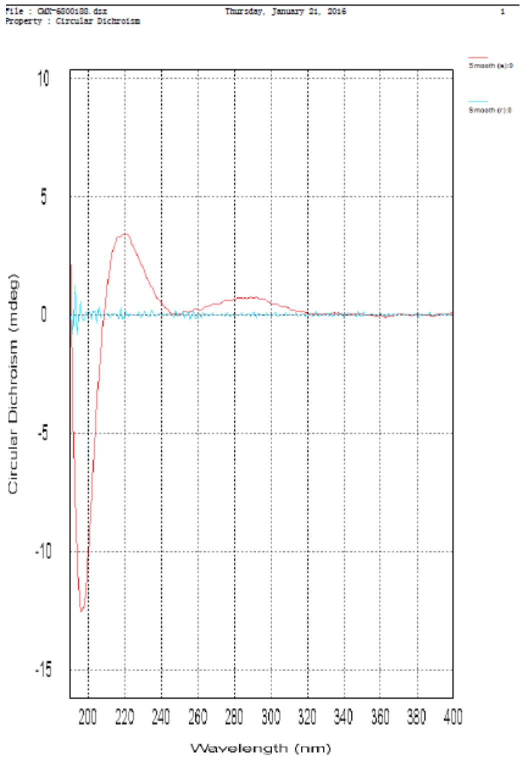

Figure S47. CD spectrum of Vlasouliolide D (**4**) in CH_3CN

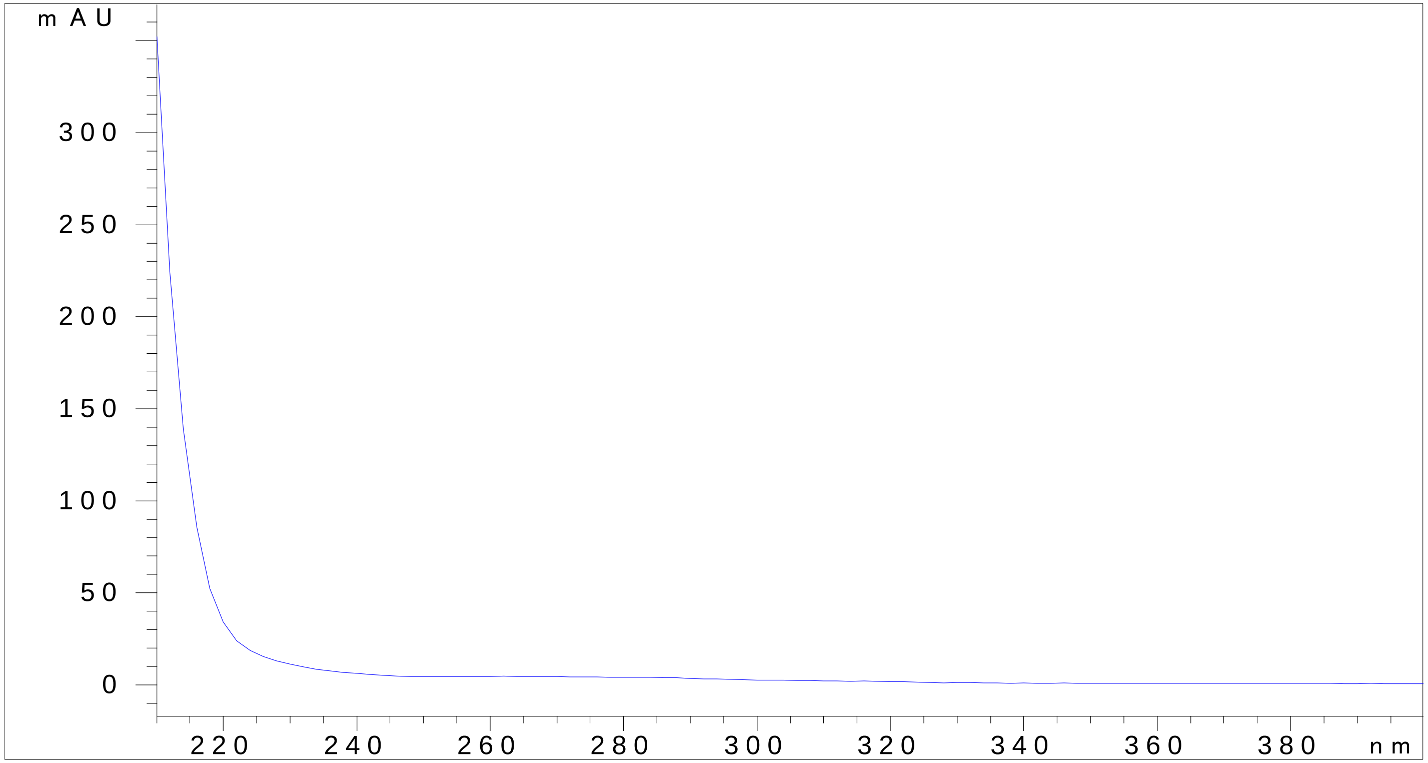

Figure S48. UV spectrum of Vlasouliolide D (**4**) in CH_3CN/H_2O

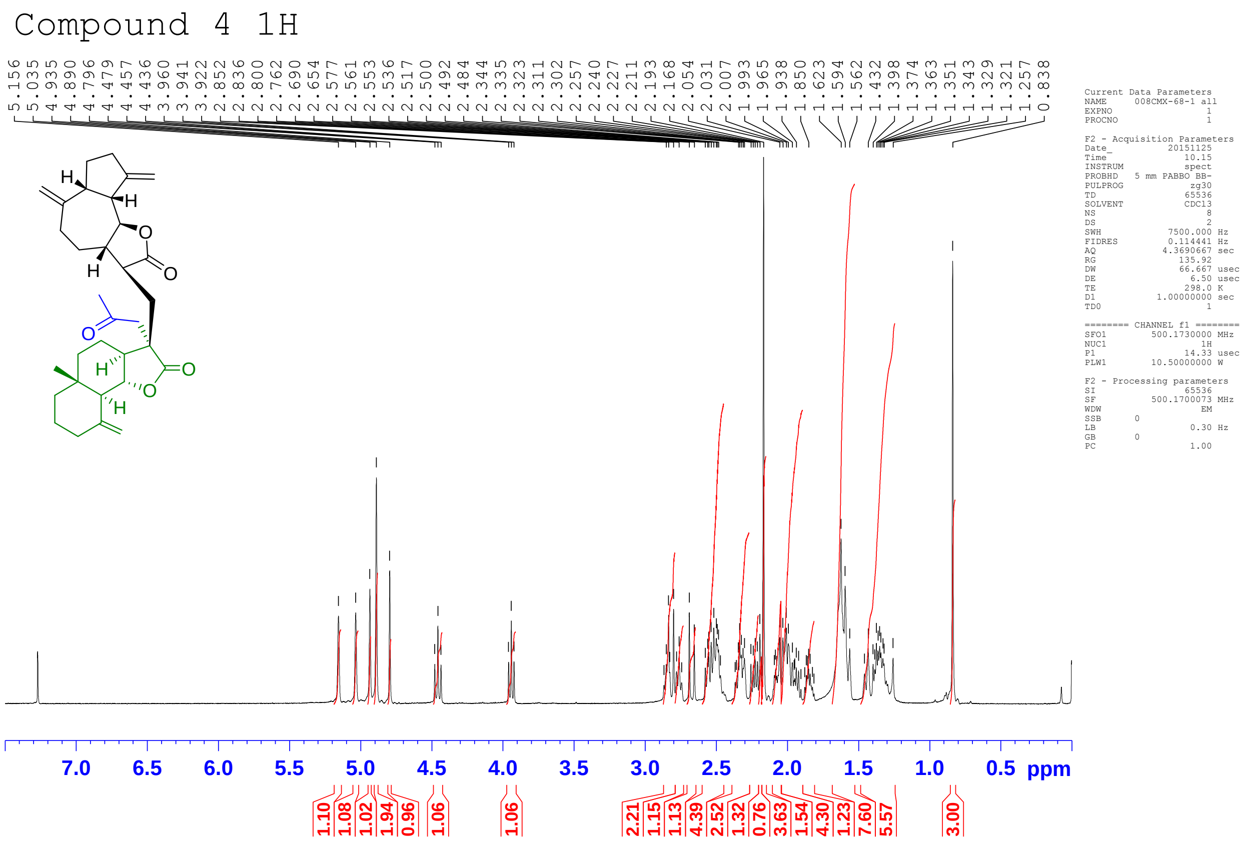

Figure S49. ^{1}H NMR spectrum of Vlasouliolide D (**4**) in $CDCl_3$

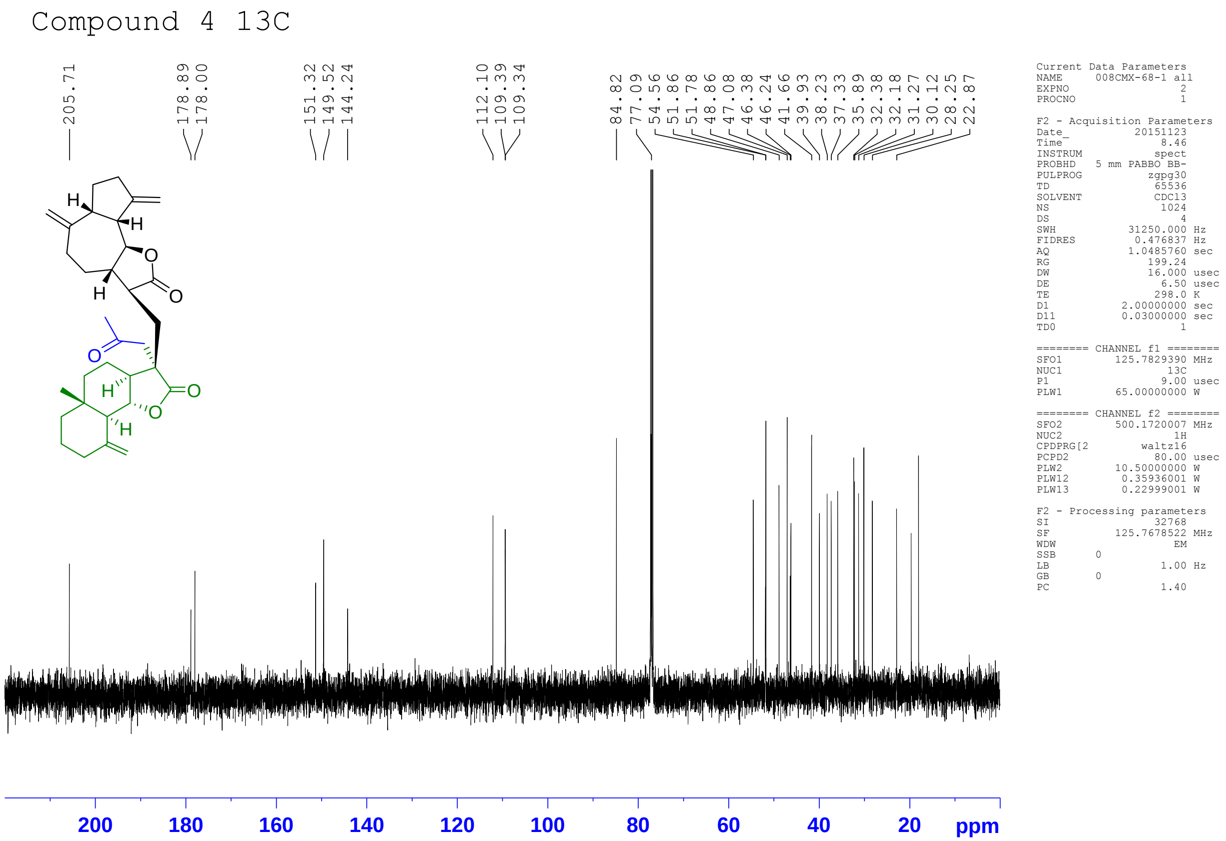

Figure S50. ^{13}C NMR spectra of Vlasouliolide D (**4**) in $CDCl_3$

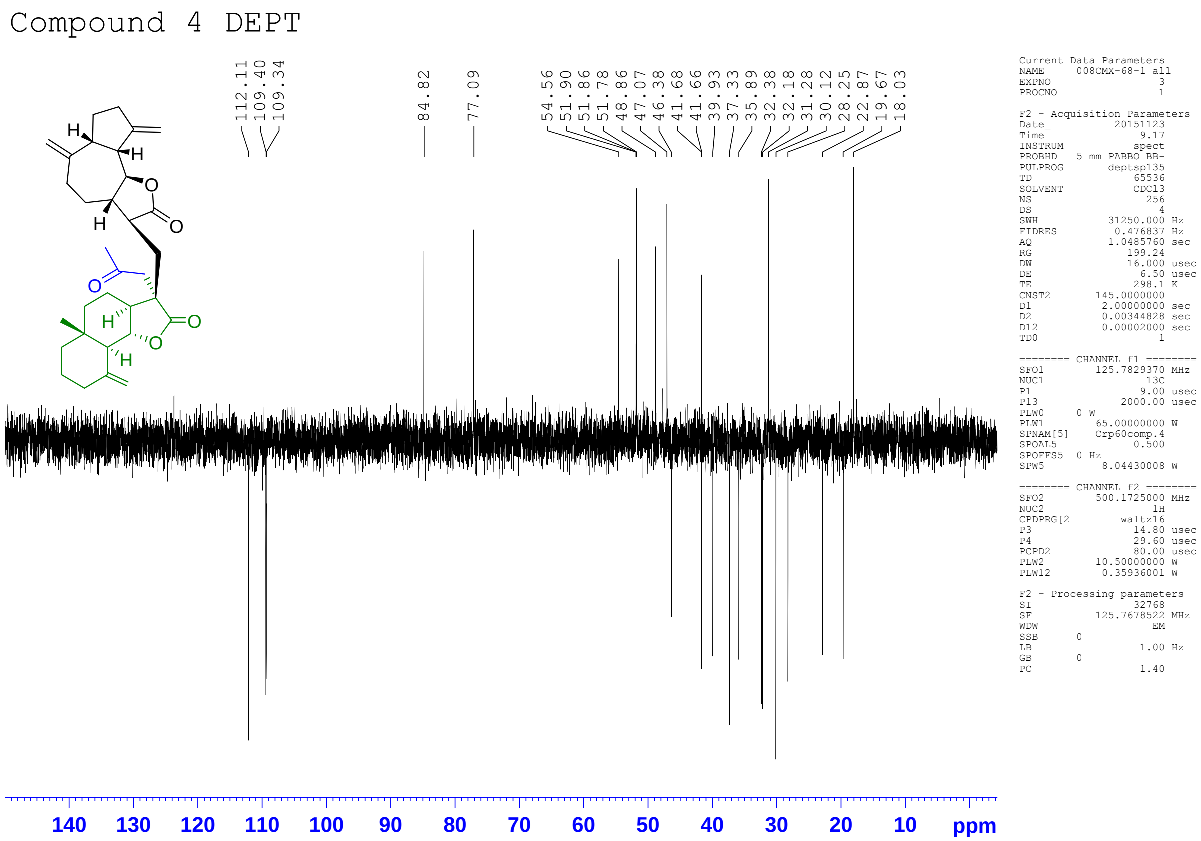

Figure S51. DEPT spectra of Vlasouliolide D (**4**) in $CDCl_3$

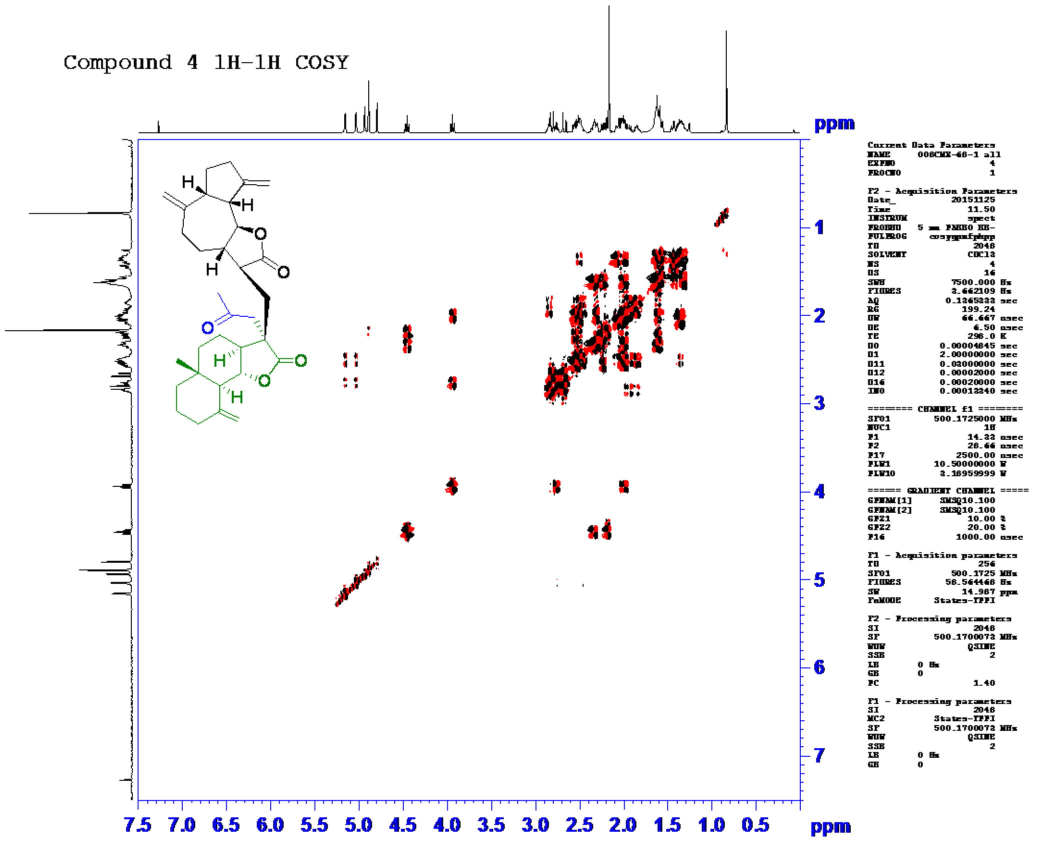

Figure S52. ^{1}H-^{1}H COSY spectrum of Vlasouliolide D (**4**) in $CDCl_3$

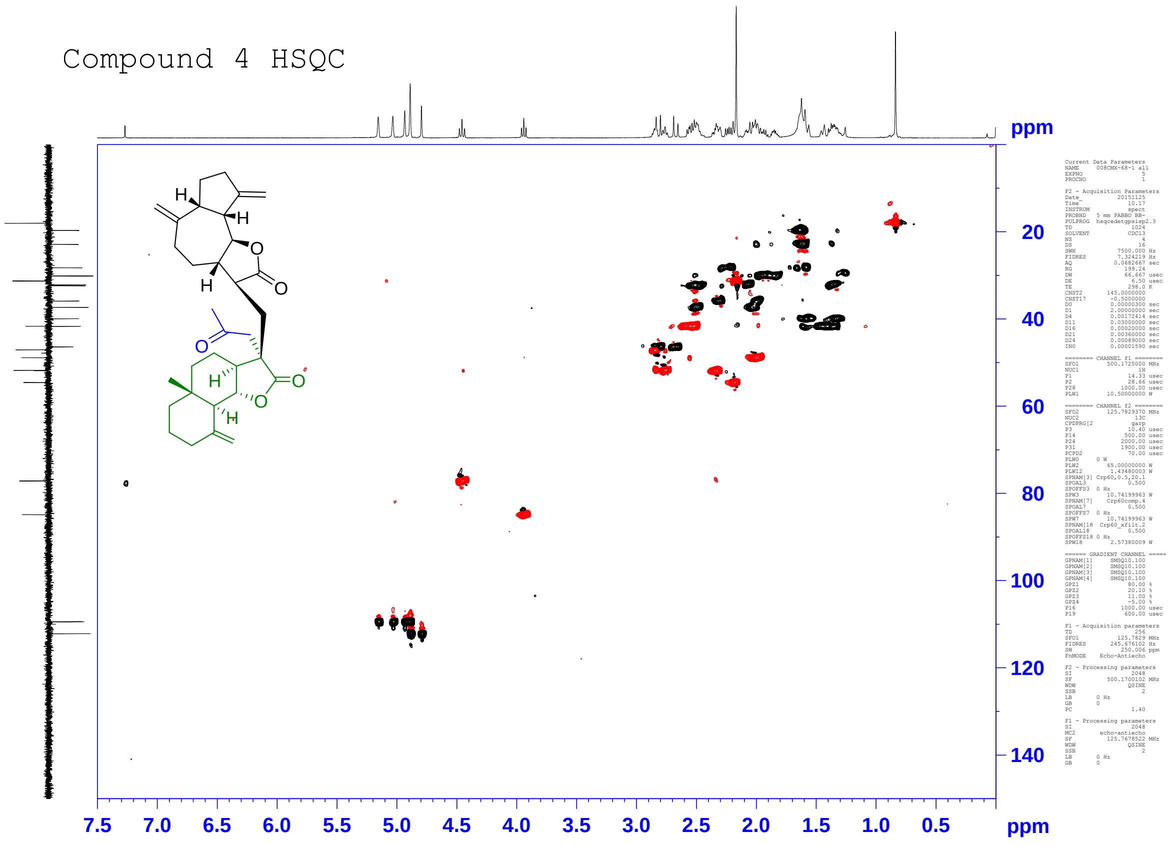

Figure S53. HSQC spectrum of Vlasouliolide D (**4**) in $CDCl_3$

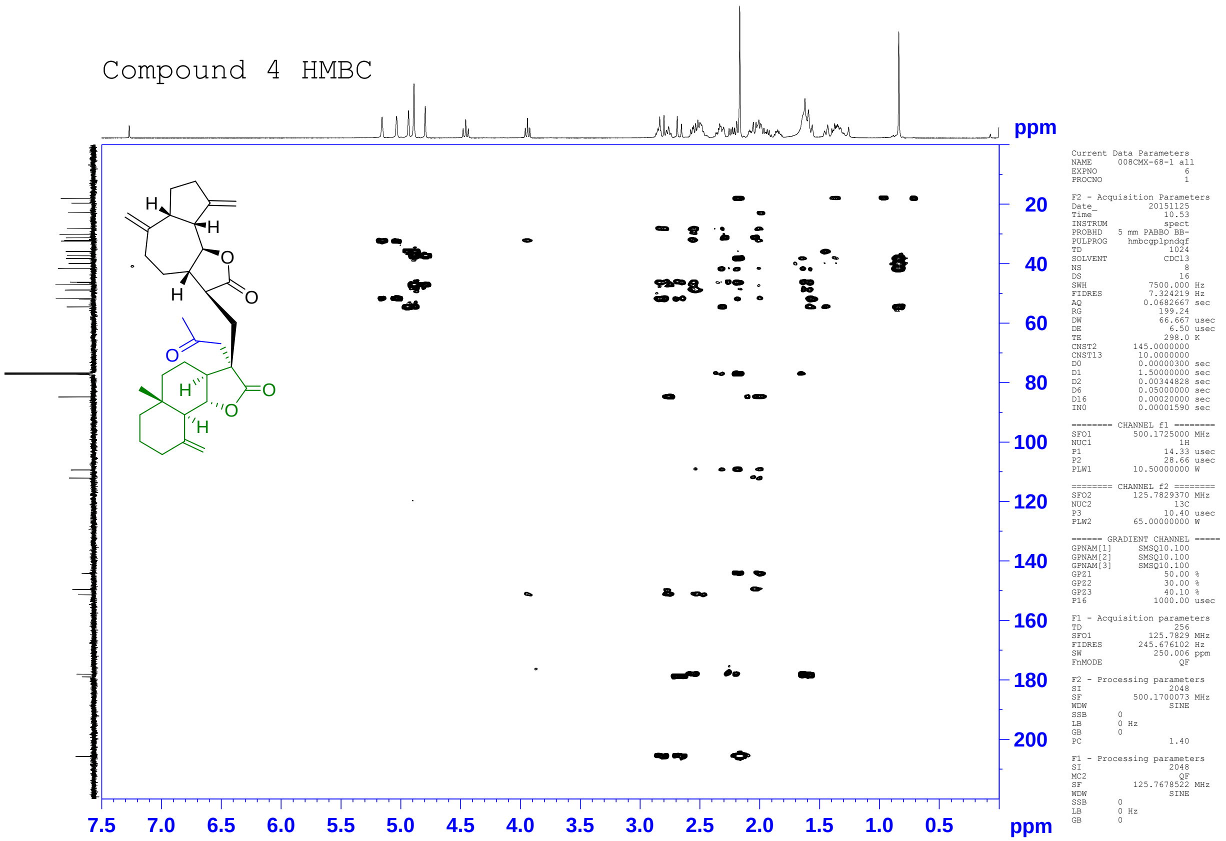

Figure S54. HMBC spectrum of Vlasouliolide D (**4**) in $CDCl_3$

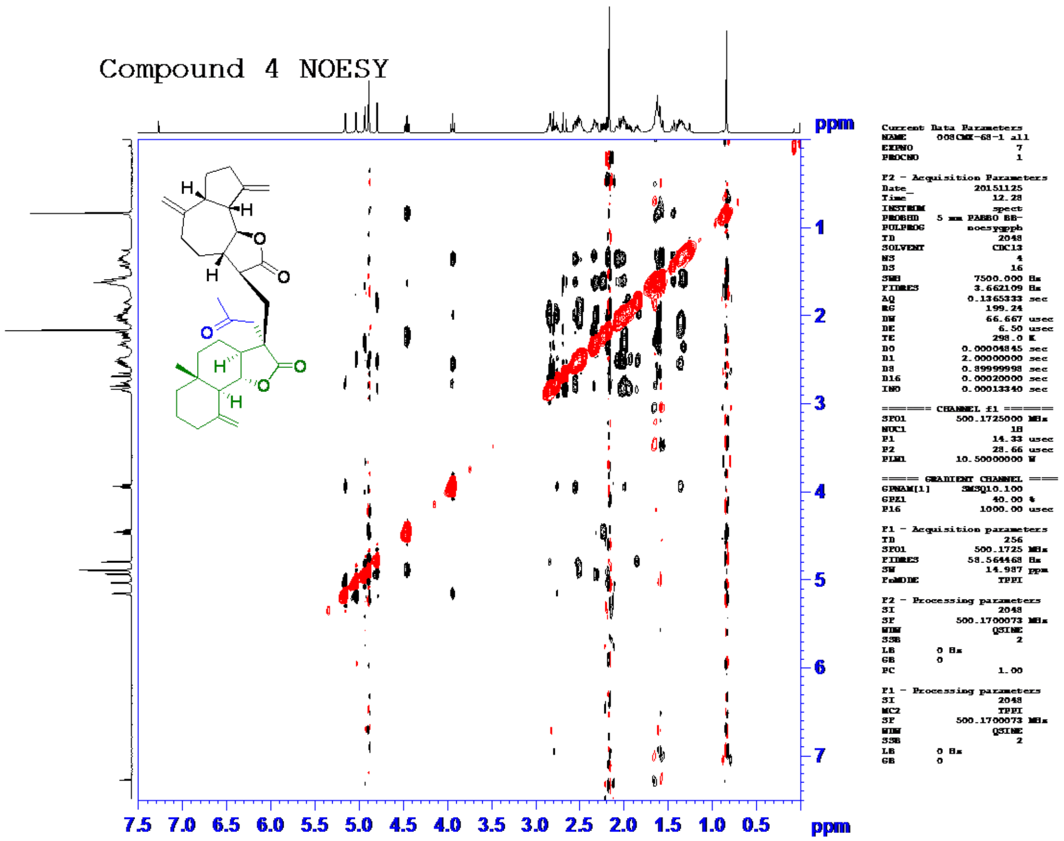

Figure S55. NOESY spectrum of Vlasouliolide D (**4**) in $CDCl_3$

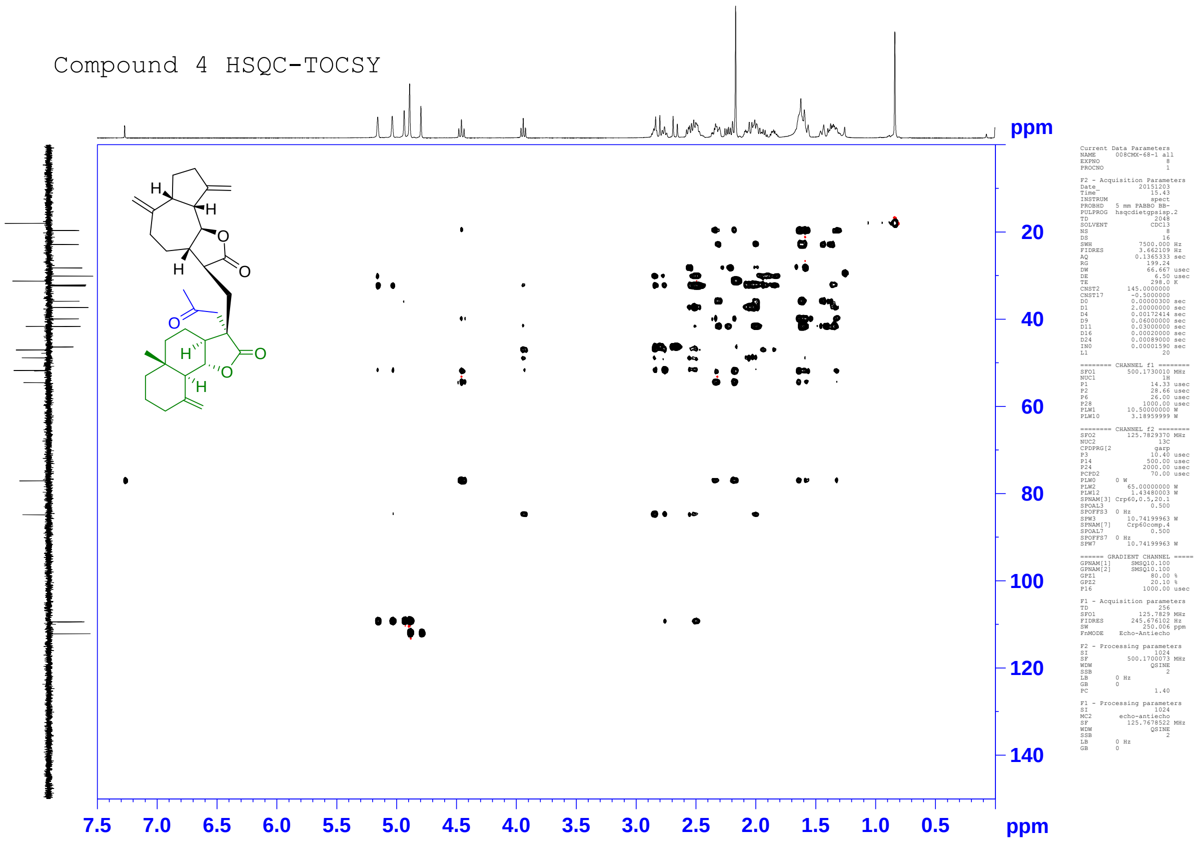

Figure S56. HSQC-TOCSY spectrum of Vlasouliolide D (**4**) in $CDCl_3$

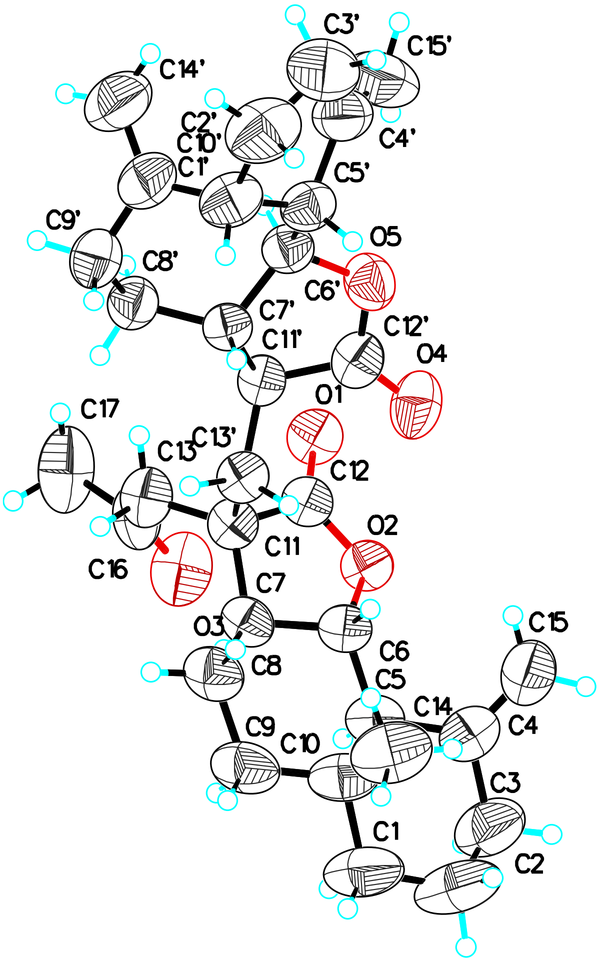

Figure S57. X-ray structure of Vlasouliolide D (**4**)

Table S6. Crystal data and structure refinement for Vlasouliolide D (**4**)

Identification code	dm15940	
Empirical formula	C32 H42 O5	
Formula weight	506.65	
Temperature	293.15 K	
Wavelength	1.54178 Å	
Crystal system	Orthorhombic	
Space group	P 21 21 2	
Unit cell dimensions	a = 12.2212(5) Å	α= 90 °.
	b = 32.2626(14) Å	β= 90 °.
	c = 7.4721(3) Å	γ = 90 °.
Volume	2946.2(2) $Å^3$	
Z	4	
Density (calculated)	1.142 Mg/m^3	
Absorption coefficient	0.601 mm^{-1}	
F(000)	1096	
Crystal size	0.22 x 0.2 x 0.15 mm^3	
Theta range for data collection	2.739 to 69.484 °.	
Index ranges	-14<=h<=14, -38<=k<=39, -8<=l<=9	
Reflections collected	26386	
Independent reflections	5396 [R(int) = 0.0465]	
Completeness to theta = 67.679 °	99.8 %	
Absorption correction	Semi-empirical from equivalents	
Max. and min. transmission	0.7532 and 0.6067	
Refinement method	Full-matrix least-squares on F^2	
Data / restraints / parameters	5396 / 0 / 336	
Goodness-of-fit on F^2	1.077	
Final R indices [I>2sigma(I)]	R1 = 0.0537, wR2 = 0.1323	
R indices (all data)	R1 = 0.0647, wR2 = 0.1396	
Absolute structure parameter	0.14(11)	
Extinction coefficient	n/a	
Largest diff. peak and hole	0.120 and -0.170 e.$Å^{-3}$	

Biological activity assay

Table S7. IC_{50} values (μM) of **1-4** from *Vladimiria souliei* against LPS-induced nitric oxide (NO) production

Compound	1	2	3	Mean±SD IC_{50} (μM)
**1**	1.11	0.98	1.34	1.14±0.18
**2**	2.14	3.45	2.01	2.53±0.80
**3**	1.25	1.76	1.69	1.57±0.28
**4**	2.51	3.14	3.91	3.19±0.70
Aminoguanidine[a]	3.22	3.35	3.51	3.36±0.14

[a]Aminoguanidine was positive control.

Table S8. Cytotoxicity Assay of **1-4** from *Vladimiria souliei* (Mean±SD, n=3)

Conc. (μM/L)	**1**	**2**	**3**	**4**
20	60.24±9.44	96.45±8.45	76.45±8.34	88.35±9.05
10	65.89±4.33	94.67±4.55	84.06±9.30	94.56±8.04
5	84.12±7.98	96.39±7.45	85.56±8.89	100.02±9.46
2.5	90.12±10.45	90.56±8.50	93.45±8.83	99.35±7.49
1.25	92.14±8.91	91.34±8.08	95.20±10.34	100.35±7.57
0.625	98.41±9.81	97.45±10.34	100.31±8.44	97.24±9.47
0.3125	98.34±8.59	99.35±10.01	97.47±7.34	99.46±7.94

Compounds **1-4** were not exhibited significantly cytotoxic at the concentrations required for inhibition NO production (as determined by MTT assay).
