# Supplementary material for: Vlasouliolides A-D, four rare C17/C15 sesquiterpene lactone dimers with potential anti-inflammatory activity from Vladimiria souliei
Source: Sci Rep. 2017 Mar 3;7:43837. doi: 10.1038/srep43837 (PMC5335558; doi:10.1038/srep43837)
Supplement: Supplementary Dataset 2 [file srep43837-s3.zip › checkcif/checkCIF compound 2.pdf]

checkCIF (basic structural check) running

Checking for embedded fcf data in CIF ...
Found embedded fcf data in CIF. Extracting fcf data from uploaded CIF, please wait . . . .

checkCIF/PLATON (basic structural check)

Structure factors have been supplied for datablock(s) cu_dm15891_0m

THIS REPORT IS FOR GUIDANCE ONLY. IF USED AS PART OF A REVIEW PROCEDURE FOR PUBLICATION, IT SHOULD NOT REPLACE THE EXPERTISE OF AN EXPERIENCED CRYSTALLOGRAPHIC REFEREE.

No syntax errors found.
Please wait while processing ....
Structure factor report

CIF dictionary
Interpreting this report

Datablock: cu_dm15891_0m

```
Bond precision:     C-C = 0.0074 A            Wavelength=1.54178
Cell:        a=11.1725(3)   b=14.2312(4)   c=35.3832(10)
             alpha=90       beta=90        gamma=90
Temperature: 296 K
```

	Calculated	Reported
Volume	5625.9(3)	5625.9(3)
Space group	P 21 21 21	P 21 21 21
Hall group	P 2ac 2ab	P 2ac 2ab
Moiety formula	C32 H40 O5	C32 H40 O5
Sum formula	C32 H40 O5	C32 H40 O5
Mr	504.64	504.64
Dx,g cm-3	1.192	1.192
Z	8	8
Mu (mm-1)	0.629	0.629
F000	2176.0	2176.0
F000'	2182.36	
h,k,lmax	13,17,43	13,17,43
Nref	10692[5947]	10295
Tmin,Tmax	0.941,0.969	0.618,0.753
Tmin'	0.939	

```
Correction method= # Reported T Limits: Tmin=0.618
Tmax=0.753 AbsCorr = MULTI-SCAN
Data completeness= 1.73/0.96   Theta(max)= 70.111
R(reflections)= 0.0570( 7800)     wR2(reflections)= 0.1664( 10295)
```

```
S = 1.070             Npar= 669
```

The following ALERTS were generated. Each ALERT has the format
test-name_ALERT_alert-type_alert-level.
Click on the hyperlinks for more details of the test.

Alert level C

PLAT220_ALERT_2_C Large Non-Solvent C Ueq(max)/Ueq(min) Range 3.6 Ratio
PLAT220_ALERT_2_C Large Non-Solvent O Ueq(max)/Ueq(min) Range 3.5 Ratio
PLAT241_ALERT_2_C High 'MainMol' Ueq as Compared to Neighbors of C3 Check
And 7 other PLAT241 Alerts

PLAT241_ALERT_2_C High 'MainMol' Ueq as Compared to Neighbors of C3' Check

PLAT241_ALERT_2_C High 'MainMol' Ueq as Compared to Neighbors of C9 Check

PLAT241_ALERT_2_C High 'MainMol' Ueq as Compared to Neighbors of C2A Check

PLAT241_ALERT_2_C High 'MainMol' Ueq as Compared to Neighbors of C3A Check

PLAT241_ALERT_2_C High 'MainMol' Ueq as Compared to Neighbors of C3B Check

PLAT241_ALERT_2_C High 'MainMol' Ueq as Compared to Neighbors of C9A Check

PLAT241_ALERT_2_C High 'MainMol' Ueq as Compared to Neighbors of C9B Check

PLAT242_ALERT_2_C Low 'MainMol' Ueq as Compared to Neighbors of C4 Check
And 8 other PLAT242 Alerts

PLAT242_ALERT_2_C Low 'MainMol' Ueq as Compared to Neighbors of C4' Check

PLAT242_ALERT_2_C Low 'MainMol' Ueq as Compared to Neighbors of C10 Check

PLAT242_ALERT_2_C Low 'MainMol' Ueq as Compared to Neighbors of C2"A Check

PLAT242_ALERT_2_C Low 'MainMol' Ueq as Compared to Neighbors of C4A Check

PLAT242_ALERT_2_C Low 'MainMol' Ueq as Compared to Neighbors of C4B Check

PLAT242_ALERT_2_C Low 'MainMol' Ueq as Compared to Neighbors of C6A Check

PLAT242_ALERT_2_C Low 'MainMol' Ueq as Compared to Neighbors of C10A Check

PLAT242_ALERT_2_C Low 'MainMol' Ueq as Compared to Neighbors of C10B Check

PLAT340_ALERT_3_C Low Bond Precision on C-C Bonds .... 0.00744 Ang.
PLAT790_ALERT_4_C Centre of Gravity not Within Unit Cell: Resd. # 1 Note
C32 H40 O5
PLAT911_ALERT_3_C Missing # FCF Refl Between THmin & STh/L= 0.600 11 Report

---

Alert level G

PLAT720_ALERT_4_G Number of Unusual/Non-Standard Labels .... 32 Note
PLAT790_ALERT_4_G Centre of Gravity not Within Unit Cell: Resd. # 2 Note
C32 H40 O5
PLAT791_ALERT_4_G The Model has Chirality at C1 (Chiral SPGR) R Verify
And 19 other PLAT791 Alerts

PLAT791_ALERT_4_G The Model has Chirality at C1' (Chiral SPGR) R Verify

PLAT791_ALERT_4_G The Model has Chirality at C1A (Chiral SPGR) R Verify

PLAT791_ALERT_4_G The Model has Chirality at C1B (Chiral SPGR) R Verify

PLAT791_ALERT_4_G The Model has Chirality at C5 (Chiral SPGR) R Verify

PLAT791_ALERT_4_G The Model has Chirality at C5' (Chiral SPGR) R Verify

PLAT791_ALERT_4_G The Model has Chirality at C5A (Chiral SPGR) R Verify

PLAT791_ALERT_4_G The Model has Chirality at C5B (Chiral SPGR) R Verify

PLAT791_ALERT_4_G The Model has Chirality at C6 (Chiral SPGR) R Verify

PLAT791_ALERT_4_G The Model has Chirality at C6' (Chiral SPGR) R Verify

PLAT791_ALERT_4_G The Model has Chirality at C6A (Chiral SPGR) R Verify

PLAT791_ALERT_4_G The Model has Chirality at C6B (Chiral SPGR) R Verify

PLAT791_ALERT_4_G The Model has Chirality at C7 (Chiral SPGR) S Verify

PLAT791_ALERT_4_G The Model has Chirality at C7' (Chiral SPGR) S Verify

PLAT791_ALERT_4_G The Model has Chirality at C7A (Chiral SPGR) S Verify

PLAT791_ALERT_4_G The Model has Chirality at C7B (Chiral SPGR) S Verify

PLAT791_ALERT_4_G The Model has Chirality at C11 (Chiral SPGR) R Verify

PLAT791_ALERT_4_G The Model has Chirality at C11' (Chiral SPGR) S Verify

PLAT791_ALERT_4_G The Model has Chirality at C11A (Chiral SPGR) R Verify

PLAT791_ALERT_4_G The Model has Chirality at C11B (Chiral SPGR) S Verify

PLAT912_ALERT_4_G Missing # of FCF Reflections Above STh/L= 0.600 117 Note
PLAT916_ALERT_2_G Hooft y and Flack x Parameter values differ by . 0.10 Check

0 **ALERT level A** = Most likely a serious problem - resolve or explain
0 **ALERT level B** = A potentially serious problem, consider carefully
22 **ALERT level C** = Check. Ensure it is not caused by an omission or oversight
24 **ALERT level G** = General information/check it is not something unexpected

0 ALERT type 1 CIF construction/syntax error, inconsistent or missing data
20 ALERT type 2 Indicator that the structure model may be wrong or deficient
2 ALERT type 3 Indicator that the structure quality may be low
24 ALERT type 4 Improvement, methodology, query or suggestion
0 ALERT type 5 Informative message, check

It is advisable to attempt to resolve as many as possible of the alerts in all categories. Often the minor aler point to easily fixed oversights, errors and omissions in your CIF or refinement strategy, so attention to these fine details can be worthwhile. In order to resolve some of the more serious problems it may be necessary to carry out additional measurements or structure refinements. However, the purpose of your study may justify the reported deviations and the more serious of these should normally be commented upon in the discussion or experimental section of a paper or in the "special_details" fields of the CIF. checkCIF was carefully designed to identify outliers and unusual parameters, but every test has its limitations and alerts that are not important in a particular case may appear. Conversely, the absence of alerts does not guarantee there are no aspects of the results needing attention. It is up to the individual to critically assess their own results and, if necessary, seek expert advice.

Publication of your CIF in IUCr journals

A basic structural check has been run on your CIF. These basic checks will be run on all CIFs submitted for publication in IUCr journals (*Acta Crystallographica*, *Journal of Applied Crystallography*, *Journal of Synchrotron Radiation*); however, if you intend to submit to *Acta Crystallographica Section C* or *E*, you should make sure that full publication checks are run on the final version of your CIF prior to submission.

Publication of your CIF in other journals

Please refer to the *Notes for Authors* of the relevant journal for any special instructions relating to CIF submission.

PLATON version of 19/11/2015; check.def file version of 17/11/2015

Datablock cu_dm15891_0m - ellipsoid plot

Download CIF editor (publCIF) from the IUCr
Download CIF editor (enCIFer) from the CCDC
Test a new CIF entry
