# Supplementary material for: Vlasouliolides A-D, four rare C17/C15 sesquiterpene lactone dimers with potential anti-inflammatory activity from Vladimiria souliei
Source: Sci Rep. 2017 Mar 3;7:43837. doi: 10.1038/srep43837 (PMC5335558; doi:10.1038/srep43837)
Supplement: Supplementary Dataset 2 [file srep43837-s3.zip › checkcif/checkCIF compound 3.pdf]

checkCIF (basic structural check) running

Checking for embedded fcf data in CIF ...
Found embedded fcf data in CIF. Extracting fcf data from uploaded CIF, please wait . .

checkCIF/PLATON (basic structural check)

Structure factors have been supplied for datablock(s) cu_dm16188_0m

THIS REPORT IS FOR GUIDANCE ONLY. IF USED AS PART OF A REVIEW PROCEDURE FOR PUBLICATION, IT SHOULD NOT REPLACE THE EXPERTISE OF AN EXPERIENCED CRYSTALLOGRAPHIC REFEREE.

No syntax errors found.
Please wait while processing ....
Structure factor report

CIF dictionary
Interpreting this report

Datablock: cu_dm16188_0m

```
Bond precision:      C-C = 0.0051 A            Wavelength=1.54178
Cell:         a=9.1953(2)    b=7.6570(2)    c=19.9371(2)
              alpha=90       beta=99.956(2) gamma=90
Temperature:296 K
                   Calculated                      Reported
Volume             1382.60(5)                      1382.60(5)
Space group        P 21                            P 1 21 1
Hall group         P 2yb                           P 2yb
Moiety formula     C32 H42 O5                      C32 H42 O5
Sum formula        C32 H42 O5                      C32 H42 O5
Mr                 506.66                          506.65
Dx,g cm-3          1.217                           1.217
Z                  2                               2
Mu (mm-1)          0.640                           0.640
F000               548.0                           548.0
F000'              549.59
h,k,lmax           11,9,24                         11,8,24
Nref               5201[ 2810]                     3967
Tmin,Tmax          0.962,0.994                     0.564,0.753
Tmin'              0.926
Correction method= # Reported T Limits: Tmin=0.564
Tmax=0.753 AbsCorr = MULTI-SCAN
Data completeness= 1.41/0.76   Theta(max)= 69.439
R(reflections)= 0.0425( 3462)     wR2(reflections)= 0.1108( 3967)
S = 1.042             Npar= 336
```

The following ALERTS were generated. Each ALERT has the format
test-name_ALERT_alert-type_alert-level.
Click on the hyperlinks for more details of the test.

Alert level C

STRVA01_ALERT_4_C Flack test results are ambiguous.
From the CIF: _refine_ls_abs_structure_Flack 0.400
From the CIF: _refine_ls_abs_structure_Flack_su 0.200
PLAT029_ALERT_3_C _diffrn_measured_fraction_theta_full value Low . 0.971 Note
PLAT241_ALERT_2_C High 'MainMol' Ueq as Compared to Neighbors of C3' Check
PLAT242_ALERT_2_C Low 'MainMol' Ueq as Compared to Neighbors of C1" Check
PLAT340_ALERT_3_C Low Bond Precision on C-C Bonds .... 0.00511 Ang.
PLAT411_ALERT_2_C Short Inter H...H Contact H5' .. H15C .. 2.13 Ang.
PLAT911_ALERT_3_C Missing # FCF Refl Between THmin & STh/L= 0.600 61 Report
PLAT915_ALERT_3_C No Flack x Check Done: Low Friedel Pair Coverage 53 %
PLAT978_ALERT_2_C Number C-C Bonds with Positive Residual Density 0 Note

Alert level G

PLAT153_ALERT_1_G The s.u.'s on the Cell Axes are Equal ..(Note) 0.0002 Ang.
PLAT720_ALERT_4_G Number of Unusual/Non-Standard Labels .... 11 Note
PLAT791_ALERT_4_G The Model has Chirality at C1' (Chiral SPGR) R Verify
And 9 other PLAT791 Alerts

PLAT791_ALERT_4_G The Model has Chirality at C5 (Chiral SPGR) S Verify

PLAT791_ALERT_4_G The Model has Chirality at C5' (Chiral SPGR) R Verify

PLAT791_ALERT_4_G The Model has Chirality at C6 (Chiral SPGR) S Verify

PLAT791_ALERT_4_G The Model has Chirality at C6' (Chiral SPGR) R Verify

PLAT791_ALERT_4_G The Model has Chirality at C7 (Chiral SPGR) S Verify

PLAT791_ALERT_4_G The Model has Chirality at C7' (Chiral SPGR) S Verify

PLAT791_ALERT_4_G The Model has Chirality at C10 (Chiral SPGR) R Verify

PLAT791_ALERT_4_G The Model has Chirality at C11 (Chiral SPGR) S Verify

PLAT791_ALERT_4_G The Model has Chirality at C11' (Chiral SPGR) S Verify

PLAT910_ALERT_3_G Missing # of FCF Reflection(s) Below Theta(Min) 1 Note
PLAT912_ALERT_4_G Missing # of FCF Reflections Above STh/L= 0.600 33 Note

0 **ALERT level A** = Most likely a serious problem - resolve or explain
0 **ALERT level B** = A potentially serious problem, consider carefully
9 **ALERT level C** = Check. Ensure it is not caused by an omission or oversight
14 **ALERT level G** = General information/check it is not something unexpected

1 ALERT type 1 CIF construction/syntax error, inconsistent or missing data
4 ALERT type 2 Indicator that the structure model may be wrong or deficient
5 ALERT type 3 Indicator that the structure quality may be low

13 ALERT type 4 Improvement, methodology, query or suggestion
0 ALERT type 5 Informative message, check

It is advisable to attempt to resolve as many as possible of the alerts in all categories. Often the minor alerts point to easily fixed oversights, errors and omissions in your CIF or refinement strategy, so attention to these fine details can be worthwhile. In order to resolve some of the more serious problems it may be necessary to carry out additional measurements or structure refinements. However, the purpose of your study may justify the reported deviations and the more serious of these should normally be commented upon in the discussion or experimental section of a paper or in the "special_details" fields of the CIF. checkCIF was carefully designed to identify outliers and unusual parameters, but every test has its limitations and alerts that are not important in a particular case may appear. Conversely, the absence of alerts does not guarantee there are no aspects of the results needing attention. It is up to the individual to critically assess their own results and, if necessary, seek expert advice.

Publication of your CIF in IUCr journals

A basic structural check has been run on your CIF. These basic checks will be run on all CIFs submitted for publication in IUCr journals (*Acta Crystallographica*, *Journal of Applied Crystallography*, *Journal of Synchrotron Radiation*); however, if you intend to submit to *Acta Crystallographica Section C* or *E* or *IUCrData*, you should make sure that full publication checks are run on the final version of your CIF prior to submission.

Publication of your CIF in other journals

Please refer to the *Notes for Authors* of the relevant journal for any special instructions relating to CIF submission.

PLATON version of 30/03/2016; check.def file version of 30/03/2016

Datablock cu_dm16188_0m - ellipsoid plot

无法显示链接的图像。该文件可能已被移动、重命名或删除。请验证该链接是否指向正确的文件和位置。

Download CIF editor (publCIF) from the IUCr
Download CIF editor (enCIFer) from the CCDC
Test a new CIF entry
